# Pressure injury treatment by intermittent electrical stimulation (PROTECT-2): protocol for a multicenter randomized clinical trial

**DOI:** 10.1186/s13063-024-08085-x

**Published:** 2024-05-10

**Authors:** Chase Donaldson, Marcelo Gama de Abreu, Edward J. Mascha, James Rowbottom, Eric Harvester, Ashish Khanna, Tanmay Sura, Daniel I. Sessler, Fabio Rodriguez Patarroyo, Alper Gulluoglu, Paul Zajic, Utkarsh Chauhan, Hani Essber, Andrea Kurz

**Affiliations:** 1 Department of Intensive Care and Resuscitation, Cleveland Clinic, 9500 Euclid Avenue, Cleveland, OH 44195 USA; 2 Department of Quantitative Health Sciences and Outcomes Research, Lerner Research Institute; Outcomes Research Consortium, Department of Anesthesiology, Hospital Based Care Institute, 9500 Euclid Avenue, Cleveland, OH 44195 USA; 3 Department of Anesthesiology, Section On Critical Care Medicine, Wake Forest University School of Medicine, Atrium Health Wake Forest Baptist Medical Center, Medical Center Boulevard, Winston-Salem, NC 27157 USA; 4 Department of Anesthesiology, 100 Medical Center Blvd, Winston-Salem, NC 27157 USA; 5 Outcomes Research Consortium, Department of Anesthesiology, Hospital Based Care Institute, Cleveland Clinic, 9500 Euclid Avenue, Cleveland, OH 44195 USA; 6 Outcomes Research Consortium, Department of Anesthesiology, Hospital Based Care Institute, Cleveland Clinic, 9500 Euclid Avenue, Cleveland, OH 44195 USA; 7 Department of Anaesthesiology and Intensive Care Medicine, Medical University of Graz, Auenbruggerpl, 5, Graz, 8036 Austria; 8 University of Alberta Medical School, 1-002 Katz Group Centre for Pharmacy and Health Research, Edmonton, AB T6G 2E1 Canada; 9 Departments of General Anesthesiology and Outcomes Research Consortium, Department of Anesthesiology, Hospital Based Care Institute, Cleveland Clinic , 9500 Euclid Avenue, Cleveland, OH 44195 USA

**Keywords:** Pressure ulcer, Electric stimulation therapy, Intensive care units, Wound healing

## Abstract

**Background:**

Pressure ulcers account for a substantial fraction of hospital-acquired pathology, with consequent morbidity and economic cost. Treatments are largely focused on preventing further injury, whereas interventions that facilitate healing remain limited. Intermittent electrical stimulation (IES) increases local blood flow and redistributes pressure from muscle-bone interfaces, thus potentially reducing ulcer progression and facilitating healing.

**Methods:**

The Pressure Injury Treatment by Intermittent Electrical Stimulation (PROTECT-2) trial will be a parallel-arm multicenter randomized trial to test the hypothesis that IES combined with routine care reduces sacral and ischial pressure injury over time compared to routine care alone. We plan to enroll 548 patients across various centers. Hospitalized patients with stage 1 or stage 2 sacral or ischial pressure injuries will be randomized to IES and routine care or routine care alone. Wound stage will be followed until death, discharge, or the development of an exclusion criteria for up to 3 months. The primary endpoint will be pressure injury score measured over time.

**Discussion:**

Sacral and ischial pressure injuries present a burden to hospitalized patients with both clinical and economic consequences. The PROTECT-2 trial will evaluate whether IES is an effective intervention and thus reduces progression of stage 1 and stage 2 sacral and ischial pressure injuries.

**Trial registration:**

ClinicalTrials.gov NCT05085288 Registered October 20, 2021.

**Supplementary Information:**

The online version contains supplementary material available at 10.1186/s13063-024-08085-x.

## Introduction

### Background and rationale

Pressure ulcers constitute a major morbidity in critically ill patients and are consequent to immobilization, deranged tissue perfusion, and poor nutrition all of which contribute to local tissue ischemia and skin and soft tissue breakdown through a reduction in perfusion pressure and impaired healing. The prevalence of pressure ulcers in ICU patients is 16–26% [1, 2]. In the United States, they are responsible for over 60,000 deaths each year, and the treatment of hospital-acquired pressure ulcers costs ~ $12 billion annually [3]. Despite preventive measures, pressure ulcers can develop within hours [4], especially in patients with hypotension, mechanical ventilation, renal replacement therapy, ICU sedation, and exposure to vasopressors [5]. Consequently, hospital-acquired pressure ulcers are about 4 times as common in ICUs than routine care wards [6].

Intermittent electrical stimulation (IES) was developed more than 35 years ago and was designed to increase tissue perfusion. The belief is that enhancing tissue perfusion improves local tissue oxygenation, improving tissue resistance to pressure and other factors that promote ulceration [7]. The contractions produced by IES reduce pressure around ischial tuberosities and distribute pressure to areas at lesser risk of breakdown from sustained loading (sitting or supine) as demonstrated by functional MRI [8]. The device invokes muscle contractions for 10 s every 10 min via surface electrodes, emulating the subconscious adjustments performed by able-bodied individuals in response to discomfort when seated or lying. Animal studies demonstrate that IES reduces internal pressure at bone-muscle interfaces (the hypothesized mechanism for injury development), increases tissue oxygenation in surrounding areas, and reduces or eliminates deep tissue injury in muscles subjected to prolonged loading [7, 9].

Clinical trials of IES have been limited to phase I trials which have demonstrated the safety of the device, ease of use, and patient satisfaction in multiple settings, including the ICU [10, 11]. Studies by Ahmetović et al. and Kane et al. evaluated the device as a preventive measure for pressure ulcers. No subjects in the trial groups developed a pressure ulcer.

Given the proposed mechanisms of IES and its performance in pilot and phase 1 trials, it is possibly that IES will prove an effective treatment for pressure injuries. Specifically, by preventing further injury through the offloading of pressure from bone-muscle interfaces and the increase of local blood flow, tissue that has already been injured may be less likely to progress to higher pressure stages and potentially heal more quickly. However, there is currently no pre-clinical or clinical evidence from adequately powered trials.

### Objectives

We designed the ‘Pressure Injury Treatment by Intermittent Electrical Stimulation (PROTECT-2) study to test the hypothesis that in patients with new or established stage 1–2 sacral and ischial pressure injuries, IES in addition to routine care for sacral and ischial pressure ulcers promotes healing over time better than routine care alone.

## Methods

### Trial design

**Fig. 1:**
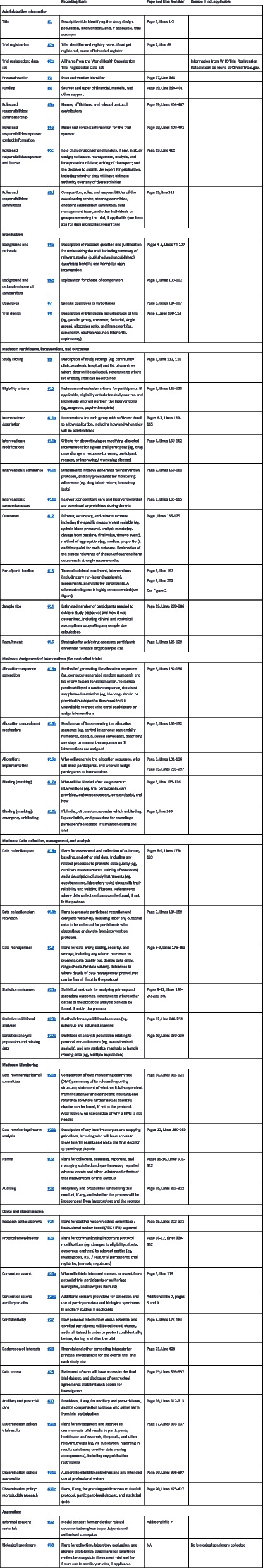
Spirit Schedule of Enrollment, Interventions, and Assessments

PROTECT-2 is a parallel-group, unblinded multi-center randomized clinical trial that will assess whether IES combined with the standard of care (treatment) is superior to the standard of care alone (control). The trial is already IRB approved at all trial sites (US, Austria) and registered at ClinicalTrials.gov (NCT05085288). The SPIRIT checklist and figure for our proposal is provided in Fig. 1 and additional file 1. [12].

### Trial population

We will include a minimum of 548 adult inpatients who meet the following criteria: (1) hospital inpatients, (2) new or established stage 1–2 sacral/ischial pressure injuries, (3) provide written informed consent in English. Consent is given by the patient or their legally authorized representative (LAR). Subjects enrolled via LAR will be themselves consented when able. Consent will be done in person or by telephone by investigators.

Patients with a pacemaker/AICD, active rhabdomyolysis, gluteal skin breakdown, and unstable fractures at risk of displacement by IES will be excluded. Patients with BMI > 40 kg/m^2^ will also be excluded based on concerns about not being able to elicit a reliable contraction of the muscle with the device. Patients with atrial or ventricular wires after cardiac surgery can be enrolled so long as they are not being actively paced or, in the opinion of the treating physician, likely to requiring pacing during the trial. Patients with multiple pressure injuries in higher stages can be enrolled as long as one of the sacral or ischial injuries is stage 1 or 2.

### Patient recruitment

Study personnel will screen the hospital patient lists in the electronic medical record for nursing-documented or wound care nursing-documented pressure ulcers. Some sites also utilize bedside screening in intensive care units.

### Randomization and blinding

Randomization sequences will be generated using the PLAN procedure in the SAS statistical software and will be stratified by clinical site and initial pressure injury stage at point of consent (stage I or II). Furthermore, random sized blocks will be used. Study personnel will randomize patients after enrollment via a central 24-h Interactive Web Randomization System (REDCap). This is an open label study, so no blinding is required. Randomization codes will be maintained until after all the data are collected and analyzed. The patient and study staff will be informed of the randomized allocation.

### Trial intervention

#### Intermittent electrical stimulation device

**Fig. 2:**
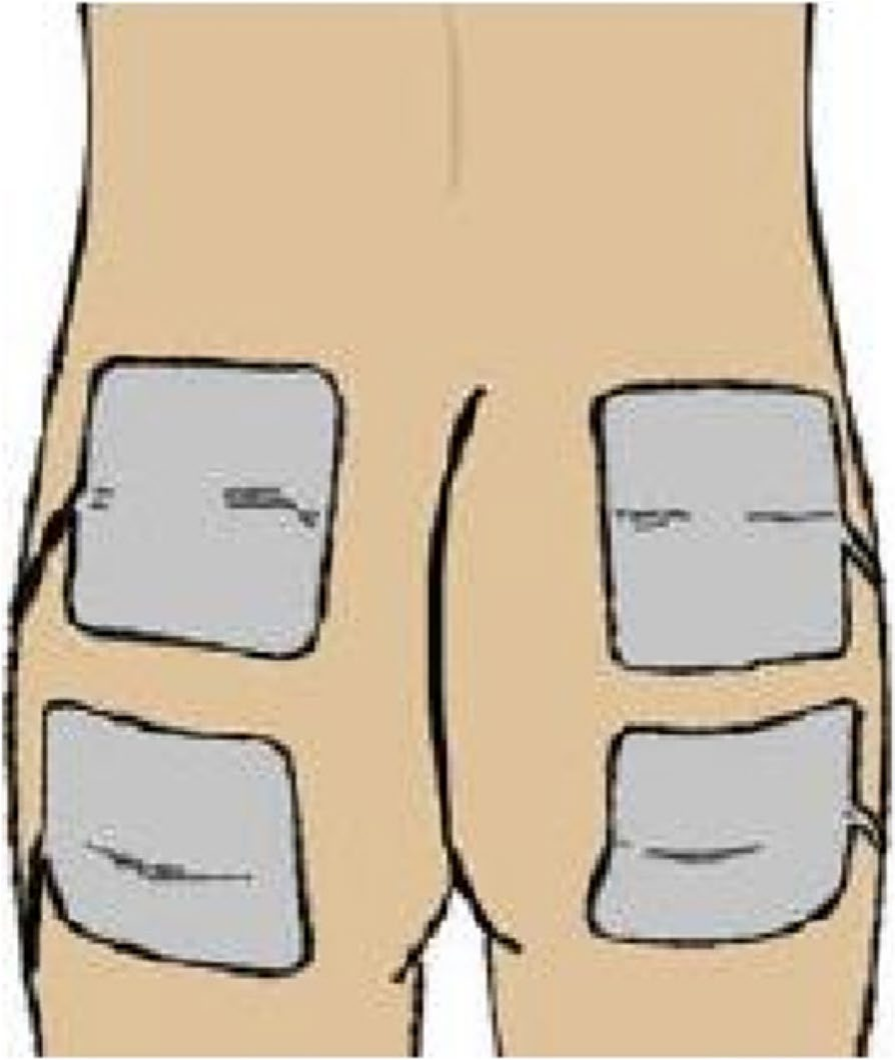
The IES system. Electrodes are placed directly on the skin overlying the gluteal muscles but not directly on the pressure ulcer

The Prelivia System™ is a IES system composed of a simulator (Prelivia, Rehabtronics) and self- adhesive, non-sterile 7.5 by 10 cm surface gel electrodes (Axelgaard Pals Platinum Neurostimulation electrodes, Model 895340-4-40, Fallbrook, CA) applied directly on the skin (Fig. 2).

The device produces charged pulses at 30 Hz. Stimulation intensity is progressively increased until visible muscle contractions of the gluteus maximus muscles are seen, up to a maximum current of 100 mA. Stimulation occurs for 10 s every 10 min. Chosen device parameters approximate those shown to induce pressure redistribution and sustained elevation in tissue oxygenation based on previous studies. Each subject will receive IES consistently 24 h a day from entry and while in the ICU except for brief periods necessary for particular tasks in patient care (ex. dressing changes, operating room needs). While in non-ICU environments, the device will be used while in bed and not participating in physical therapy, mobilization, toileting, and similar activities where no consistent pressure in applied to the sacral area. The study device will be used until discharge, death, patient or LAR request for disenrollment, or the need for surgical or other interventions that preclude the use of IES. Should patients request not to have the study device for a short period of time, this will be notated and they remain in the trial group. If there are patient concerns about contextual use of the device, such as toileting or sleeping, these are discussed with the patient, and should the patient not have the device on per the protocol, this will be noted in the twice daily assessments. If there are medical interventions that require that the sacrum and ischium not be examined, such as unstable open-chested patients, short-term pausing is acceptable and will be documented on the case report form (Additional file 2). If these interventions are longer term or if the patient develops any condition not conducive to the use of the trial device, the device will be discontinued until these conditions are resolved. Research personnel will visit each subject in the study group Monday through Friday to administer a questionnaire to the bedside nurse, ensure proper use of the device, and address participant concerns.

Subjects in the intervention group will receive all other wound care preventatives and treatments that are standard of care. No standard interventions for wound care treatment are prohibited. The standard of care comparator is to isolate the effect of IES in addition to what is the current standard of care.

### Outcome variables

#### Primary

The primary efficacy outcome is the sacral and ischial pressure injury score measured over time.

#### Secondary

Secondary outcomes are time to event endpoints (time to resolution of ulcer, time to worsening of ulcer, time to discharge alive, daily subjective evaluation of ulcer, and mortality).

#### Exploratory

Our first exploratory endpoint will be total hospital cost effectiveness analysis within a subset of the patients. We will measure incremental cost-effectiveness ratios (ICERS) between traditional care and IES interventions of each procedure for the treatment of pressure ulcers, done within a subset of the patients. Furthermore, patient experience with respect to sleep disturbance, distraction or discomfort, and feeling of electric shock will be assessed with a daily questionnaire. Daily assessment of the device area with respect to redness or skin issues will similarly be documented with a daily questionnaire (Additional file 3). The assessors will be research personnel.

### Data collection and management

Data for wound assessment is collected by the electronic medical record as is all other baseline and study data as specified in the case report form (CRF) (Additional file 2). When able, data will be automatically extracted from the EMR. Study personnel at the participating sites record data on CRFs and submit the CRFs through a secure web-based computerized database (i.e., iDataFax). Patients are identified using a unique numeric code, and all patient data are anonymized to ensure patient confidentiality. Data validity checks are programmed in the database and are monitored by data management assistants from the Project Office through multi-level data validation of CRFs.

To promote participant retention and complete follow-up, all data per the protocol will continue to be gathered from the participant as part of the modified intention to treat analysis (i.e., including all randomized patients who receive at least part of the study intervention) should they disenroll unless they request that subsequent data not be gathered. If a patient withdraws from study and also withdraws consent for disclosure of future information, no further evaluations are performed, and no additional data is collected. The sponsor may retain and continue to use any data collected before the withdrawal of consent.

## Statistical methods

### Data analysis

#### Analysis population

All randomized participants who have received any of the study intervention will be included in the primary endpoint analysis using modified intention-to-treat (mITT). The mITT data will be used to evaluate efficacy on the primary and secondary endpoints, and this data will comprise the analysis population.

#### Significance level

The significance level will be 0.05 for all hypothesis testing and will be controlled at 5% across any multiple comparisons using appropriate multiple testing procedures. Two-sided tests for superiority will be used throughout.

For the statistical analysis of clinical course, unstageable injuries will be assigned a score of 3 (equivalent to stage III injuries). If a patient experiences multiple sacral/ischial pressure injury simultaneously, the most severe injury will be used for analysis.

#### Primary outcome

The primary outcome will be sacral and ischial pressure injury assessed with the SPI instrument score (range 0–4) over time. For the statistical analysis of clinical course, unstageable injuries will be assigned a score of 3 (equivalent to stage III injuries). For ulcers that progress to deep tissue injuries (DTI), these will be assigned a score of the previous ulcer stage plus one. If a patient experiences multiple sacral/ischial pressure injury simultaneously, the most severe injury will be used for analysis. The primary outcome will be based on the combined stage 1 and stage 2 ulcer types.

Patients will be assessed twice daily by the bedside nurse for pressure injury location and stage from study entry to discharge from the hospital or at least 30 days, whichever comes first. This is a standard wound care evaluation protocol for nursing. Wound care nursing specialist consultation staging will be used instead of bedside nursing staging should a discrepancy occur. If SPI scores are available after discharge and within the 30 days, those data will be included in the analyses as well, even if the SPI are healed or at the worst stage.

#### Primary outcome analyses

We will assess the efficacy of an IES system added to the standard care versus standard wound care on sacral and ischial pressure injury scores measured over time using a generalized mixed effects ordinal regression cumulative logit model which considers treatment and time (categorical) as fixed effects and subject as a random effect and accounts for (1) the ordinal nature of the data and (2) the within-subject correlation over time. The model will allow differing number of measurements and lengths of follow-up for patients but will assume that shorter follow-up or dropout is largely at random and not due to either improvement or worsening of SPI symptoms. We will also assess the treatment-by-time interaction to assess whether the treatment effect is consistent over time. Given a significant interaction, we will assess the treatment effect at various time points, although the primary analysis will be the treatment effect collapsed over time.

Results will be reported as a proportional odds ratio which estimates the odds of an IES-treated patient having a higher (worse) score than a randomly chosen standard care patient. For example, an odds ratio of 0.6 would mean the treatment was associated with an estimated 40% lower odds of an EIS-treated patient having a higher score compared to standard care; correspondingly, a treated patient has a 40% higher odds of having a lower (better) score compared to a standard care patient.

#### Missing data methods

Study personnel will go to great lengths to avoid missing outcomes data. In any case, missing data in the analyses will be assessed using multiple imputation with chained equations (MICE, also known as fully conditional specification) [13], in which predictions for missing data points will be made using regression models containing all available baseline and outcome variables. We will use 100 MICE iterations and then average predictions for missing values. Using this methodology, even data that is originally missing not at random (MNAR) may effectively be missing at random (MAR) since the predictions utilize information from many variables which might represent the reason(s) for missingness.

#### Displaying of results/treatment effect

We will display the distribution of scores for treatment and control over time. We will also display a forest plot of odds ratios over time and the cumulative/aggregate odds ratio collapsed over time.

### Alternative statistical models and sensitivity analyses

First, if the generalized mixed effects model does not converge or is deemed not sufficient, an analogous generalized estimating equation (GEE) model will be used in which the correlation is adjusted for using the R matrix (within-subject correlation), either unstructured or autoregressive (AR (1)). Second, a recurrent time to event model with ordinal outcome (the continuation ratio approach) [14] will be fit. This approach models the risk for the *j*th event (e.g., a certain category of pressure injury at a specific f/up measurement) ignoring any previous events, but accounting for subject as random effect, and can be modeled using an event- stratified proportional hazards regression model.

#### Treatment effect heterogeneity

We will assess treatment effect (IES device versus control) heterogeneity for the primary outcome across levels of various baseline factors (Additional file 4) using tests for interaction (treatment-by-baseline interaction). Primary result will be the interaction *P*-value and corresponding estimate of the interaction effect. We will also test and report the treatment effect and confidence interval within levels of each factor.

### Secondary endpoint analyses

We will assess the treatment effect of IES vs standard care on time to event outcomes (time to resolution of ulcer, time to worsening of ulcer, time to discharge alive, and mortality) using Cox proportional hazards regression and reporting results as hazard ratio and 95% confidence interval, with the proportional hazards assumption tested using the treatment-by-log(time) interaction as well as graphical displays of the hazard of the outcome over time for the overlayed treatment groups. Kaplan–Meier analyses with 95% confidence bands and the log-rank test will also be used.

#### Exploratory endpoints

We will assess cost effectiveness of IES vs standard care within a subset of the patients studied. We will measure incremental cost-effectiveness ratios (ICERS) between traditional care and IES interventions in the treatment of pressure ulcers. Safety assessment is as follows: results from each of the 5 possible side effects listed on Patient and Assessor Questionnaire will be summarized as (1) proportion of patients ever having the event across their follow-ups, (2) severity grade across follow-ups for those having the event, and (3) severity grade for all patients, assigning a 0 to those without the event. Severity grade will be summarized using median [quartiles] and mean (SD). Confidence intervals within and between randomized groups will be estimated using bootstrap resampling with replacement to account for within-subject correlation across measurements.

#### Internal pilot study to re-assess assumptions on variability and correlation

At both the first and second interim analyses, we will reassess the following assumptions used in sample calculations: within-subject correlation over time, mean # measurements per subject, control group proportions across stages. Reassessing these “nuisance” parameters will not affect the type I error but may result in increasing the maximum planned sample size. We will not reconsider the treatment effect of interest.

### Interim analysis

Interim analyses will be performed at each 25% of the planned enrollment using a group sequential design assessing the treatment effect on the primary outcome for efficacy and futility. We will use a gamma spending function with gamma parameters − 4 for efficacy and − 1 for futility. Stopping boundaries will be statistically non-binding; statistical accommodation for this option was made in the sample size calculations. The decision whether to stop a study at any interim analysis would be made by the DSMB, which would consider not only the statistical boundaries but other information as well. For example, if a futility or efficacy boundary was crossed at the first look, the DSMB might consider continuing the trial to obtain a more precise estimate of the treatment effect.

All data and results will be submitted to the data and safety monitoring board (DSMB) for review on an A versus B basis unless the DSMB requests to be unblinded. The DSMB is made up of physicians from multiple trial sites and a statistician (Additional file 5). The committee will assess any possible safety concerns, efficacy, and futility and advise if discontinuation is warranted and will meet at least yearly. DSMB members are independent from the sponsor and report to and communicate with the IRB and the steering committee.

### Sample size considerations

Sample size calculations are based on the primary outcome of pressure ulcer score (0–4), measured over time within a patient and assuming a statistical model (mixed effects ordinal regression) which accounts for the within-subject correlation over the repeated measurements. We designed the study to have 90% power at the 0.05 significance level to detect a treatment effect as large or larger than the effect seen in Table 1 (A) (for the combined starting stages I and II). Sample size was estimated based on applying a design effect to a standard 2-group comparison for independent data (i.e., the Wilcoxon Rank Sum test); it incorporates the number of measurements per patient (m) and an assumed intraclass correlation (ICC) to account for within-subject correlation (i.e., multiply standard sample size calculation by 1 + (m-1)ICC) [15].

**Table 1:** Minimal clinically important differences in pressure ulcer score

**A. Combined stages I and II** ***N* = 586 fixed, *assuming single measurement* per patient** ***N* = 668 with interim analysis adjustment**	**Pressure ulcer score (row percent)**				
	**0**	**I**	**II**	**III**	**IV**
**Intervention**	**82.5**	**2.5**	**12.5**	**2.5**	**0**
**Control**	**72.5**	**2.5**	**15**	**5**	**5**
**B. Patients starting at stage I** ***N* = 548 fixed (620 with interims)**					
	**0**	**I**	**II**	**III**	**IV**
**Intervention**	**85**	**5**	**7.5**	**2.5**	**0**
**Control**	**75**	**5**	**10**	**5**	**5**
**C. Patients starting at stage II** ***N* = 616 fixed (697 with interims)**					
	**0**	**I**	**II**	**III**	**IV**
**Intervention**	**80**	**0**	**17.5**	**2.5**	**0**
**Control**	**70**	**0**	**20**	**5**	**5**

Hypothetical percent in each pressure ulcer score category by treatment arm, for smallest clinically important effects: overall (A), for stage I (B), and stage II (C)

Table 2 gives total sample size for the combined stages in Table 1 (A), varying the number of measurements per subject (10, 20, 40) and varying ICC (0.2, 0.5, 0.8). The ICC is expected to be relatively high, so the recommended minimum sample size is 548 total (274/gp) for the overall treatment effect combining stages 1 and 2. However, since sufficient power is desired for each of the 2 starting strata, stage I and stage II, roughly the same sample size (548) should be achieved for each strata (see Table 2 for stage I and stage II strata). Therefore, a total maximum (accounting for interim analyses) sample size of approximately 1100 patients was recommended for this trial.

**Table 2:** Sample size for varying # measurements and ICC. Data = *N* total. Starting with *N* = 668 total observations (Table 1 (A)), what is the required *N* subjects accounting for ICC and expected number of measurements/subject?

Patient sample	*N* measurements per patient	ICC		
		**.2**	**.5**	**.8**
**Combined stages **Table 1** (A) effect**	**10**	*188*	*368*	*548*
	**20**	*162*	*351*	*542*
	**40**	*148*	*344*	*538*
**Stage I start **Table 1** (B) effect**	**10**	174	342	510
	**20**	150	326	504
	**40**	138	318	500
**Stage II start **Table 1** (C) effect**	**10**	196	384	572
	**20**	168	366	564
	**40**	154	358	562

*N* = 668 assumed 1 measurement per patient (Table 1)

*ICC* intraclass correlation

### Trial organization

The Cleveland Clinic Department of Outcomes Research is the coordinating center for this trial and is responsible for the central randomization, trial database, data consistency checks, data analyses, and coordination of participating centers worldwide.

The group responsible for trial steering included the primary investigator from three of the research sites, a statistician from the coordinating center, two wound care nursing specialists from two of the research sites, and the chairmen of the Departments of Research from three of the research sites.

The endpoint adjudication committee members included the coordinating center PI, the statistician, and the chairman of the coordinating center research department.

The data management team included a statistician, a member of the database and data quality control arm of the coordinating center research department, and a research fellow. Day-to-day trial conduct is undertaken by research personnel to include fellows and nurses, the primary investigator from each site, two wound care nurses from the coordinating center, the database manager (weekly), and the statistician (weekly).

No stakeholder or public involvement group was utilized for the design of this trial.

## Adverse event reporting

### Reporting, recording, and follow-up of adverse events

The investigator will assess the relationship between protocol treatment and the occurrence of AEs, and this assessment will be recorded in the database for adverse events. The key potential adverse events of interest are restricted to skin irritation/allergies, and these skin manifestations will be scored with the International Common Terminology Criteria for Adverse Events (CTCAE), version 5.0, for adverse event reporting. Due to the non-systemic nature of the intervention, and the complexity of inpatient care and number of concurrent therapies being administered to this population, no other adverse events will be collected or reported. The investigator will follow each adverse event of interest until the event has resolved to baseline grade or better or is assessed as stable by the investigator or until the patient is discharged from hospital. During the study period, resolution of adverse events (with dates) should be documented on the adverse event electronic case report form (eCRF) and the patient’s medical record to facilitate source data verification. If, after follow-up, return to baseline status or stabilization cannot be established, an explanation should be recorded on the adverse event eCRF. No renumeration or compensation is provided to participants.

### Frequency and plan for monitoring trial conduct

Monitoring of the trial conduct will be conducted to minimize the number of errors and missing data and, consequently, to generate an accurate database for analysis. Two independent monitors are installed by the sponsor to perform study monitoring. Remote monitoring will be performed to signal early aberrant patterns, issues with consistency, credibility, and other anomalies. On-site monitoring will be conducted by the sponsor in all sites after the first and the third interim analyses to control the presence and completeness of the research dossier and the informed consent forms. Source data checks will be performed in the files of 25% of the patients after completion of the trial.

## Ethical considerations

This trial is conducted in compliance with the protocol, the Declaration of Helsinki, the International Consensus on Harmonization—Good Clinical Practice (ICH-GCP), and all applicable laws and regulations of the countries in which the study is performed. Before sites start recruiting patients, the local investigators must have written and dated approval/favorable opinion from the Institutional Review Board/Independent Ethics Committee (IRB/IEC) for the protocol, consent form, subject recruitment materials/process, and, where applicable, approval by the participating countries competent authority (CA) in accordance with local laws and regulations (Additional file 6). Amendments to the protocol also require IRB/IEC and/or CA approval, where applicable and relevant changes are made to the ClinicalTrials.gov website. All data are stored on a central encrypted, high-security computer system and kept strictly confidential.

## Dissemination

Our dissemination plan includes presentation at national and international conferences and publications in peer reviewed high-impact journals.

## Discussion

Pressure ulcers are associated with high national healthcare costs [3] (3) and are associated with an increased mortality [16]. Despite many preventative measures that have been developed with varying success, few treatments apart from routine wound care practices have been developed. With its mechanisms of increasing local blood flow and redistributing pressure away from muscle-bone interfaces, IES has the potential to facilitate healing and retard progression of pressure injuries. The mechanism would also have significant physiologic plausibility for the prevention of pressure ulcers in high-risk patients, but given the relatively low incidence of pressure ulcers in our analysis, the number of patients required in a trial attempting to demonstrate whether the device can prevent pressure ulcers was prohibitive. Given the burden of pressure ulcers on hospital systems, evaluating the device’s utility for acute care hospital-based treatment of new or established pressure ulcers is necessary.

The device used for this trial has been used in animal trials and small human trials evaluating its efficacy. We opted for testing the impact of electrodes placed around the pressure ulcer overlying the gluteus maximus muscle given its being a large muscle group with the potential to recruit increased blood flow to the area around the ulcer. The specific settings used in terms of frequency and amperage range were initially determined by preclinical data and were also used in the animal and human studies conducted before PROTECT-2.

Limitations for the interpretation of the results of this study do exist. Besides electrical stimulation of the gluteal muscles, other factors may have effect on the progression and healing of ulcers including contemporary wound care treatments, different national standards of treatment, different hospital protocols, and differences between ICU and non-ICU care. However, these findings will not bias the findings of the study given the multicentered, international nature of the trial, and testing for interaction for ICU vs. non-ICU enrollment will be done. In addition, combining stage 1 and stage 2 ulcers for the primary analysis will not enable us to assess whether the IES device may be more effective for 1 stage of ulcer versus stage 2. Nevertheless, in secondary analyses, we will test the interaction, although underpowered, between treatment and baseline stage as well as report the effect for each stage separately.

Much attention has been paid to safety in the PROTECT-2 trial. Accordingly, data and patient safety during the trial is closely monitored by a DSMB, whose members have been chosen due to their expertise in clinical research. The research electronic data capture will be used for building the database within a secure system and allowing access to the eCRF as well as randomization of patients into groups within one single platform for participation sites. Importantly, data on side effects as well as patient and caregiver perceptions of skin health and sleep hygiene will be collected and used to enhance the description of the stimulation device.

In summary, the PROTECT-2 trial is designed to establish the efficacy of IES for treatment of stage 1–2 sacral and ischial pressure injuries in hospitalized patients.

## Trial progress

This paper is based on the most recent version of the study protocol (i.e., v5.0, 2023-12-29). The first patient was randomized on June 6, 2022. As of December 30, 2023, we have recruited 176 patients across 3 centers in 2 countries. The first interim analysis based on recruitment is anticipated to be completed January 2024.

### Supplementary Information

**Additional file 1.** SPIRIT Checklist.

**Additional file 2.**Case report form (CRF).

**Additional file 3.** Patient and Assessor Questionnaire. Questions for patients based upon their experience as well as the assessor based on their patient evaluation.

**Additional file 4.** Baseline factors for treatement heterogeneity.  

**Additional file 5.** Date and safety monitoring board (DSMB). A list of all members of the DSMB.   

**Additional file 6.** IRB approval/ethical approval.

**Additional file 7.** Funding documentation.

**Additional file 8.**Informed consent materials.

## Data Availability

All institutions and investigators participating in the trial will have access to the final dataset and will be able to freely publish and disseminate study results. The datasets analyzed during the current study and statistical code are available from the corresponding author on reasonable request, as is the full protocol.

## Abbreviations

CA: Competent authority
CTCAE: International Common Terminology Criteria for Adverse Events
CRF: Case report form
DSMB: Data and safety monitoring board
GEE: Generalized estimating equation
IRB/IEC: Institutional review board/independent ethics committee
ICC: Intraclass correlation
ICERS: Incremental cost-effectiveness ratios
ICH-GCP: International Consensus on Harmonization – Good Clinical Practice
IES: Intermittent electrical stimulation
LAR: Legally authorized representative
PH: Proportional hazard

## References

[1] Labeau SO, Afonso E, Benbenishty J, Blackwood B, Boulanger C, Brett SJ (2020). Prevalence, associated factors and outcomes of pressure injuries in adult intensive care unit patients: the DecubICUs study. Intensive Care Med.

[2] Woodbury MG, Houghton PE (2004). Prevalence of pressure ulcers in Canadian healthcare settings. Ostomy Wound Manage.

[3] Zulkowski K, Langemo D, Posthauer ME (2005). Coming to consensus on deep tissue injury. Adv Skin Wound Care.

[4] Gefen A (2008). How much time does it take to get a pressure ulcer? Integrated evidence from human, animal, and in vitro studies. Ostomy Wound Manage.

[5] Lima Serrano M, González Méndez MI, Carrasco Cebollero FM, Lima Rodríguez JS (2017). Risk factors for pressure ulcer development in intensive care units: a systematic review. Med Intensiva.

[6] Coyer F, Miles S, Gosley S, Fulbrook P, Sketcher-Baker K, Cook JL (2017). Pressure injury prevalence in intensive care versus non-intensive care patients: a state-wide comparison. Aust Crit Care.

[7] Solis LR, Hallihan DP, Uwiera RR, Thompson RB, Pehowich ED, Mushahwar VK (2007). Prevention of pressure-induced deep tissue injury using intermittent electrical stimulation. J Appl Physiol (1985).

[8] Gyawali S, Solis L, Chong SL, Curtis C, Seres P, Kornelsen I, Mushahwar VK (2011). Intermittent electrical stimulation redistributes pressure and promotes tissue oxygenation in loaded muscles of individuals with spinal cord injury. J Appl Physiol (1985).

[9] Solis L, Gyawali S, Curtis CA, Chong S, Thompson R, Mushahwar V (2008). Changes in superficial pressure and tissue oxygenation levels due to contractions elicited by intermittent electrical stimulation for the prevention of deep tissue injury.

[10] Ahmetović A, Mushahwar VK, Sommer R, Schnepf D, Kawasaki L, Warwaruk-Rogers R, ... Chan KM. Safety and feasibility of intermittent electrical stimulation for the prevention of deep tissue injury. Adv Wound Care. 2015;4(3):192–201. 10.1089/wound.2014.0569. 10.1089/wound.2014.0569 PMC4352702 25785240

[11] Kane A, Warwaruk-Rogers R, Ho C, Chan M, Stein R, Mushahwar VK, Dukelow SP (2017). A feasibility study of intermittent electrical stimulation to prevent deep tissue injury in the intensive care unit. Adv Wound Care.

[12] Chan A-W, Tetzlaff JM, Gotzsche PC, Altman DG, Mann H, Berlin J, Dickersin K, Hrobjartsson A, Schulz KF, Parulekar WR, Krleza-Jeric K, Laupacis A, Moher D (2013). SPIRIT 2013 explanation and elaboration: guidance for protocols of clinical trials. BMJ.

[13] Zhang Z (2016). Multiple imputation with multivariate imputation by chained equation (MICE) package. Ann Transl Med.

[14] Gebski V, Byth K, Asher R (2021). Recurrent time-to-event models with ordinal outcomes. Pharm Stat.

[15] Rutterford C, Copas A, Eldridge S (2015). Methods for sample size determination in cluster randomized trials. Int J Epidemiol.

[16] Ahtiala MH, Kivimäki R, Laitio R, Soppi ET. The Association Between Pressure Ulcer/Injury Development and Short-term Mortality in Critically Ill Patients: A Retrospective Cohort Study. Wound Manag Prev. 2020;66(2):14–21. 10.25270/wmp.2020.2.1421. 10.25270/wmp.2020.2.1421 32294060

