# Supplementary material for: Pressure injury treatment by intermittent electrical stimulation (PROTECT-2): protocol for a multicenter randomized clinical trial
Source: Trials. 2024 May 10;25:313. doi: 10.1186/s13063-024-08085-x (PMC11083768; doi:10.1186/s13063-024-08085-x)
Supplement: Supplementary file 2 — Additional file 2.Case report form (CRF). [file 13063_2024_8085_MOESM2_ESM.docx]

**Case Report Form**

**The PROTECT 2 ICU Study: Pressure Injury Treatment by Intermittent Electrical Stimulation: A Randomized, Controlled Trial**

| Study ID: ☐☐☐-☐☐-☐☐☐☐ | Subject Initials: ☐☐☐ |
| --- | --- |

**A.1 Eligibility**

| **A.1.1. Inclusion Criteria** | Yes / No |
| --- | --- |
| 1. Either new or established stage 1 or 2 sacral or ischial pressure ulcer in the ICU environment. |   |
| 1. Participants capable of giving informed consent, or if appropriate, participants having an acceptable individual capable of giving consent on the participant’s behalf. |   |

| **A.1.2. Exclusion Criteria** | Yes / No |
| --- | --- |
| 1. BMI > 40 |   |
| 1. Unstable spinal, pelvic, or hip fractures that may be displaced by a forced contraction |   |
| 1. Rhabdomyolysis |   |
| 1. Presence of permanent pacemaker or AICD, and for those with external wires after cardiac surgery, those who are using or at high risk for the development of a requirement for an external pacemaker |   |
| 1. Skin breakdown or malignant skin involvement over the gluteal regions that would preclude the use of surface electrodes |   |

**A.2. Patient baseline characteristics**

**A.2.1. Demographic**

 Male  Female Height (cm): _____ Weight (kg): _____ BMI: _____ (kg/m2): Age _(yrs.)_: _____ ASA (II-IV): ____Ethnicity (race): Caucasian  Black/African American Latino other:______

| **A.2.2. Critical Care Diagnosis** | |
| --- | --- |
| ICD-10 Code | Diagnosis |
| ________ | ____________________________________________________ |
| ________ | ____________________________________________________ |
| ________ | ____________________________________________________ |
| ________ | ____________________________________________________ |
| ________ | ____________________________________________________ |
| ________ | ____________________________________________________ |

**A.2.3. Severity of the illness on ICU day 1**

APACHE IV score: _______ SOFA score: _______

| **A.2.4. Preconditioning factors** |
| --- |
| **Serum Albumin during the last 14 days?** Yes No |
| **Serum Albumin :** ______ g/dL  N/A |
| **History of type II diabetes mellitus?** Yes No **Hemoglobin A1C level:** ______ |
| **Latest Hemoglobin level:** ______ g/dL |
| **Cumulative fluid balance at enrollment:** Positive Negative Amount: _______ cc |
| **Current steroid use > 3 days:** Yes No Cumulative Hospital Prednisone equivalents: _______ mg |
| **Active COVID-19 infection for current hospitalization:** Yes No Unknown |
| **Organ transplant recipient for the current hospital stay?** Yes No |
| **Hospital length of stay prior to enrollment:** _______ days |
| **Braden Score at Enrollment ________** |

| **A.2.5. Hemodynamic and Ventilatory support at enrollment** | |
| --- | --- |
| Medication | Dosage |
| Norepinephrine: Yes No | ______ mcg/min |
| Vasopressin: Yes No | ______ unit/min |
| Phenylephrine: Yes No | ______ mcg/min |
| Dopamine: Yes No | ______ mcg/kg/min |
| Epinephrine: Yes No | ______ mcg/min |
| Nitroglycerine: Yes No | ______ mcg/kg/min |
| Dobutamine: Yes No | ______ mcg/kg/min |
| Milrinone: Yes No | ______ mcg/kg/min |
| Midodrine: Yes No | ______mg |

***A.2.6.* Life Support Device**

 Mechanical ventilator  Non-invasive ventilator  Renal Replacement Therapy  Ventricular Assist Device  ECMO

**B. Randomization**

| **Patient was randomized?** Yes No **Date:** ___/___/______ **Time:** ____:____ |
| --- |
| **Ulcer occurrence:** New Previusly stablished |
| **Stratification by pressure injury status:**  Stage I Injury  Stage II Injury |
| **Study Arm:**  IES Device + Standard of Care  Standard of Care |
| **IES device application: Date:** ___/___/______ **Time:** ____:____ |

**C. Data collection during ICU hospitalization**

**C.1. Day 1 – Wound Examination**

| **C.1.1. Ulcer characteristics** |
| --- |
| - **Was the pressure ulcer evaluated for this assessment? (Note: Patient can be off the IES and still be assessed)** Yes No |
| **If not:**  Patient' or  LAR refused the pressure ulcer assessment (explain) |
|  Clinical decision to not assess the ulcer (explain, e.g. patient too unstable to be moved) |
|  Shift did not assess or record |
|  The patient died |
|  Other (comment) |
| **If Yes: Pressure Injury Stage:** 1 2 3 4 U sDTI Healed Missing (explain) |
| - **The Ulcer is:** the same  resolved  worse Missing (explain) |
| - **Was the patient using the IES system?** Yes No |
| **If Not: Why?** Explain [dropdown] 1) patient is in control group 2) other [add comment]____________ |
| **If Yes: Any Electrode induced skin toxicity?**  Yes No  N/A Missing (explain) |
| - **Any IES system dysfunctionality, including lack of visible muscle contraction upon testing?** . Yes No  N/A Missing (explain) |

| **The patient stayed one more day?** Yes No  N/A  If yes a C.2. question box will appear |
| --- |

**C.2. Day 2 – Wound Examination**

| **C.2.1. Ulcer characteristics** |
| --- |
| - **Was the pressure ulcer evaluated for this assessment? (Note: Patient can be off the IES and still be assessed)** Yes No |
| **If not:**  Patient' or  LAR refused the pressure ulcer assessment (explain) |
|  Clinical decision to not assess the ulcer (explain, e.g. patient too unstable to be moved) |
|  Shift did not assess or record |
|  The patient died |
|  Other (comment) |
| **If Yes: Pressure Injury Stage:** 1 2 3 4 U sDTI Healed Missing (explain) |
| - **The Ulcer is:** the same  resolved  worse Missing (explain) |
| - **Was the patient using the IES system?** Yes No |
| **If Not: Why?** Explain [dropdown] 1) patient is in control group 2) other [add comment]____________ |
| **If Yes: Any Electrode induced skin toxicity?**  Yes No  N/A Missing (explain) |
| - **Any IES system dysfunctionality, including lack of visible muscle contraction upon testing?** . Yes No  N/A Missing (explain) |

| **Enrollment in:** Non-ICU patient  ICU patient |
| --- |

| **Patient and Assessor Questionairre Side Effects for PROTECT2 Study Device** |
| --- |
| **Patient**  **Have you experienced any of the following events since the last visit by the study assessor?** |
| • Difficulty falling asleep or staying asleep  N/A / No / Yes - Severity grade: 1 2 3 4 5 6 7 8 9 10(worst |
| • Distraction or discomfort due to stimulation  N/A / No / Yes - Severity grade: 1 2 3 4 5 6 7 8 9 10(worst) |
| • Feeling of electrical shock?  N/A / No / Yes - Severity grade: 1 2 3 4 5 6 7 8 9 10(worst)  Where: _______________________________ |
| **Assessor**  **Have you noticed any of the following signs in the patient’s skin underneath or around the pads since the last visit?** |
| • Redness  N/A / No / Yes - Severity grade: 1 2 3 4 5 6 7 8 9 10(worst |
| • Skin irritation, blistering, or swelling  N/A / No / Yes - Severity grade: 1 2 3 4 5 6 7 8 9 10(worst) |

| **The patient stayed one more day?** Yes No  N/A  If yes a C.3. question box will appear |
| --- |

**D. Last Hospitalization Day**

| **D.1. Discharge** |
| --- |
| **Date:** ___/___/______ **Time:** ____:____ |
| **ICU length of stay:** ____ Days **ICU readmissions?:** Yes No  **Hospital length of stay:** ____ Days from enrollment to hospital discharge or death **Discharge Destination (**Death, Hospice, Acute Rehab, Long Term Acute Care (LTAC), Skilled Nursing Facility (SNF), home)**:** _____ |
|  |

| **D.2. Ulcer characteristics** |
| --- |
| \| **C.1.1. Ulcer characteristics** \| \| --- \| \| **If Yes: Was the Pressure Ulcer Evaluated?** Yes No \| \| **If Not:**  Clinical decision not to assess the ulcer (explain, e.g. patient so unstable to be moved) \| \|  Patient refused to be assessed (explain) \| \|  Pressure ulcer was not recorded by this shift (explain) \| \|  Other (explain) \| \| **If Yes: Pressure Injury Stage:** 1 2 3 4 U sDTI Healed Missing (explain) \| \| **The Ulcer is:** **the same**  **resolved**   **worse** Missing (explain) \| \| **Was the patient using the IES system?** Yes No \| \| **If Not: Why?** Explain [dropdown] 1) patient is in control group 2) other [add comment]____________ \| \| **If Yes: Any Electrode induced skin toxicity?:** Yes No  N/A Missing (explain) \| |
| **Number of OR trips for which the device was turned off:** ____ |
| **Cumulative number of hours for which the patient was in the OR with the device turned off:**____ |
| **Maximum number of hours in the OR when the device was turned off:**____ |
| **Other Treatments related to the ulcer** :  Grafting procedures  Medications  Hyperbarics  N/A |

| **D.3. Vasoactive Drugs** | | |
| --- | --- | --- |
| Medication | Cumulative dose (mcg) | Daily maximum dose |
| Norepinephrine (mcg/min) |  | Day 1: **______** Day2: **______** Day3: **______** Day 4: **______** Day5: **______** Day6+: **_____** |
| Phenylephrine (mcg/min) |  | Day 1: **______** Day2: **______** Day3: **______** Day 4: **______** Day5: **______** Day6+: **_____** |
| Vasopressin (unit/min) |  | Day 1: **______** Day2: **______** Day3: **______** Day 4: **______** Day5: **______** Day6+: **_____** |
| Dopamine (mcg/kg/min) |  | Day 1: **______** Day2: **______** Day3: **______** Day 4: **______** Day5: **______** Day6+: **_____** |
| Epinephrine (mcg/min) |  | Day 1: **______** Day2: **______** Day3: **______** Day 4: **______** Day5: **______** Day6+: **_____** |
| Nitroglycerine (mcg/kg/min) |  | Day 1: **______** Day2: **______** Day3: **______** Day 4: **______** Day5: **______** Day6+: **_____** |
| Dobutamine (mcg/kg/min) |  | Day 1: **______** Day2: **______** Day3: **______** Day 4: **______** Day5: **______** Day6+: **_____** |
| Milrinone (mcg/kg/min) |  | Day 1: **______** Day2: **______** Day3: **______** Day 4: **______** Day5: **______** Day6+: **_____** |
| Midodrine (mg) |  | Day 1: **______** Day2: **______** Day3: **______** Day 4: **______** Day5: **______** Day6+: **_____** |

***D.4. Life Support Device***

 Mechanical ventilator  Non-invasive ventilator  Renal Replacement Therapy  Ventricular Assist Device  ECMO

**D.5. *Ventilatory Support***

Mechanical ventilator Yes No Number of Days: ______

Non-invasive ventilator (excluding routine CPAP) Yes No Number of Days: ______

| **D.6. Labs, medications, and hemodynamics since enrollment** |
| --- |
| Average Hemoglobin level: ______ g/dL |
| Minimum Hemoglobin level: ______ g/dL |
| Number of units of packed red blood cells transfused: ______ |
| Number of hypotensive episodes (MAP <65): ______ |
| Average blood oxygen saturation: ______% |
| Cumulative systemic steroid dose since admission _______ prednisone equivalents |
| Neuromuscular blockade used during study period Yes No |
| Mean blood glucose: ______ mg/dL |

**D.7. Organ transplant recipient for the current hospital stay?** Yes No

**E. One month follow-up**

| **Patient is:** Alive Dead Cause of Death:________ |
| --- |
| **Number of wound care follow up visits for sacral or ischial pressure injury:** ______ |
| **Wound care device or medical equipment related to sacral or ischial pressure injury:** Yes No |

**F. Three months follow-up**

| **Patient is:** Alive Dead Cause of Death:________ |
| --- |
| **Number of wound care follow up visits for sacral or ischial pressure injury:** ______ |
| **Wound care device or medical equipment related to sacral or ischial pressure injury:** Yes No |
