# Supplementary material for: Pressure injury treatment by intermittent electrical stimulation (PROTECT-2): protocol for a multicenter randomized clinical trial
Source: Trials. 2024 May 10;25:313. doi: 10.1186/s13063-024-08085-x (PMC11083768; doi:10.1186/s13063-024-08085-x)
Supplement: Supplementary file 3 — Additional file 3. Patient and Assessor Questionnaire. Questions for patients based upon their experience as well as the assessor based on their patient evaluation. [file 13063_2024_8085_MOESM3_ESM.docx]

## **Appendix E** Patient and Assessor Questionnaire

**Side Effects for PROTECT2 Study Device**

*Patient*

Have you experienced any of the following events since the last visit by the study assessor?

• Difficulty falling asleep or staying asleep

N/A / No / Yes - Severity grade: 1 2 3 4 5 6 7 8 9 10(worst)

• Distraction or discomfort due to stimulation

N/A / No / Yes - Severity grade: 1 2 3 4 5 6 7 8 9 10(worst)

• Feeling of electrical shock?

N/A / No / Yes - Severity grade: 1 2 3 4 5 6 7 8 9 10(worst)

Where: _______________________________

*Assessor*

Have you noticed any of the following signs in the patient’s skin underneath or around the pads since the last visit?

• Redness

N/A / No / Yes - Severity grade: 1 2 3 4 5 6 7 8 9 10(worst)

• Skin irritation, blistering, or swelling

N/A / No / Yes - Severity grade: 1 2 3 4 5 6 7 8 9 10(worst)
