# Supplementary material for: Pressure injury treatment by intermittent electrical stimulation (PROTECT-2): protocol for a multicenter randomized clinical trial
Source: Trials. 2024 May 10;25:313. doi: 10.1186/s13063-024-08085-x (PMC11083768; doi:10.1186/s13063-024-08085-x)
Supplement: Supplementary file 4 — Additional file 4. Baseline factors for treatement heterogeneity. [file 13063_2024_8085_MOESM4_ESM.docx]

Treatment Effect Heterogeneity Comparisons

- Demographic data, sex, age
- Critical care diagnosis
- Severity of illness screen with SOFA and APACHE IV score on ICU day 1 and at enrollment
- Body height (cm) and weight (kg) reported as BMI
- Serum albumin measured at enrollment or within 14 days prior to enrollment
- History of type II diabetes mellitus (yes/no)
- Hemoglobin A1c level
- Cumulative fluid balance at enrollment
- Baseline pressure injury assessment
- Location of enrollment (ICU vs. non-ICU)
- Pressure injury development (new, established)
- Vasoactive drug (norepinephrine, phenylephrine, dopamine, epinephrine, milrinone, nitroglycerin, dopamine, dobutamine, midodrine) dosage reported in norepinephrine equivalents

Treatment Effect Outcomes at Discharge:

- - Pressure injury assessment
  - Electrode induced skin toxicity
  - Mechanical support device during hospital stay (ventricular assist device, ECMO, renal replacement therapy)
  - Organ transplant recipient for the current hospital stay
  - Cumulative and daily maximum vasopressor dose for the hospital stay while on the device
  - Number of ventilator days
  - ICU length of stay
  - Hospital length of stay from enrollment to hospital discharge or death
  - Reason for discontinuation
  - All inpatient treatments related to the ulcer (grafting procedures, medications, hyperbarics)
  - Number of units of packed red blood cells transfused while on the device
  - Number of operating room procedures for which device was turned off
  - Cumulative hours in the operating room for those procedures for which the device was turned off
- OR time with the device turned off >4 hours (y/n)
  - Average hemoglobin over the duration of the device
  - Minimum hemoglobin while on the device
  - Number of hypotensive episodes while on the device
  - Average blood oxygen saturation while on the device
  - Mean blood glucose while on the device
  - ICU readmission during the hospital stay after enrollment
  - Discharge destination

Additional comparisons and data for analysis:

1. Compare IES to standard of care for the primary outcome in the following specific patient populations:
2. Ventricular assist device
3. ECMO
4. Organ transplantation
5. Describe descriptive outcomes of populations
   1. COVID positive during hospital stay (y/n)
   2. Steroid use during study period (y/n)
   3. Hospital length of stay
   4. Ventilator days (0-7, 7-21, >21)
   5. Number of hypoxemia events while using device (SaO2<88)
   6. Vasopressors used and dosages for the duration of device usage (norepinephrine equivalents)
   7. ICU length of stay (<7 days, 7-21 days, >21 days
   8. Number of units of packed red blood cells transfused while on the device
   9. Number of operating room procedures for which device was turned off (0, 1-2, >3)
   10. Cumulative hours in the operating room for those procedures for which the device was turned off (0/n/a, 1-4, 4-8, >8)
   11. OR time with the device turned off >4 hours (y/n)
   12. Average hemoglobin over the duration of the device (<7, 7-8, 8-10, >10)
   13. Minimum hemoglobin while on the device
   14. Average blood oxygen saturation while on the device (<92, 92-95, 95-100)
   15. Number of hypotensive episodes while on the device (MAP <65)
   16. Mean blood glucose while on the device
6. Description and frequency of adverse events related to IES, including patient and assessor perception
7. 0 point Likert Scale for “Difficulty Following Asleep” as answered by the patient
8. 10 point Likert Scale for “Distraction or Discomfort due to Stimulation” as answered by the patient
9. 10 Point Likert Scale for “Feeling of Electric Shock as answered by the patient
10. 10 Point Likert Scale for “Redness” since last assessment as answered by the assessor
11. 10 point Likert Scale for “Skin Irritation, Blistering, or Swelling” since last assessment as answered by the assessor
12. Cost effectiveness analysis study comparing traditional care to IES
