# Supplementary material for: Pressure injury treatment by intermittent electrical stimulation (PROTECT-2): protocol for a multicenter randomized clinical trial
Source: Trials. 2024 May 10;25:313. doi: 10.1186/s13063-024-08085-x (PMC11083768; doi:10.1186/s13063-024-08085-x)
Supplement: Supplementary file 5 — Additional file 5. Date and safety monitoring board (DSMB). A list of all members of the DSMB. [file 13063_2024_8085_MOESM5_ESM.docx]

Data and Safety Monitoring Board (DSMB)

Chair: Mark Zahniser, MD; CCF

Daniel Sessler, MD; CCF

Brett Wakefield, MD; CCF

Balaram Anandamurthy, MD; CCF

Edward Mascha, PhD; CCF

Sean Dobson, MD; Wake Forest University
