# Supplementary material for: Pressure injury treatment by intermittent electrical stimulation (PROTECT-2): protocol for a multicenter randomized clinical trial
Source: Trials. 2024 May 10;25:313. doi: 10.1186/s13063-024-08085-x (PMC11083768; doi:10.1186/s13063-024-08085-x)
Supplement: Supplementary file 7 — Additional file 7. Funding documentation. [file 13063_2024_8085_MOESM7_ESM.pdf]

Cleveland Clinic Foundation

Notice of Award Acceptance Form

Awarded Date: 12/14/2021 Institution: CCF Other:

Award Type: Contract

Project Type: Clinical Research Other:

Proposal Type: New Other:

PI Name: Donaldson, Chase

Administrative Home/Dept Name: Anethesia Department No. 070

Title: PROTECT 2

Sponsor: REHABTRONICS, INC Sponsor Award #: RHTX2112CD

Originator Of Funds: Originator of Funds Award #:

Current Period Funding		
Budget Start Date:		9/28/2021
Budget End Date:		9/27/2031
Direct Costs:		$688,000.00
Indirect Costs:		$206,400.00
FAC Rate:	30.00 %	
**Total Costs:**		$894,400.00

Cumulative Funding		
Project Start Date:		9/28/2021
Project End Date:		9/27/2031
Direct Costs:		$688,000.00
Indirect Costs:		$206,400.00
FAC Rate:	30.00 %	
**Total Costs:**		$894,400.00

GPID: 24777 Project Number: SP005011

Terms and Conditions

T&C Code	Description

Award Specific Terms and Conditions

Please see the contract for full terms and conditions.
The budget is based on 300 patients at $2,743 per patient plus administrative costs.

Key Personnel

PI Name	Eft %	Eft Cal Mon	CS %
Chase Donaldson	0.00	0.00	

Comments

GAID 11214 SA00005474 CRS00010418

Compliance

Compliance	Status	Reference	Approval Date	Expiration Date
HS - Human Subjects	Approved	21-1009	12/3/2021	11/18/2022

Institutional Official Signature/Date: Jacqueline Whatley 12/15/2021

OSRP-R Signature/Date: Carla Hannan 12/15/2021

Cleveland Clinic - Law Department

This page needs to be retained with the Agreement at all times.

COMPANY INFORMATION

REHABTRONICS, INC
#4352, 10230 - JASPER AVENUE
EDMONTON, ALBERTA T5J 4P6

CONTRACT INFORMATION

Contract ID: 4251048
Master Agreement Number:
Dept Reference No.: 24777
Contract Description: INVESTIGATOR-SPONSORED RESEARCH AGREEMENT, PROTECT 2 STUDY OF PRELIVIA SYSTEM; PI CHASE DONALDSON
Institute: Anesthesiology
Submitting Dept: OTHER
Contract Amount:

Principal Investigator: CHASE DONALDSON

Dept Contact: MARK METTLER

TERM INFORMATION

Effective Date:
Expiration Date:
Term Type: Fixed

LEGAL TEAM INFORMATION

Attorney: AMBER DOWD

Paralegal:

Contract approved as to form for: 4251048
Attorney: DOWD, AMBER
By: Zimmer, Kathy
Date: 9/9/2021 9:09:07 AM

INVESTIGATOR-SPONSORED RESEARCH AGREEMENT

This INVESTIGATOR-SPONSORED RESEARCH AGREEMENT (this "**Agreement**") is made effective as of the date signed by the last party ("**Effective Date**") by and among

Rehabtronics Inc., ("**GRANTOR**"),
#4350, 10230 Jasper Avenue
Edmonton, Alberta, Canada T5J 4P6

and **THE CLEVELAND CLINIC FOUNDATION ("INSTITUTION")**,an Ohio non-profit corporation having a place of business at 9500 Euclid Avenue, Cleveland, OH 44195. Hereinafter, the Grantor and Institution may be referred to as "Party" or "Parties".

PRELIMINARY STATEMENTS

A. INSTITUTION desires to conduct (as defined in Section 1.2(a)), and Grantor desires to support the conduct of, an investigator-sponsored research study under the terms of this Agreement (the "**Study**"), under the direction of the investigator Dr. Chase Donaldson (the "**INVESTIGATOR**").

B. The Study will relate to the Prelivia System™ ("**Study Device**") and may involve other medicinal products as required by the Protocol (as defined in Section 1.1).

C. The Parties desire to agree on various terms and conditions to govern the Parties' negotiation and performance of the Study.

The Parties hereby agree as follows:

ARTICLE 1. STUDY GOVERNANCE

1.1 IDENTIFICATION OF STUDY

The Study title is "The PROTECT2 Study: Pressure injury prevention by intermittent Electrical sTimulation 2" attached hereto as Attachment B the "**Protocol**"), which is, together with any amendments made thereto, which shall be considered to constitute an integral part of this Agreement.

1.2 CONDUCT OF STUDY

(a) INSTITUTION shall and shall cause INVESTIGATOR to conduct and supervise the Study at INSTITUTION's facilities.

(b) INSTITUTION shall cause INVESTIGATOR and all Study Personnel (as defined in Section 1.2(d)) to conduct the Study in accordance with (i) the Protocol, and(ii) this Agreement. Both Parties specifically intend to comply with all laws, rules, regulations, good clinical practices to the extent applicable to either Party including, but not limited to International Conference on Harmonization / Good Clinical Practice guidelines, as adopted or issued by Health Canada ("**ICH-GCP**") and the requirements and official guidance of relevant health authorities, including, but not limited to, (A) Health Canada regulations, polices and guidelines, (B) applicable laws regarding the protection of privacy, personal data and medical data, including the *Health Information Act* (Alberta) ("**HIA**"), (C) applicable laws and regulations regarding the reporting of any fees and other expenditures paid to healthcare professionals; (D) all applicable anti-bribery legislation, (F) TCPS 2 - Tri-Council Policy Statement: Ethical Conduct for Research Involving Humans (collectively for clause (iii), as the same may be amended from time to time, and (G) the federal anti-kickback

statute (42 U.S.C. §1320a-7b) and the related safe harbor regulations; (ii) the Limitation on Certain Physician Referrals, also referred to as the "Stark Law" (42 U.S.C. §1395nn); (iii) The Federal Food, Drug, and Cosmetic Act (21 U.S.C. §§ 301 et seq.); (iv) the Public Health Service Act (42 U.S.C. § 201 et seq.); (v) the Health Insurance Portability and Accountability Act of 1996 and the Health Information Technology for Economic and Clinical Health Act (collectively, "HIPAA"); (vi) all applicable anti-corruption laws, including the United States Foreign Corrupt Practices Act of 1977 and the UK Bribery Act of 2010, as each may be amended, or any rules and regulations thereunder; (vii) any and all applicable U.S. export control laws and regulations, as well any and all embargoes and/or other restrictions imposed by the Treasury Department's Office of Foreign Asset Controls; (viii) the disclosure of certain payments and other transfers of value to health care professionals and institutions, including Section 1128G of the Social Security Act, 42 U.S.C. 1320a-7h (the "Sunshine Act"); and (ix) all comparable state and local laws and regulations relating to the conduct of the Study ("Applicable Law").

(c) INSTITUTION shall ensure that an appropriate Research Ethics Board ("IRB") reviews, approves, and oversees the Study as may be required by Applicable Law and/or the Protocol.

(d) All employees, staff and agents of INSTITUTION and all other persons providing services in the conduct of that Study (all such persons, including any SUBINVESTIGATORS, as defined in Section 1.3(b), collectively, "**Study Personnel**") will perform the Study on behalf of INSTITUTION. For the avoidance of doubt, Study Personnel shall not include any employee, agent, or other person working on behalf of GRANTOR.

1.3 INVESTIGATOR; SUBINVESTIGATORS; REPLACEMENT INVESTIGATOR

(a) INSTITUTION shall ensure that INVESTIGATOR personally supervises the conduct of the Study including by ensuring that all Study Personnel are qualified by experience or training to perform the activities assigned to such individual and supervising the performance of such activities.

(b) The INSTITUTION may appoint other individuals employed by INSTITUTION as subinvestigators to assist in the conduct of the Study (each, a "**SUBINVESTIGATOR**").

(c) If the INVESTIGATOR becomes unable to conduct the Study, INSTITUTION shall propose to GRANTOR the appointment of a new INVESTIGATOR, which proposal shall provide at least a curriculum vitae. In the event GRANTOR consents to the proposed new INVESTIGATOR, the Parties shall work in good faith to amend this Agreement and any other documents as necessary to reflect such substitute to ensure compliance with all Applicable Law.

1.4 STUDY PERSONNEL

INSTITUTION shall ensure that INVESTIGATOR takes all reasonable steps to inform Study Personnel and SUBINVESTIGATORS of all of their obligations under this Agreement, and shall require Study Personnel and SUBINVESTIGATORS to fully comply with same. INSTITUTION shall be liable for any failure by Study Personnel to perform obligations under this Agreement. Such obligations include, but are not limited to, obligations with respect to confidentiality, publication, intellectual property, and use and disclosure of Study Data.

1.5 NATURE OF CONSIDERATION

The Parties acknowledge and agree that no part of any funding provided under this Agreement is a prohibited payment for recommending or arranging for the referral of business or the ordering of items or

services. Additionally, the Parties agree that neither this Agreement nor any consideration paid hereunder is contingent upon INSTITUTION'S or INVESTIGATOR's use or purchase of any GRANTOR products.

The Parties acknowledge that any financial support provided under this Agreement constitutes Fair Market Value for Institution's performance of the Study and represents the real cost to Institution in performing the activities required to initiate and conduct the Study. The Parties further acknowledge that this Agreement, and any consideration paid under it, is not contingent upon Institution's purchase or use of Grantor's products.

ARTICLE 2. OBLIGATIONS OF INSTITUTION, INVESTIGATOR AND STUDY PERSONNEL

2.1 PERFORMANCE OF STUDY

INSTITUTION shall ensure that INVESTIGATOR performs the Study in an ethical and professional manner and shall use his/her reasonable efforts to complete the Study within the time period set forth in the Protocol. In furtherance of the Study, the INSTITUTION will, in addition to all other requirements of the Protocol or Applicable Law and to the extent applicable:

(a) prior to initiating the Study, submit to GRANTOR necessary IRB approvals and any other forms, reports, or documents required by external regulatory bodies, including informed consent forms ("**Regulatory Documents**"), to the extent required by Applicable Law;

(b) respond promptly to telephone, fax, or e-mail contacts from GRANTOR regarding Study status;

(c) register and post results of the Study on a publically available bank such as www.clinicaltrials.gov, as required by Applicable Law;

(d) maintain accountability and responsibility for all Study-related funds and Study Devices supplied by GRANTOR and used in the Study. GRANTOR shall have the right, upon advance written notice, to access all records concerning Study-related funds, Study Device and Regulatory Documents, at mutually agreeable times during normal business hours, and to make copies of those records at reasonable cost. This right of access will survive completion or termination of the Study;

(e) provide copies to GRANTOR of all amendments to the Protocol and consent forms, as finally approved by the IRB, within five (5) business days after such approval;

(f) notify GRANTOR in the event of an inspection by regulatory authorities and the results of any such inspection;

(g) report to GRANTOR immediately if any regulatory authority places a hold or any restriction on any aspect of the Study;

(h) as reasonably requested by GRANTOR, will provide any additional information required to fulfill GRANTOR's regulatory reporting requirements, including, but not limited to, those related to product quality; and

(i) within one (1) year of the conclusion or termination of the Study, submit to GRANTOR, as required, a final Study report regarding the Study (the "**Study Report**"), which will contain relevant information about the execution of the Study including, but not limited to:

(i) Subject demographics;

(ii) Methodology;

(iii) A summary of the Study Data (as hereinafter defined) generated in the course of the Study;

(iv) Safety data, if applicable, including listings of all non-serious and serious adverse events, pregnancies, discontinuations associated with an adverse event, and deaths, as well as a brief narrative description for each serious adverse event and death; and

(v) Discussion and conclusions.

(j) INSTITUTION shall ensure that INVESTIGATOR complies with all Institution policies, procedures and guidelines applicable to the conduct of the Study..

2.2 DEBARMENT

INSTITUTION represents and warrants that neither it nor INVESTIGATOR is currently using, and shall not knowingly use any person or entity debarred in any capacity in connection with performing any Study which GRANTOR has indicated the results of which are intended to be included in applications to the FDA and/or any other regulatory authorities worldwide. INSTITUTION acknowledges that this representation, certification, and agreement will be relied upon by GRANTOR when making such applications. INSTITUTION shall promptly notify GRANTOR of any breach of the foregoing representation, certification or agreement. This provision survives the termination or expiration of this Agreement.

2.3 ADVERSE EVENT AND SAFETY REPORTING

(a) INSTITUTION shall ensure that all Study Personnel report to GRANTOR all Serious Adverse Events and other reportable events in the course of the Study in accordance with Applicable Law and the Protocol.

ARTICLE 3. **SUPPORT**

3.1 PAYMENT AND SUPPORT

(a) Study Budget. GRANTOR or its designee shall pay to INSTITUTION or other entity specifically referred to in Attachment A – Study Budget hereto (such payee, the "**Payee**") such amount as determined and in the manner set forth in Attachment A. INSTITUTION agrees that all such payments made by GRANTOR or its designee reflect all research support received under this Agreement and reflect no more than local fair market value for the research supported under this Agreement.

Payments made under the terms of this Agreement will be made payable to:

The Cleveland Clinic Foundation
PO Box 931531-Corporate
Cleveland, OH 44193-5012

3.2 BUDGET

INSTITUTION acknowledges that GRANTOR shall provide only the funding and/or support for the Study to the INSTITUTION as expressly set forth in Attachment A. Without limiting the generality of the foregoing, except to the extent expressly set forth in such Attachment A, GRANTOR will not be responsible

for arranging or paying for any laboratory or other facilities, equipment and supplies required for performance of the Study.

3.3 PROHIBITION ON DOUBLE-BILLING

INSTITUTION shall not seek, collect, or assist the Study subject in seeking or collecting, reimbursement from any health insurance plan, or governmental medical plan or other government-provided health coverage available to the Study subject for any medical expenses (including expenses relating to Study procedures or Study complications) covered by the research support provided by GRANTOR under this Agreement.

3.4 PROVISION OF STUDY DEVICE

Where GRANTOR is providing Study Devices for a Study, GRANTOR shall provide to INSTITUTION, without cost, that amount of Study Devices and electrodes required to conduct the Protocol. INSTITUTION shall use and return, and cause all Study Personnel and SUBINVESTIGATORS to use and return, all Study Devices supplied by GRANTOR only as specified in the Protocol for the Study. INSTITUTION shall return all Study Devices supplied by GRANTOR at the conclusion or early termination of the Study. GRANTOR represents and warrants that the Study Device has been manufactured in accordance with Good Manufacturing Practices and in full compliance with all applicable government and regulatory requirements and specifications, including without limitation the requirements of the Medical Devices Branch of Health Canada, AND(i) it has obtained all necessary governmental and regulatory approvals, waivers or exemptions needed to conduct the Study and provide the Product including without limitation, all applicable FDA approvals; and that all approvals will be in full force and effect during the Study; (ii) it has disclosed to Institution and applicable government authorities all relevant, material information concerning the safety, use, and efficacy of the Study Devices; (iv) it is the owner of the Study Devices or has the authority to grant all of the rights related to the Study Devices provided for in this Agreement, including the right to authorize Institution to use the Study Devices in the manner described in the Protocol; and (v) to the best of its actual knowledge, after reasonable investigation, the use of the Study Devices for Study purposes will not infringe any rights, patent or otherwise, of any third party.

3.5 TRANSPARENCY

GRANTOR may disclose as required by Applicable Laws, the terms of this Agreement, including, without limitation, the total research support provided pursuant to this Agreement and details with respect thereto. When making such disclosures, GRANTOR reserves the right to attribute research support provided under this Agreement to each Payee, the INSTITUTION or person supported under this Agreement. INSTITUTION will provide reasonable assistance with GRANTOR' compliance with Applicable Laws and its policies relating to the reporting of payments and transfers of other things of value from drug manufacturers to healthcare providers.

ARTICLE 4. **CONFIDENTIAL INFORMATION**

4.1 DEFINITIONS

(a) "**Affiliate(s)**" means, with respect to a Party, a business entity that Controls, is under the Control of or under common Control with such Party.

(b) "**Control**" means the possession, directly or indirectly, of the power to direct or cause the direction of the management of the business entity, whether through ownership of voting securities or otherwise.

(c) "**Disclosing Party**" means, with respect to Confidential Information disclosed or made available under this Agreement, the Party who or whose Affiliate owns or otherwise controls that Confidential Information.

(d) "**Receiving Party**" means, with respect to Confidential Information disclosed or made available under this Agreement, the Party who receives or is otherwise exposed to such Confidential Information under this Agreement.

(e) "**Confidential Information**" means this Agreement and any information of a confidential or proprietary nature relating to the Study that is directly or indirectly disclosed or otherwise made available under this Agreement to the Receiving Party, whether written, graphic, oral, visual, tangible or intangible, in any form or format (including machine or computer readable code), provided that tangible disclosures are, wherever practicable, marked confidential and intangible disclosures are reduced to writing within thirty (30) days of such disclosure. Notwithstanding the foregoing, any information which is not marked or later confirmed in writing as "Confidential" shall be deemed Confidential Information if a reasonable person knowledgeable in the fields of clinical research or medical practice, would consider the information to be of a confidential or proprietary nature. Confidential Information does not include information that: (i) is now in the public domain or subsequently enters the public domain through no breach of this Agreement by the Receiving Party or its Affiliates; (ii) the Receiving Party receives from any third party who, to the knowledge of the Receiving Party is under no obligation to the Disclosing Party to keep such information confidential, without restriction as to use or confidentiality as shown by written or other tangible evidence; (iii) was in the Receiving Party's or its Affiliates' possession on a non-confidential basis prior to the time of disclosure by the Disclosing Party; (iv) is approved for release by the Disclosing Party or (v) is independently developed by or for the Receiving Party or its Affiliates by persons without access to the Confidential Information.

4.2 USE OF CONFIDENTIAL INFORMATION

The Receiving Party shall use Confidential Information solely in connection with the conduct of the Study for which it was provided or otherwise in the performance of this Agreement under which it was provided ("**Permitted Purpose**"). For avoidance of doubt, a Permitted Purpose may include publication but only when accomplished in accordance with Article 7 (Publication).

4.3 PERMITTED DISCLOSURES OF CONFIDENTIAL INFORMATION

The Receiving Party agrees to disclose Confidential Information only to persons that need to know such Confidential Information for a Permitted Purpose. Prior to disclosing Confidential Information to such persons, the Receiving Party shall advise said persons of the confidential nature of the Confidential Information and the relevant obligations contained in this Agreement. The Receiving Party shall be liable for unauthorized disclosure or use of Confidential Information by any person to whom it discloses such information. Notwithstanding anything in this Agreement to the contrary, INSTITUTION may disclose Confidential Information to the extent it is that is required to be disclosed to obtain ethical and administrative approval for the Study; that must be disclosed to potential Study subjects during the recruitment process or to subjects who are or were enrolled in the Study, or their lawful representatives, in order to obtain and maintain informed consent or as the information relates to their health, safety or diagnosis; or in the case of a multi-centre Study, is deemed necessary by the Institution in the interest of subject safety to be disclosed to IRBs of other Study sites.

4.4 REQUIRED DISCLOSURES

If the Receiving Party receives a subpoena or other administrative or judicial process demanding Confidential Information, the Receiving Party shall: (i) promptly inform the individual or entity issuing such subpoena or other government process of the existence of this Agreement; (ii) unless prohibited by law from doing so, promptly notify the Disclosing Party of the disclosure requirement (which will include a copy of any applicable subpoena or order); (iii) to the extent practicable, afford the Disclosing Party a reasonable opportunity to oppose, limit or secure confidential treatment for the required disclosure; and (iv) not oppose

any effort by the Disclosing Party to quash any such subpoena or other government process. If the Disclosing Party fails to intervene to quash said subpoena or other government process after being given notice and a reasonable opportunity to do so, or if such motion is denied by a court of competent jurisdiction, the Receiving Party shall disclose only that portion of the Confidential Information of the Disclosing Party that the Receiving Party is legally required to disclose. In the event that any Confidential Information is ordered to be produced in an action or proceeding, it will not automatically lose its confidential status through such use, and the Receiving Party shall take all reasonable and necessary steps to protect its confidentiality.

4.5 RETURN OF CONFIDENTIAL INFORMATION

Upon the termination or expiration of this Agreement, or at any other time upon the written request of the Disclosing Party, the Receiving Party shall promptly return to the Disclosing Party or at the Disclosing Party's request, destroy all Confidential Information in the Receiving Party's possession or control, together with all copies, summaries and analyses, regardless of the format in which the information exists or is stored. In case of destruction, the Receiving Party shall promptly send a written notice that destruction has been accomplished to the Disclosing Party. If the retention period for the Confidential Information is longer than seven (7) years, Disclosing Party will reimburse Receiving Party for additional reasonable storage costs plus management fees annually, upon receipt of invoice. However, the Receiving Party is entitled to retain one (1) copy of Confidential Information for the sole purposes of (a) determining its obligations under this Agreement; (b) as necessary to exercise publication rights under this Agreement; or (c) otherwise abiding by the record retention requirements of this Agreement or Applicable Law. Backups of electronic records containing Confidential Information shall not be required to be destroyed or returned but will be held in confidence until overwritten or erased in accordance with Receiving Party's standard practices associated therewith.

4.6 DURATION OF CONFIDENTIALITY

The obligations of confidentiality survive for five (5) years beyond expiration or earlier termination of this Agreement.

4.7 IRREPARABLE HARM

The Receiving Party acknowledges that breach of any of the obligations set forth in this Article 4 may cause the Disclosing Party irreparable harm, for which monetary damages may be an inadequate remedy. Therefore, in the event of breach or threatened breach of this Article 4, the Disclosing Party may entitled, in addition to any other remedy available at law or in equity, to seek injunctive relief or an order for specific performance from a court of competent jurisdiction.

4.8 PROHIBITION ON DISCLOSURES OR USE FOR FINANCIAL BENEFIT

Without limiting the generality of the foregoing, INSTITUTION shall not knowingly (i) disclose Confidential Information belonging to GRANTOR directly or indirectly to any financial, securities, or industry analyst, or to the media, except as authorized in writing by GRANTOR or (ii) use Confidential Information belonging to GRANTOR in connection with purchase or sale of any securities. Notwithstanding the foregoing, this Agreement shall not restrict in any way INSTITUTION from making investments in pooled investment vehicles such as mutual funds, or in individual securities in the ordinary course of business on behalf of the Institution's endowment, reserve fund or the like

ARTICLE 5. PATIENT PRIVACY

5.1 PATIENT HEALTH INFORMATION

The Parties shall comply, and shall require all of its respective personnel (including INVESTIGATOR) to comply, with: (a) all Applicable Law governing patient privacy and confidentiality of health information, including without limitation and to the extent applicable , HIA and its regulations and HIPAA ; (b) comply with any reasonable conditions and requirements set forth in HIPAA relating to the use, protection, disclosure, return or disposal of any Study subject's individually identifying health information ("Health Information") or relating to safeguards against the identification, direct or indirect, of any Study subject; (c) use Health Information only for the purpose of conducting the Study; (d) not publish the Health Information in a form that could reasonably enable the identity of any Study subject to be readily ascertained; (e) not make any attempt to contact a Study subject to obtain additional Health Information or identify a Study subject unless the Study subject has provided a Party with informed consent regarding the same; (f) be liable for the actions of its employees, agents, consultants or other persons for whom they are in law responsible respecting the collection, use or disclosure of Health Information and for ensuring compliance with this Article by these persons; and (g) ensure that if the identity of any Study subject is disclosed to it or its employees, agents, or consultants, such information shall not be disclosed to any third parties, except where such disclosure is required by law. The Parties shall each take all actions necessary to comply with such laws and regulations, including agreeing to amend this Agreement as necessary for compliance.

The Parties shall treat all Health Information disclosed as part of the data reporting of the Study or any monitoring or audit function as strictly confidential and in accordance with all applicable federal, state or local laws, and regulations governing confidentiality and privacy of individually identifiable health information, including HIPAA and HIA. If a Party’s use, transfer or processing of the data triggers other data privacy laws, the Party shall confirm that the authorization obtained is sufficient under such laws.

ARTICLE 6. INTELLECTUAL PROPERTY AND STUDY DATA

6.1 DEFINITIONS

(a) “**Background Invention**” means any Invention, improvements, discoveries, know-how, methods, operations, formulas, data, copyrights, ideas, software, developments and works of authorship i) owned or controlled by a Party prior to the Effective Date or ii) otherwise invented by or on behalf of a Party independently of this Agreement.

(b) “**Invention**” means any inventions, expressions of ideas, discoveries, devices, data, mechanisms, substances, works, trade secrets, know-how, formulae and methods, including improvements, whether or not protectable by patent, copyright or otherwise, and all intellectual property rights therein (including all related patents and patent applications).

(c) “**Medical Records**” means the medical records of Study subjects in connection with the Study.

(d) “**Study Data**” means all data, records and reports collected or created as a result of using GRANTOR’s Confidential Information and/or Study Drug. Study Data includes, without limitation, case report forms (“**CRFs**”) and all data reported on the CRF, any data summaries, any interim reports and any final report and all records regarding inventories and dispositions of Study Drug, and expressly excluded the Study Results. For the

4251048 - CCF LAW DEPARTMENT APPROVED AS TO FORM - ACD - 9/9/2021

avoidance of doubt, Institution is the exclusive owner of all Study Data , Medical Records, and any source documents.

(e) "**Study Invention**" means any Invention solely or jointly conceived, made, reduced to practice, or first demonstrated to have utility by GRANTOR, INSTITUTION, or any other Study Personnel in connection with this Agreement.

(f) **"Study Results"** means any results generated by conducting the Study. All right and title to Study Results will remain with the INSTITUTION.

6.2 INTELLECTUAL PROPERTY

(a) Intellectual property that any party hereto owned prior to execution of this Agreement, or develops independently of the Study and any other party's Confidential Information, is that party's separate property and is not affected by this Agreement. No party hereto has any claims to or rights in such intellectual property of the other parties hereto. **Furthermore, this Agreement is not a "work made for hire" agreement under the copyright laws of any country.**

(b) All information and results produced, generated or developed by the INVESTIGATOR and/or INSTITUTION in the course of the Study and in accordance with the Protocol, excluding Institution Inventions, Other Inventions, GRANTOR Inventions and GRANTOR Background IP as defined below ("**Data**") shall be owned by and be the property of the INSTITUTION. GRANTOR is hereby granted a non-exclusive, free of charge, perpetual, world-wide, sublicensable (but only to its Affiliates) right and license to access, copy and use the Study Report for any legitimate purpose subject to Section 7.4 (Use of Name) provided that GRANTOR will not publicly disclose the final report until publication of the Data by INSTITUTION and/or INVESTIGATOR and/or confirmation by INSTITUTION and/or INVESTIGATOR that there will not be a publication.

(c) INSTITUTION and INVESTIGATOR shall own all right, title, and interest in any invention created by INSTITUTION and/or INVESTIGATOR arising out of their performance of the Study a (together with patent rights covering such inventions hereafter referred to as "**INSTITUTION Invention**").

(d) INSTITUTION shall own all right, title and interest in any invention created by INSTITUTION and/or INVESTIGATOR arising out of performance of the Study and which directly relies on GRANTOR Background IP (together with patent rights covering such inventions, each hereafter also referred to as "Institution **Invention**"), All right, title and interest in and to Study Inventions shall reside jointly if both Institution and GRANTOR are inventors. Inventorship shall be determined in accordance with US Patent Laws ("Joint Invention") . With respect to Joint Inventions, each Party hereby confirms that nothing in this Agreement shall operate in any way to limit the other Party's indivisible, non-exclusive ownership interest in and to such Joint Inventions.

To the Extent that INSTITUTION owns the rights to any such INSTITUTION INVENTIONS, Institution grants GRANTOR a fully paid up, limited, royalty free, non-exclusive, non-sublicensable license to use Institution Invention for GRANTOR's internal research and development purposes.

4251048 - CCF LAW DEPARTMENT APPROVED AS TO FORM - ACD - 9/9/2021

To the extent that Institution owns the rights to any such Study Device related INSTITUTION INVENTION, INSTITUTION grants to GRANTOR a time-limited option to obtain an exclusive worldwide, royalty-bearing license for all purposes, to INSTITUTION's rights to sublicense and assign, to each Study Device related INSTITUTION INVENTION or Joint Invention, under terms to be negotiated in good faith between the Parties. This option shall extend for ninety (90) days after GRANTOR's receipt of a written, confidential disclosure of such Inventions by Institution ("Option Period"). All licenses shall be subject to the standard terms and conditions of INSTITUTION's licenses as well as to the negotiation and mutual agreement of other licensing terms, including terms requiring GRANTOR to demonstrate reasonable efforts to commercialize the licensed Invention. Any such exclusive licenses will include a commercially reasonable royalty rate at the time of the conception and reduction to practice of any such Study Device related Institution Invention or Joint Invention. In the event that GRANTOR fails to so notify INSTITUTION or elects not to obtain an exclusive license, then GRANTOR'S option shall expire with respect to said Study Device related INSTITUTION Invention or Joint Invention, and INSITUTION shall have no further obligation to Grantor regarding such license. Upon GRANTOR's exercise of its option with regard to any particular Study Device related Institution Invention or Joint Invention, INSTITUTION and GRANTOR shall negotiate in good faith for up to six (6) months in an attempt to reach a license agreement satisfactory to both parties. If the parties fail to negotiate a license within six (6) months after the beginning of the negotiation period ("Negotiation Period"), Institution shall have no further obligation to GRANTOR regarding such exclusive license.

(e) Neither GRANTOR nor INSTITUTION transfers to the other by operation of this Agreement any patent, copyright, or other proprietary right of any Party, except as explicitly stated in this Agreement.

(f) INSTITUTION makes no representations or warranties regarding, the Study Device any merchantability of the Study Device, Study Results, Study Data or Study Inventions or the fitness of the Study Results, Study Data or Study Inventions for any particular purpose. INSTITUTION shall not be liable for any direct, special, indirect, punitive, consequential or other damage suffered by GRANTOR or others resulting from the development or use of the Study Results, Study Data or Study Inventions, Study Device regardless of the foreseeability thereof. INSTITUTION does not warrant that the Study Results, Study Results, Study Data, Study Device or Study Inventions or any part thereof or any aspect of the same shall be capable of receiving statutory protection. INSTITUTION FURTHER DOES NOT MAKE ANY WARRANTIES WITH REGARD TO THE FREEDOM FROM PATENT, TRADEMARK, OR COPYRIGHT INFRINGEMENT, INFORMATIONAL CONTENT, INTEGRATION, OR THEFT OF TRADE SECRETS AND DOES NOT ASSUME ANY LIABILITY HEREUNDER FOR ANY INFRINGEMENT OF ANY PATENT, TRADEMARK, OR COPYRIGHT ARISING FROM THE USE OF STUDY DATA, STUDY RESULTS, STUDY DEVICE OR INVENTIONS.

6.3 <u>INFORMED CONSENT</u>

To the extent required by Applicable Laws, INSTITUTION shall cause INVESTIGATOR to obtain advance informed consent, i.e. prior to any Study procedures being performed, from each of the subjects (or their duly authorized representatives) participating in the Study. INSTITUTION shall cause INVESTIGATOR to comply with Applicable Laws when obtaining Study subjects' consent to participation in the Study. Without limiting the foregoing, with respect to the Study, INSTITUTION shall ensure that INVESTIGATOR requires that all informed consent documents:

(a) comply with all Applicable Laws, including, without limitation, regulations specifying required ICF elements; and

(b) adequately describe all foreseeable risks associated with participating in the Study (including all foreseeable material risks associated with Study Device) in a manner consistent with the investigator brochure(s).

6.4 COLLECTION AND STORAGE OF DATA AND MEDICAL RECORDS

As necessary, INSTITUTION shall ensure that INVESTIGATOR prepares, documents and maintains medical records and Data in accordance with Applicable Law, INSTITUTION policy and the Protocol.

ARTICLE 7. **PUBLICATION**

7.1 PUBLICATION BY INVESTIGATOR AND INSTITUTION

Each of INSTITUTION and INVESTIGATOR may freely publish and disseminate the Study results and shall solely determine the authorship and contents (including scientific conclusions and professional judgments) of same. INSTITUTION shall and shall cause INVESTIGATOR to provide GRANTOR with a copy of each Publication not less than thirty (30) days prior to its submission to a journal, publisher or meeting or fifteen (15) days prior to any public disclosure of any manuscript or other public disclosure (e.g., presentations). "**Publication**" means any public disclosures by INSTITUTION and any of its respective Study Personnel of the Study results and shall include, without limitation, any abstract, article, manuscript, data, text, diagrams, posters, charts, slides or pictures related to the investigative findings. If identified by GRANTOR, all Confidential Information belonging to GRANTOR that may be contained in any Publication shall be deleted as long as its removal does not compromise the scientific integrity of the publication., save for Study methods and any background information provided by GRANTOR that is necessary to include in any Publication of Study results, or necessary for other scholars to verify such Study results. GRANTOR acknowledges that INSTITUTION retains editorial control of all publications.

7.2 DELAY FOR PATENT PROTECTION

In addition, if requested by GRANTOR, INSTITUTION shall, and shall cause all Study Personnel to, withhold a Publication from submission for publication or presentation for an additional sixty(60) days from the date is first received by GRANTOR for review to allow for the filing of a patent application or the taking of any other measure to preserve GRANTOR's proprietary rights.

7.3 LICENSE TO USE PUBLICATIONS

Subject to the rights of any third party publisher, INSTITUTION hereby grants to GRANTOR an irrevocable, royalty-free license to make, distribute and otherwise use copies of any Publication such use of any Publication is in accordance with Section 7.4 . . In addition, to the extent applicable, GRANTOR personnel shall be acknowledged in accordance with customary scientific practice.

7.4 USE OF NAME

Neither Party shall make, place or disseminate any advertising, public relations, promotional material or any material of any kind using the name of the other Party and/or the other Party's' subsidiary or affiliate companies or using their trademarks, logos, symbol, image, likeness, abbreviation and/or other identifying intellectual property of the other Party or the other Party's employee or agent, without the prior written approval of such Party. The use of Institution's name, trademark, logo, symbol, image or the like shall require written permission from Cleveland Clinic Foundation's Corporate Communications Executive Director. Notwithstanding the foregoing, INSTITUTION and INVESTIGATOR may, without prior consent, disclose their participation in the Study (including the name of GRANTOR, name of the Study, protocol

number, and funding amount) as required by law, Court order, or regulation; and may, without prior consent, disclose their participation in the Study (including the name of GRANTOR, name of the Study and protocol number), in (a) INVESTIGATOR's C.V.; (b) internal reports; (c) publications and presentations made in accordance with this Agreement; (d) grant applications to government funding sources; (e) government reports and filings; and (f) conflict-of-interest reports. This paragraph also applies to SUBINVESTIGATORS and other Study Personnel. Institution may also (i) acknowledge GRANTOR's support, including but not limited to financial support as may be required by academic journals, professional societies, funding agencies, and applicable regulations and (ii) post GRANTOR's name, the Study title, the Study period, and other publically available information about the Study, including that listed on clinicaltrials.gov, on Institution publicly accessible lists of research conducted by the Institution.

ARTICLE 8. **TERM AND TERMINATION**

8.1 TERM

This Agreement shall commence on the Effective Date of this Agreement and shall, unless sooner terminated as expressly provided in this Agreement, continue in full force and effect up through and shall expire on the tenth anniversary of the Effective Date.

8.2 TERMINATION BY PARTIES

(a) This Agreement may be terminated or suspended and/or further enrollment of subjects in the Study may be suspended:

(i) by GRANTOR without cause upon 30 days prior written notice to INSTITUTION;

(ii) by any Party, (a) if necessary to protect the best interests of the Study subjects, (b) for material breach of this Agreement where the breach is not cured within thirty (30) days following receipt of written notice thereof from the non-breaching Party, or (c) as otherwise expressly permitted by the Protocol;

(iii) by written mutual agreement of the Parties;

(iv) Either Party may terminate this Agreement immediately in the event that the other Party becomes insolvent or is unable to pay its debts as they become due, or a petition in bankruptcy or for reorganization is filed by or against it, or a receiver is appointed of the whole or any substantial portion of its property; or either PARTY may terminate this Agreement immediately if the Study cannot be initiated or continued because of IRB disapproval.

(v)

(b) Termination of this Agreement (and GRANTOR's research support) shall not affect INSTITUTION's or INVESTIGATOR's right to continue the Study.

(c) This Agreement may be terminated by INSTITUTION with 30 days prior written notice to GRANTOR.

8.3 SURVIVAL

The rights and obligations of GRANTOR and INSTITUTION which by intent or meaning, express or implied, have validity beyond termination or expiration of this Agreement shall survive the termination or expiration of this Agreement.

4251048 - CCF LAW DEPARTMENT APPROVED AS TO FORM - ACD - 9/9/2021

ARTICLE 9. **MISCELLANEOUS**

9.1 INDEMNIFICATION

GRANTOR shall defend, indemnify and hold harmless INSTITUTION and the directors, officers, medical and professional staff, employees, students, associates, agents and its employees and successors, heirs and assigns (each an "**Indemnitee**" and collectively the "**Indemnitees**") from and against any and all liabilities, claims, suits, damages, costs and expenses, including reasonable legal fees, (hereinafter "**Losses**") arising out of (a) GRANTOR's use of the Study Report; (b) any patent infringement, copyright violation, or trade secret misappropriation caused by use of the Study Drug; (c) the design, manufacture, sale, promotion or use in commerce by GRANTOR, its Affiliates or its and their its licensee of any product, service or process relating to the Study; (d) any negligence or willful misconduct or wrongful acts or omissions of GRANTOR and (e) any defect in the manufacture of the Study Drug. The indemnification given by GRANTOR is given individually to each of the Indemnitees such that any failure by an Indemnitee to comply with the indemnification provisions in respect of its/his/her interests will not adversely affect, deprive or otherwise prejudice the right of each of the other Indemnitees from obtaining indemnification from GRANTOR.

INSTITUTION will defend, indemnify and hold harmless GRANTOR, its Affiliates and their respective directors, officers, employees, agents, successors, and assigns from and against any Losses arising out of the negligence or wrongful acts or omissions of INSTITUTION or the employees, officers, or directors of INSTITUTION except to the extent such liability, loss or damage is caused by the negligence or willful misconduct of GRANTOR, its Affiliates each of their respective directors, officers, employees, agents, successors, and assigns, or the breach of this Agreement by GRANTOR, its Affiliates or its and their respective directors, officers, employees, agents, successors, and assigns

9.2 INDEPENDENT CONTRACTOR RELATIONSHIP

Nothing contained in this Agreement shall be construed to place the Parties or their personnel in the relationship of employer and employee, partners, principal and agent, joint venturers or as an insurer or a representative of the other Party. No Party shall have the power to bind or obligate any other Party nor shall any Party hold itself out as having such authority. INSTITUTION and Study Personnel shall not (i) be eligible to participate in or receive any benefits or have any rights as an employee of GRANTOR; or (ii) be covered by any GRANTOR liability insurance policies during the term of this Agreement.

9.3 INSURANCE

(a) INSTITUTION represents and warrants that it has and shall maintain comprehensive general liability insurance coverage on either a self-insured or indemnity basis to protect against liability under this Agreement in amounts equal to at least one million dollars ($1,000,000) per occurrence combined single limit and three million dollars ($3,000,000) annual aggregate and, upon request of the GRANTOR , agrees to furnish evidence of insurance acceptable to the other Party indicating the required coverage. GRANTOR agrees to give Institution at least thirty (30) days prior written notice in the event of any material, adverse change in such insurance.

(b) GRANTOR carries clinical trials liability insurance and product liability insurance, each with limits of at least $5,000,000 per occurrence, per annum, and $10,000,000 in the aggregate, per annum. GRANTOR's insurance includes blanket contractual liability, covers the Study and is not materially encumbered by existing claims. GRANTOR shall maintain such coverage for the duration of this Agreement and, if the policy is claims-made, for two (2) years thereafter.

9.4 NO SPONSORSHIP OR INJURY COMPENSATION

Under no circumstances shall GRANTOR be deemed the "sponsor" of the Study, as that term is defined under Applicable Law. Except as provided in Section 9.1, above, GRANTOR is not obligated to provide any compensation for any research-related injuries, and INSTITUTION shall not and shall cause its respective Study Personnel not to, state in any Protocol or written or other informed consent that GRANTOR will provide any such compensation.

9.5 NO CONFLICT

INSTITUTION hereby represents and CERTIFIES that the execution, delivery and performance of this Agreement: (i) does not, in any material respect, conflict with or violate any Applicable Law; (ii) does not conflict with or violate any organizational, charter or internal governance document of INSTITUTION; and (iii) does not conflict with or constitute a default under any contract, agreement or obligation of INSTITUTION.

9.6 NO IMPLIED RIGHTS OR LICENSE

No right or license is granted under this Agreement by any Party to the other Parties except those specifically set forth in this Agreement. Nothing contained within this Agreement shall impose an obligation of exclusivity on any of the Parties.

9.7 GOVERNING LAW : INTENTIONALLY OMITTED

9.8 THE PARTIES REMAIN SILENT ON JURISDICTION.SEVERABILITY

This Agreement is intended to be severable, and the invalidity and/or unenforceability of any clause of this Agreement, or part thereof, shall not affect the validity and or enforceability of any other clause or part thereof to the extent not invalidated or held unenforceable.

9.9 NOTICES

Any legal or formal notices must be in writing and will be deemed effective only when delivered personally or mailed by certified or registered mail, postage prepaid, to the Party and address set forth below.

For purposes of this Agreement, the person at GRANTOR to whom notices shall be addressed is:

Dr. Rahul Samat
Rehabtronics Inc.
4350 - 10230 Jasper Ave.
Edmonton, AB, Canada T5J 4P6
Phone: 1-800-481-3214 Ext. 700
Email: rahul@rehabtronics.com

And the person at INSTITUTION to whom notices shall be addressed is:

The Cleveland Clinic Foundation
Law Department
Administration Campus Building 3
3050 Science Park Drive
Beachwood, OH 44122

With a copy to INVESTIGATOR

Chase Donaldson, MD
The Cleveland Clinic Foundation
9500 Euclid Avenue/P77
Cleveland, OH 44195
DONALDC@ccf.org

Each Party may change its address for notices by giving the other Parties written notice thereof.

9.10 ENTIRE AGREEMENT; CONFLICT

This Agreement and any attachments hereto/thereto set out the entire agreement of the Parties with respect to its subject matter and supersede all prior agreements and understandings relating to its subject matter of this Agreement. This Agreement and any attachments hereto/thereto may not be altered, modified, or waived in whole or in part, except in writing signed by the Parties.

9.11 HEADINGS

The headings in this Agreement are intended solely for convenience or reference and shall be given no effect in the construction or interpretation of this Agreement.

9.12 COUNTERPARTS

This Agreement may be executed in counterparts, each of which shall be deemed to be an original, and all of such counterparts shall together constitute one and the same agreement. Each Party acknowledges that an original signature or a copy thereof, including a "portable document format" or PDF copy, or a signature generated by industry standard electronic signature software, which is transmitted by facsimile or by email shall constitute an original signature for purposes of this Agreement and shall have the same legal force and effect as the exchange of original signatures, and that in any proceeding arising under or relating to this Agreement, each Party hereby waives any right to raise any defence or waiver based upon execution of this Agreement by means of such electronic signatures or maintenance of the executed Agreement electronically.

9.13 FORCE MAJEURE

For the purposes of this Agreement, "Force Majeure" means circumstances and conditions beyond the control of the party affected and without the fault or negligence of such party which render it impossible for that party to fulfill its obligations under this Agreement or which delay such fulfillment. Force Majeure shall include, but not be limited to, declared or undeclared war or warlike acts, acts of God, acts by a foreign enemy, civil war, confiscation by order or directive of any government or public authority, pandemic or similar form of epidemic, pandemic, earthquake, flood, fire or other natural physical disaster, strikes, labour unrest, lockouts or other matters similar in nature or severity to the foregoing.

No Party shall be liable for any delay or failure to perform its obligations hereunder, nor be deemed to be in breach of this Agreement, if and to the extent such delay or failure has arisen from a Force Majeure. The occurrence of a Force Majeure shall not release the affected party from its obligations hereunder, but shall merely suspend the performance of any obligation so prevented, hindered or delayed during the period of continuance of the Force Majeure. The Party alleging a Force Majeure shall notify the other parties in writing as soon as reasonably practicable upon obtaining knowledge of the occurrence of the Force Majeure which notice shall include particulars of the Force Majeure including the anticipated duration thereof. The party affected shall exert its reasonable efforts to eliminate or cure or overcome any of the causes of the

4251048 - CCF LAW DEPARTMENT APPROVED AS TO FORM - ACD - 9/9/2021

Force Majeure and to resume performance of its obligations with all possible speed, but shall not be required to settle any labour dispute in order to comply with this Agreement.

9.14 ASSIGNMENT

No Party may assign any of its/his/her respective rights or delegate any of its/his/her respective duties under this Agreement without the prior written consent of the other Parties. Subject to the foregoing, this Agreement shall bind and inure to the benefit of the respective Parties and their successors, heirs and assigns.

*[Signature Page Follows]*The Parties hereto confirm that it is their wish that this Agreement be drawn up in the English language. Les parties aux présentes confirment leur volonté que la présente convention soit rédigée en anglais.

In order to demonstrate their agreement, the Parties have executed this Agreement as of the Effective Date. This agreement may be signed in any manner that clearly evidences the Parties' intent to be bound, including via faxed or, images, electronic or digital signatures (i.e. pdf)

GRANTOR

By: [signature]

Print Name: Dr. Rahul Samant

Title: CEO, Rehabtronics Inc.

Date: September 28, 2021

THE CLEVELAND CLINIC FOUNDATION

By: Serpil Erzurum/JW

Print Name: Serpil C. Erzurum, MD

Title: Chair, Lerner Research Institute

Date: 9/23/2021

Read and Acknowledged by: INVESTIGATOR(Not a Party to the Agreement)

By: [signature]

Print Name: Chase Donaldson, MD

Title: Principal Investigator

Date:____

Attachment A:

Rehabtronics – PROTECT2 Study Budget and Milestone Payments:

Study Budget :

Study Name PROTECT-2

Principal Investigator Chase Donalson

	Activity	Hourly Rate	Number of Hours	Total
**Per Patient**				
	Principal Investigator	**$350**	**4**	**$1,400**
	Research Fellow	**$60**	**10**	**$600**
	Quality Assurance	**$55**	**2**	**$110**
			**Per Patient**	$2,110
		Sample Size	300	**$633,000**
**Patient Stipends**		$0	300	**$0**
**Database Development and Maintenance**				**$10,000**
**Protocol Development**				**$15,000**
**Statistical Analysis and Manuscript**				**$30,000**
**Direct Cost Total**				$688,000
**CCF 30% Indirect Cost**		30%		$206,400
**Total**				**$894,400**

Milestone Payments Schedule :

Milestone 1	Initiation (start up)	$50,000
Milestone 2	50 patients	$132,400
Milestone 3	100 patients and Interim analysis	$132,400
Milestone 4	150 patients	$132,400
Milestone 5	200 patients and Interim analysis	$132,400
Milestone 6	250 patients	$132,400
Milestone 7	300 patients Last patient	$132,400
Milestone 8	Final report	$50,000
		$894,400

Attachment B:

Protocol. The PROTECT 2 ICU Study: Pressure Injury Treatment by Intermittent Electrical Stimulation: A Randomized, Controlled Trial

ARTICLE 10. **Protocol.PROTECT2.20210706**

Version 1.1

Adapted from The PROTECTStudy: Pressure injuRy Treatment by inTermittent EleCtrical sTimulation (IIT-0018 April 8, 2020)

Short Title	PROTECT 2:ICU
Regulatory Sponsor<br><br>Corporate Sponsor	The Cleveland Clinic Foundation<br><br>Rehabtronics, Inc.<br>https://www.blog.rehabtronics.com
Principal Investigator	Chase Donaldson, MD
Collaborators (alphabetical)	Eric Chanowski, MD<br>Hani Essber, MD<br>Faith Factor, MD<br>Eric Harvester, CNP<br>Steven Insler, DO<br>Nakul Kumar, MD<br>Howard Nearman, MD<br>Fabio Rodriguez Patarroyo, MD<br>James Rowbottom, MD<br>Lisa Shuck, RN<br>Roshni Sreedharan, MD<br>Belinda Udeh, MD<br>Chiedozie Udeh, MD<br>Shinya Unai, MD<br>Andrea Kurz, MD

4251048 - CCF LAW DEPARTMENT APPROVED AS TO FORM - ACD - 9/9/2021

	Ashish Khanna, MD

4251048 - CCF LAW DEPARTMENT APPROVED AS TO FORM - ACD - 9/9/2021

ARTICLE 11. **STUDY SUMMARY/SYNOPSIS**

Title The PROTECT 2 ICU Study: Pressure Injury	Treatment by Intermittent Electrical Stimulation: A
Randomized, Controlled Trial	
Short Title Protocol	PROTECT 2 ICU Study
Number	PROTECT IIT-0018 Phase III
Phase	Phase III
Methodology	Multicentered randomized controlled study with
adaptive design to evaluate if Intermittent	Electrical Stimulation (IES) decreases progression and
facilitates healing of pressure injuries for patients	with, sacral / ischial pressure injuries.
	Accrual: 18 Months
Study Duration Treatment: At least 30 days of	treatment OR death OR hospital discharge.
	Follow up: 3 and 12 Months
Total Study Duration: Approximately 24	Months
Study Centre(s)	Cleveland Clinic Foundation (CCF) Main Campus
Wake Forest University	University of Graz
Primary Objectives Determine the superiority of an	IES system added to the standard care of turning patients every two hours
in sacral and ischial pressure injury score	measured over time.
	Patients receiving IES treatment concurrent with the

4251048 - CCF LAW DEPARTMENT APPROVED AS TO FORM - ACD - 9/9/2021

Secondary Objectives standard of care will be compared to standard care for the following: time to resolution of ulcer, time to worsening of ulcer, time to discharge alive, and mortality. Describe adverse events and their frequency caused by IES system. Treatment group heterogeneity will be assessed for the following patient factors: Ulcer stage at enrollment (Stage 1 or Stage 2), hypoalbuminemia (>4, 3-4, 2-3, <2), Diagnosis of diabetes mellitus (y/n), Hemoglobin A1C (HbA1C) (>10, 8-9.9, 6-7.9, <6.), COVID positive during hospital stay, steroid use for study period (y/n), continuous renal replacement therapy (y/n) pressure injury risk by Braden Scale score: Moderate (13-14), High (10-12), Very High (9 or less), BMI (<25, 25-29.9,

4251048 - CCF LAW DEPARTMENT APPROVED AS TO FORM - ACD - 9/9/2021

30-39.9), vasopressor dose at enrollment, number of ventilator days (0-7, 7-21, >21), number of hypoxic

4251048 - CCF LAW DEPARTMENT APPROVED AS TO FORM - ACD - 9/9/2021

events while using device, highest SOFA score for study period cumulative fluid balance at enrollment, vasopressors used and dosages, ICU length of stay, number of units of packed red blood cells transfused while on the device, average hemoglobin over the duration of the device, minimum hemoglobin while on the device, average blood oxygen saturation while on the device, number of hypotensive episodes while on the device, mean blood glucose while on the device.

IES will be compared to standard of care in the following specific patient populations on the primary outcome: New ventricular assist device (VAD), ECMO, organ transplantation.

Exploratory Objectives Assess cost effectiveness analysis within a sub-study of data generated. We will measure incremental cost-effectiveness ratios (ICERS) between traditional care and IES interventions in the treatment of pressure ulcers.

Study Sample Size n = 1200; 1:1 randomization to each arm by facility

Study population Multidisciplinary ICU patients with new or established stage 1 or 2 pressure ulcers; patients can be accrued at any point during their admission.

Investigational product, treatment or intervention Intermittent electrical stimulation system. Charged pulses will be administered to bilateral gluteus maximus through surface electrodes. Stimulation occurs at 30 Hz for 10s every 10 minutes. The intervention is administered 24/7 while in the ICU except as required for intermittent patient care tasks. This device is added to the standard of care management.

Device Class and Manufacturer Class II

Rehabtronics, Inc.
#4352, 10230 Jasper Avenue Edmonton, Alberta, Canada T5J 4P6 Phone: (+1) 780-701-5167
Email: support@rehabtronics.com

4251048 - CCF LAW DEPARTMENT APPROVED AS TO FORM - ACD - 9/9/2021

Duration of administration	Study entry until at least 30 days of treatment OR hospital discharge OR death.

4251048 - CCF LAW DEPARTMENT APPROVED AS TO FORM - ACD - 9/9/2021

Reference therapy (control) Standard of Care: turning the patient every two hours as per standard inpatient nursing practice and any other wound care or plastic surgery treatments deemed appropriate as per usual care.

Statistical Methodology

Primary Endpoint: Patients receiving IES treatment concurrent with the standard of care will be compared to the standard of care alone for patients with new or established stage 1 or 2 sacral or ischial pressure ulcer using repeated measures of ulcer staging over time. .

Secondary Endpoints: Assess the treatment effect of IES vs standard care on time to events outcomes (time to resolution of ulcer, time to worsening of ulcer, time to discharge alive, and mortality) using Cox proportional hazards regression and reporting results as hazard ratio and 95% confidence interval, with the proportional hazards assumption tested using the treatment-by-log(time) interaction as well as graphical displays of the hazard of the outcome over time for the overlayed treatment groups. Kaplan-Meier analyses with 95% confidence bands and the log-rank test will also be used. Assess for treatment effect heterogeneity for the primary outcome across levels of various baseline factors using tests for interaction (treatment-by-baseline factor.

Exploratory Endpoints: Measure incremental costeffectiveness ratios (ICERS) between traditional care and IES interventions in the treatment of pressure ulcers

Cost effective analysis will be done use TreeAgePro for Healthcare to measure incremental cost effectiveness ratio between the groups.

ARTICLE 12. **TABLE OF CONTENTS**

INTRODUCTION.. 9

1.1 Background...................................... 10

1.2 Study Rationale 15

4251048 - CCF LAW DEPARTMENT APPROVED AS TO FORM - ACD - 9/9/2021

STUDY OBJECTIVES AND OUTCOMES MEASURES/ ENDPOINTS 16

2.1 Objectives 16

2.2 Outcome Measures/Endpoints 16

STUDY DESIGN 17

3.1 Description of the Population to be Studied 17 3.2

Trial Duration 17

PATIENT SELECTION..18

4.1 Inclusion Criteria 18

4.2 Exclusion Criteria 18

4.3 Randomization Criteria...18

4.4 Withdrawal/Discontinuation Criteria.. 18

STUDY TREATMENT 19

5.1 Treatment Allocation 19

5.2 Intervention 19

5.3 Medications/Treatments Permitted 20

5.4 Medications/Treatments Prohibited/Restricted...20

STUDY VISITS AND PROCEDURES...20

6.1 Informed Consent 20

6.2 Patient Enrollment 21

6.3 Study Visits.. 23

STATISTICAL CONSIDERATIONS 23

7.1 Analysis…..23

7.2 Interim Analysis..25

7.3 Sample Size Calculation………………………………………………………… 25

ADVERSE EVENT REPORTING 26

4251048 - CCF LAW DEPARTMENT APPROVED AS TO FORM - ACD - 9/9/2021

8.1 Definition of an Adverse Event.. 26

8.2 Reporting, Recording and Follow-up of Adverse Events

....27 8.3 Serious Adverse

Events...27

9 MONITORING, AUDITING AND INSPECTING..28

9.1 Source Data 28

9.2 Quality Control and Assurance..29

9.3 Data Safety Monitoring Board 29

9.4 Deviations.. 29

10 DATA COLLECTION AND DATA MANAGEMENT..29

10.1 Data Collection/Case Report Forms (CRFs).. 29

11 ADMINISTRATIVE, ETHICAL AND REGULATORY STANDARDS 29

11.1 Compliance Statement 29

11.2 Ethics.. 29

11.3 Confidentiality and Data Protection 30

11.4 Protocol Registration.. 30

11.5 Protocol

Amendments...

30 12 CRITERIA FOR TERMINATION OF THE TRIAL

....31

13 FINANCING AND INSURANCE 32

14 PUBLICATION POLICY 32

15 PRINCIPAL INVESTIGATOR PROTOCOL AGREEMENT 33

16 REFERENCES.. .34

17 APPENDICES.. .36

17 CONSENT FORM……………………………………………………………………46

ARTICLE 13. LIST OF ABBREVIATIONS

AE	Adverse Event
CCF	Cleveland Clinic Foundation
CRF	Case report form
CTCAE	Common Terminology Criteria for Adverse Events
CTU	Clinical Trials Unit
DSMB	Data Safety Monitoring Board
DTI	Deep Tissue Injury
GCP	Good Clinical Practice
ICH	International Conference on Harmonization
IEC	Independent Ethics Committee

4251048 - CCF LAW DEPARTMENT APPROVED AS TO FORM - ACD - 9/9/2021

IES	Intermittent Electrical Stimulation
IIT	Investigator Initiated Trials
IRB	Institutional Review Board
REB	Research Ethics Board
SAE	Serious Adverse Event
SPI	Sacral and Ischial Pressure Injuries

The term 'Participant' is used in the protocol to refer to a person who has consented to participate in the clinical research study.

13.0 1.INTRODUCTION

Pressure ulcers (also known as pressure injuries or bedsores) constitute a major morbidity in critically ill patients due to immobilization, deranged tissue perfusion, and poor nutrition with associated poor wound healing. It has been estimated that the prevalence of pressure ulcers in ICU patients is 26.6 percent, (Labeau et al., 2020) significantly contributing to nosocomial risk and healthcare costs. Pressure ulcers can develop within the first hours of acute care despite significant research and quality recommendations in preventive care. (Gefen, 2008) With the ongoing coronavirus pandemic and increased demand for artificial life support with associated ICUrelated immobilization, new methods to reduce the development of pressure ulcers are needed. (Tang, Li, Gong, Li, & Yang, 2020) (Moore et al., 2020) The proposed randomized, controlled trial addresses ***intermittent electrical stimulation (IES***), a novel, non-invasive technology to enhance tissue perfusion to prevent pressure ulcers in the ICU.

Specific Aims:

This study will determine the superiority of an IES system added to the standard care of turning patients every two hours in treating sacral and ischial pressure injuries.

1.a. Primary Endpoint: Patients receiving IES treatment concurrent with the standard of care will be compared to the standard of care alone for sacral and ischial pressure injury score over time.

2.a. Secondary Endpoints: Patients receiving IES treatment concurrent with the standard of care will be compared to standard care for the following: time to resolution of ulcer, time to worsening of ulcer, time to discharge alive, and mortality. Describe adverse events and their frequency caused by IES system. Treatment group heterogeneity will be assessed for the following patient factors: Ulcer stage at enrollment (Stage 1 or Stage 2), hypoalbuminemia (>4, 3-4, 2-3, <2), Diagnosis of diabetes mellitus (y/n), Hemoglobin A1C (HbA1C) (>10, 8-9.9, 6-7.9, <6.), COVID positive during hospital stay, steroid use for study period (y/n), continuous renal

4251048 - CCF LAW DEPARTMENT APPROVED AS TO FORM - ACD - 9/9/2021

replacement therapy (y/n) pressure injury risk by Braden Scale score: Moderate (13-14), High (10-12), Very High (9 or less), BMI (<25, 25-29.9, 30-39.9), vasopressor dose at enrollment, number of ventilator days (0-7, 7-21, >21), number of hypoxic events while using device, lowest SOFA score for study period, . IES will be compared to standard of care in the following specific patient populations: New ventricular assist device (VAD), ECMO, organ transplantation.

2.b. Exploratory Endpoints: Compare IES to standard care for comparative cost effectiveness. Assess cost effectiveness analysis within a sub-study of data generated. We will measure incremental cost-effectiveness ratios (ICERS) between traditional care and IES interventions in the treatment of pressure ulcers.

13.1 HYPOTHESES AND RESEARCH QUESTION

Our hypothesis is that use of IES with standard intensive nursing care will reduce the risk of progression for pressure ulcers in patients admitted to intensive care._This study is germane in that it will provide level 1 evidence on the effectiveness of intermittent electrical stimulation on the prevention of ICU associated pressure ulcers. It has the potential to minimize iatrogenic harm from this common ICU associated morbidity and improve overall supportive skin care.
It has the potential to significantly improve the intensive care and influence clinical supportive care for this common, ICU associated morbidity.

13.2 IMPORTANCE, NATURE AND RATIONALE OF THE RESEARCH

Despite increased understanding of pathological mechanisms and medical advances, pressure ulcers remain problematic in critical care and new approaches are required. This research evaluates an innovative technology to increase tissue perfusion to high-risk anatomical areas. This study adapts a prior research protocol tested in oncological patients for a focus on intensive care patients. The study with its randomized, controlled design will provide level 1 evidence of the effectiveness of this novel technology in the treatment of pressure ulcers. A positive (or negative study) on pressure ulcer risk may provide societal benefits.

As a randomized, controlled trial this study offers the potential to support this novel technology, limit a major nosocomial morbidity, and influence the practice of critical care.

13.2.1. 1.1 Background

Pressure injuries, also known as pressure ulcers or bedsores, are common afflictions that impact people with limited mobility and sensation. They are localized injuries to the skin and/or underlying tissues generally caused by pressure or shear near bony prominences (*National Pressure Ulcer Adivsory Panel, European Pressure Ulcer Advisory Panel and Pan Pacific Pressure Injury*

Alliance. Prevention and Treatment of Pressure Ulcers: Quick Reference Guide, 2014) (1).
Pressure injuries are a significant problem in older hospitalized adults and have an estimated

26% prevalence across all healthcare institutions in Canada (Woodbury & Houghton, 2004) (2). In the United States of America, pressure ulcers are responsible for over 60,000 deaths each year, and the treatment of hospital-acquired pressure ulcers costs ~$12 billion annually (Zulkowski, Langemo, & Posthauer, 2005) (3).

There are six categories of pressure injuries: Stage I-IV, Unstageable, and Deep Tissue Injury (DTI) (*National Pressure Ulcer Adivsory Panel, European Pressure Ulcer Advisory Panel and Pan*
Pacific Pressure Injury Alliance. Prevention and Treatment of Pressure Ulcers: Quick Reference

Guide, 2014) (1). Staging is determined clinically, based on the type of tissue visualized or palpated (Fig. 1). Unstageable injuries are covered with eschar that obscures the wound bed. As eschar does not form at Stage I or II, Unstageable wounds conceal Stage III or IV injuries. Deep tissue injuries are indeterminate stages which reveal progression of a tissue injury, but the severity of which cannot yet be graded because of intact skin. The Braden Scale is a tool used to predict risk for developing hospital-acquired pressure injuries (Fig. 2). Categories of the scale are associated with various risk factors, including moisture, immobility, and nutrition. A score of 13-14 is considered Moderate Risk for developing a pressure injury; 10-12 is High Risk; a score of 9 or less is Very High Risk.

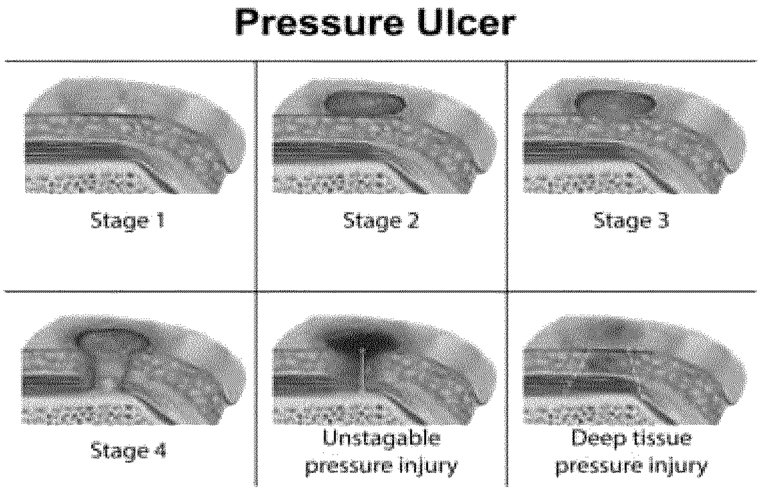

Figure 1 - Six categories of pressure injuries.

Braden Scale

Date of Assessment/Reassessment (day/month/year)							
**SENSORY PERCEPTION** ability to respond meaningfully to pressure-related discomfort	**1. Completely Limited:** Unresponsive (does not moan, flinch, or grasp) to painful stimuli, due to diminished level of consciousness or sedation. OR limited ability to feel pain over most of body surface.	**2. Very Limited:** Responds only to painful stimuli. Cannot communicate discomfort except by moaning or restlessness. OR has a sensory impairment which limits the ability to feel pain or discomfort over 1/2 of body.	**3. Slightly Limited:** Responds to verbal commands, but cannot always communicate discomfort or need to be turned. OR has some sensory impairment which limits ability to feel pain or discomfort in 1 or 2 extremities.	**4. No Impairment:** Responds to verbal commands, has no sensory deficit which would limit ability to feel or voice pain or discomfort.			
**MOISTURE** degree to which skin is exposed to moisture	**1. Constantly Moist:** Skin is kept moist almost constantly by perspiration, urine, etc. Dampness is detected every time patient is moved or turned.	**2. Very Moist:** Skin is often, but not always, moist. Linen must be changed at least once a shift.	**3. Occasionally Moist:** Skin is occasionally moist, requiring an extra linen change approximately once a day.	**4. Rarely Moist:** Skin is usually dry, linen only requires changing at routine intervals.			
**ACTIVITY** degree of physical activity	**1. Bedfast:** Confined to bed.	**2. Chairfast:** Ability to walk severely limited or non-existent. Cannot bear weight and/or must be assisted into chair or wheelchair.	**3. Walks Occasionally:** Walks occasionally during day, but for very short distances, with or without assistance. Spends majority of each shift in bed or chair.	**4. Walks Frequently:** Walks outside the room at least twice a day and inside room at least once every 2 hours during waking hours.			
**MOBILITY** ability to change and control body position	**1. Completely Immobile:** Does not make even slight changes in body or extremity position without assistance.	**2. Very Limited:** Makes occasional slight changes in body or extremity position but unable to make frequent or significant changes independently.	**3. Slightly Limited:** Makes frequent though slight changes in body or extremity position independently.	**4. No Limitations:** Makes major and frequent changes in position without assistance.			
**NUTRITION** *usual* food intake pattern	**1. Very Poor:** Never eats a complete meal. Rarely eats more than 1/3 of any food offered. Eats 2 servings or less of protein (meat or dairy products) per day. Takes fluids poorly. Does not take a liquid dietary supplement. OR is NPO and/or maintained on clear liquids or IV's for more than 5 days.	**2. Probably Inadequate:** Rarely eats a complete meal and generally eats only about 1/2 of any food offered. Protein intake includes only 3 servings of meat or dairy products per day. Occasionally will take a dietary supplement. OR receives less than optimum amount of liquid diet or tube feeding.	**3. Adequate:** Eats over half of most meals. Eats a total of 4 servings of protein (meat, dairy products) each day. Occasionally will refuse a meal, but will usually take a supplement if offered. OR is on a tube feeding or TPN regimen which probably meets most of nutritional needs.	**4. Excellent:** Eats most of every meal. Never refuses a meal. Usually eats a total of 4 or more servings of meat and dairy products. Occasionally eats between meals. Does not require supplementation.			
**FRICTION AND SHEAR**	**1. Problem:** Requires moderate to maximum assistance in moving. Complete lifting without sliding against sheets is impossible. Frequently slides down in bed or chair, requiring frequent repositioning with maximum assistance. Spasticity, contractures or agitation lead to almost constant friction.	**2. Potential Problem:** Moves feebly or requires minimum assistance. During a move skin probably slides to some extent against sheets, chair, restraints, or other devices. Maintains relatively good position in chair or bed most of the time but occasionally slides down.	**3. No Apparent Problem:** Moves in bed and in chair independently and has sufficient muscle strength to lift up completely during move. Maintains good position in bed or chair at all times.				
NOTE: Patients with a total score of 16 or less are considered to be at risk of developing pressure ulcers. (15 or 16 = mild risk. 13 or 14 = moderate risk. 12 or less = high risk)				TOTAL SCORE:			
				INITIALS:			

Figure 2 - The Braden Scale for pressure injury risk assessment.

The development of pressure ulcers in ICU patients significantly contribute to clinical risk, healthcare costs, and patient suffering. Typically developing in the sacral areas

of bedridden patients, pressure ulcers are associated with hypotension, presence and length of mechanical ventilation, renal replacement therapy, level of ICU sedation, and exposure to vasopressors. (Lima Serrano, González Méndez, Carrasco Cebollero, & Lima Rodríguez, 2017) (Lima Serrano et al., 2017) It has been reported that the United States spends $26.8 billion per year on treatment costs for pressure ulcers.

Intermittent electrical stimulation (IES) is a novel technology, though reported over thirty-five years ago, designed to increase tissue perfusion. In augmenting tissue perfusion with increased oxygen levels, it addresses a pathological mechanism associated with ICU associated pressure ulcers.(L. R. Solis et al., 2007) Other evidence-based ICU options to reduce the incidence of ICU pressure ulcers exist. A systematic review by Chou et al has supported that morbidity can be modified by advanced static support systems with reduced risk compared with standard mattresses, optimal nutritional support, repositioning processes, and skin care. (Chou et al., 2013) Limitations of current preventive practices require high intensity bedside personnel time, a concern in ICU's with limited personnel resources.

The inpatient population, by nature, is enriched in people at risk of pressure injuries. Limitations in mobility, hypoalbuminemia, decreased levels of consciousness, incontinence, and immunosuppression are all prevalent in this population, and increase the risk of pressure ulceration. Despite aggressive prevention efforts (including pressure redistribution mattresses and turning patients every 2 hours) and excellent training among inpatient nurses, pressure ulcers do occur. Additionally, patients transferred into the study site from other institutions may already have established pressure injuries. The study site participates in chart audit pressure injury reporting system of a small subset of patients, but the absolute frequency of the early-stage pressure injuries is not systematically collected nor reported. Inpatient physician and nursing staff report pressure injuries to be one of the most important unmet medical needs at the study site. Pressure injury risk and scoring is performed and charted every nursing shift for every patient (Appendices A & B). From discussion with inpatient nurses, the frequency of multiple ulcers is < 1%. Negative pressure wound therapy has not been employed within the last five years.

The treatment of pressure ulcers can be a challenging process and often requires prolonged hospitalization. In some cases, surgical interventions may be necessary to excise necrotic tissue and repair wounds with musculocutaneous flaps. The complexity and economic burden of treatment, coupled with the high morbidity and mortality associated with pressure ulcers, underscore the need for a robust method of prevention. Existing methods for prevention include pressure redistributing mattresses, wheelchair cushions, and offloading pressure by turning patients every 2 hours (Krause & Broderick, 2004) (Thomas, 2003) (4,5). Despite these interventions, the incidence and cost associated with pressure ulcers remain substantial.

The study intervention applies Intermittent Electrical Stimulation (IES) to the gluteus maximus muscles through surface electrodes. The device invokes muscle contractions for 10 seconds every 10 minutes, emulating the subconscious adjustments performed by able- bodied individuals in response to discomfort when seated or lying down. Animal studies demonstrate that IES reduces internal pressure at bone-muscle interfaces (the hypothesized mechanism for injury development), increases tissue oxygenation in surrounding areas, and reduces or eliminates deep tissue injury in muscles subjected to prolonged loading (Appendix C). (L. R. Solis et al., 2007) (L. Solis et al., 2008) Clinical studies support the safety, feasibility, and

general acceptability of gluteal IES in human participants (Ahmetovj et al., 2015) (Kane et al., 2017) (Appendix D). These pilot studies provide strong evidence for the capacity of IES to prevent sacral and ischial pressure injuries and support the need for a large, controlled, randomized controlled trial to demonstrate efficacy in the treatment of these injuries.
We propose a study to assess whether addition of the IES system to the standard of care reduces the morbidity of sacral and ischial pressure injuries by decreasing progression of stage 1 or stage 2 ulcers or facilitates their healing compared to standard wound care in an inpatient critical care population. This trial will also evaluate the impact of IES on duration of inpatient stay and overall survival. (L. Solis et al., 2008)

1.1.1 Non-Clinical Studies

The contractions produced by IES significantly reduce pressure around ischial tuberosities and distribute pressure to areas at lesser risk of breakdown from sustained loading (sitting or supine) (Gyawali et al., 2011). This reduction was not only significant for volunteers with intact spinal cords and healthy muscles, but in volunteers with long-term spinal cord injury (SCI) and substantial muscle atrophy. In addition, studies evaluating IES in adult pigs report significant reduction in internal pressure at deep bone-muscle interfaces (measured using minimally invasive catheters). (L. R. Solis et al., 2007) This finding is of major importance as deep tissue injuries (DTIs) originate at bone-muscle interfaces.

A study on anesthetized pigs loaded onto their buttocks compared the distribution of internal pressure at the ischial tuberosities and the effect of muscle contractions produced by IES (L. R. Solis, Twist, Seres, Thompson, & Mushahwar, 2013). Internal pressure was significantly higher than superficial pressure for all levels of loading, suggesting that pressure-mattress measurements substantially underestimate the magnitude of pressure around deep bone-muscle interfaces. Peak internal pressure was focused in a small region around the ischial tuberosities. Muscle contractions significantly reduced this pressure for all levels of loading on both healthy pigs and those with chronic SCI and muscle atrophy.

Validated functional Magnetic Resonance Imaging (fMRI) demonstrates IES contractions increase gluteal tissue oxygenation significantly for up to 15 minutes.(Gyawali et al., 2011) Increases in fMRI signal intensity were equivalent to an oxygenation increase of 28% in a rat model. These findings suggest IES may counteract the vascular mechanisms that lead to the formation of pressure injuries.

The ability of IES to prevent pressure ulcers was directly studied in animal models. Short, 2-hour experiments of anesthetized rats found that IES significantly reduced the extent and incidence of deep tissue injury compared to a no-treatment control (L. R. Solis et al., 2011). A similar experiment compared the efficacy of IES to a pressure relief strategy that emulated wheelchair push-ups for wheelchair-bound patients. Results supported the superiority of IES and demonstrated that the wheelchair push-ups method is similar in outcome to a no- treatment control group.(L. R. Solis et al., 2007) These findings indicate that active intermittent contractions of loaded muscles are paramount for injury prevention, and by improving perfusion, likely treatment as well.

1.1.2 Prior clinical studies with IES for prevention of pressure injury

4251048 - CCF LAW DEPARTMENT APPROVED AS TO FORM - ACD - 9/9/2021

A phase I clinical trial on 68 patients across five different clinical settings (ICU, acute ward, rehabilitation, LTC, and homecare) collected data regarding ease of use, comfort, and general acceptability of the IES system. (Ahmetović et al., 2015) No adverse effects related to the use of the IES device were seen in any study participants. Although skin redness localized to the electrode application was seen upon removal of the system in all participants, it fully resolved within 5 to 10 minutes. The system was easy to handle for clinical staff and caregivers, typically requiring less than 10 minutes to don and doff.
Study participants were highly satisfied and reported that the periodic contractions were neither bothersome nor disruptive; it was often easy to sleep with the system on (14). Participants reported relief from persistent pain, reduction of discomfort when sitting, and improved skin health. This trial demonstrated that barriers to adoption of the IES device are low and suggests that high level of compliance is expected in future trials due to participant interest in the device.

Ahmetovic et al. (2015) performed a trial on 48 participants across four clinical settings to assess the safety, feasibility, and acceptability of IES for the prevention of pressure injuries (8). The system proved safe and feasible in all settings, and no pressure injuries were observed in any of the participants. 97% of the patients' caregivers found that the IES system was an acceptable part of their daily routine.

Kane et al. (2017) investigated the feasibility, practicality, and end-user acceptability of IES as a method for pressure ulcer prevention in an intensive care environment (9). Twenty immobile subjects ranging from moderate to very high risk based on Braden Scale scores were enrolled. Nurses found that IES was simple to use and easy to incorporate into routine patient care. No pressure ulcers occurred in any subject, and no untoward reactions or adverse events occurred as a direct result of IES.

13.2.2. 1.2. Study Rationale

1.2.1 Rationale for conducting the study
Pressure injuries are highly prevalent in older hospitalized adults (2). The morbidity and mortality associated with pressure injuries, combined with the complexity of treatment, warrant aggressive prevention and treatment efforts. The studies highlighted above demonstrate the feasibility of the IES technology for prevention of pressure injuries, but no work has been done on the treatment of these injuries once manifested. The purpose of this trial is to assess the efficacy of an IES device on the treatment of sacral and ischial pressure injuries. Animal studies report that the addition of IES substantially reduces the risk and severity of pressure injuries in rats and pigs (6, 7). In addition, the reduction in internal pressure around bony prominences supports the hypothesized mechanism for the utility of IES for improving local blood flow to facilitate healing and preventing worsening of the injury. Clinical studies found that the device is safe, feasible, and acceptable, further emphasizing a need for a larger RCT to evaluate efficacy (8, 9). Together, these trials support IES as a promising intervention for patients with pressure injuries. The proposed study assesses the superiority of IES supplementation to the standard of care alone (offloading pressure every two hours) in decreasing the progression of sacral and ischial pressure injuries and facilitating their healing.

1.2.2 Rationale for device selection

The IES system assigned to the experimental group was selected based on existing safety and feasibility data and the absence of similar products with clinical validation in formal prospective trials.

1.2.3 Benefit Risk Assessment

The known benefits of the IES system are pain relief, reduction of visceral discomfort when sitting, reduction of spasms, and improved skin health (14). The main potential benefits are the reduction of progression of existing stage 1 and stage 2 sacral and ischial pressure injuries and the decreased time to healing which are reflected in the primary endpoint of this trial: the change in sacral and ischial pressure injury score measured over time. This benefit may, potentially, lead to shortened duration of inpatient stay and improved overall survival.

There are several potential risks associated with the use of the IES system. Prolonged use of adhesive and electrodes may lead to excessive or unusual irritation, redness, swelling, blistering, or other adverse reactions. In addition, the long-term effects of chronic electrical stimulation are unknown. These risks will be mitigated by routine inspection of electrode contact areas and underlying skin and the use of appropriate treatments for skin irritation.

The simultaneous connection of a patient to the IES system and high frequency surgical equipment may result in burns at the site of stimulator electrodes and possible damage to the device. Should a patient require surgery, the IES system will be removed prior to the procedure. The device is not to be used on patients with pacemakers or AICDs. We also exclude patients with suspected or diagnosed conditions of rhabdomyolysis or myasthenia gravis as there are no safety data in these populations.

13.3 2 STUDY OBJECTIVES AND OUTCOMES MEASURES/ ENDPOINTS

13.3.1. 2.1 Objectives

2.1.1 Primary objective

Determine the efficacy of an IES system added to the standard care of turning patients every two hours on the primary outcome of sacral and ischial pressure injury score measured over time.

2.1.2 Secondary objectives

a. Compare IES to standard care on the following secondary outcomes:
   i. Time to resolution of ulcer ii. Time to worsening of ulcer iii. Time to discharge alive iv. Mortality (30 day, 90 day, and 1 year)
b. Evaluate treatment effect heterogeneity across levels of the following patient factors:
   i. Ulcer stage at enrollment (Stage 1 or Stage 2) ii. Albumin level (>4, 3-4, 2-3, <2) measured at enrollment iii. Diagnosis of diabetes mellitus (y/n) iv. HbA1c (>10, 8-9.9, 6-7.9, <6)
   v. COVID positive during hospital stay (y/n) vi. Steroid use during study period (y/n) vii. Continuous renal replacement therapy (y/n)

4251048 - CCF LAW DEPARTMENT APPROVED AS TO FORM - ACD - 9/9/2021

viii. Pressure injury risk by Braden Score scale (Moderate 13-14, High 10-12, Very high
<9) ix. Pressure injury development
(new/established) x. BMI (<25, 25-29.9,
30-39.9) xi. Vasopressor dose at
enrollment xii. Ventilator days (0-7, 7-21,
>21)
xiii. Number of hypoxic events while using device xiv.
Highest SOFA score for the study period xv. Cumulative
fluid balance at enrollment (-2-+2L, 2-5L, 5-10L, >10L xvi.
Vasopressors used and dosages for the duration of device
usage xvii. ICU length of stay (<7 days, 7-21 days, >21
days
xviii. Number of units of packed red blood cells transfused while on the
device xix. Number of operating room procedures for which device was
turned off (0, 1-2, >3) xx. Cumulative hours in the operating room for those
procedures for which the device was turned off (0/n/a, 1-4, 4-8, >8)
xxi. OR time with the device turned off >4 hours (y/n)
xxii. Average hemoglobin over the duration of the device (<7, 7-8, 8-10,
>10) xxiii. Minimum hemoglobin while on the device
xxiv. Average blood oxygen saturation while on the device (<92, 92-95,
95-100) xxv. Number of hypotensive episodes while on the device xxvi.
Mean blood glucose while on the device

c. Compare IES to standard of care for the primary outcome in the following specific patient populations:
   i. Ventricular assist device
   ii. ECMO
   iii. Organ transplantation
d. Description and frequency of adverse events related to IES
e. Cost effectiveness analysis study comparing traditional care to IES

3 Study Design

13.3.2. Methods

This is a two-arm, prospective randomized control trial assessing whether IES combined with the standard of care (treatment) is superior to the standard of care alone (control). The study is a parallel design, adaptive, non-blinded randomized controlled trial, and uses two-sided analysis. We plan interim analyses at each 25% of the maximum N. Part of the early interim analyses (first and second) will involve reassessing the a priori assumptions on data distributions and variability use in sample size calculations and updating the maximum study size needed. Treatment effect results will be shared with the Data Safety Monitoring Board (DSMB) to determine whether the study should be ended early for either futility or having demonstrated superiority of the intervention. Patients can be entered into the protocol multiple times with independent assessments of inclusion/exclusion criteria and new consent for each enrollment. At each enrollment they will be re-randomized to either experimental or control arm. For the purpose of overall survival analysis (the only endpoint with a delayed assessment of outcome), such patients will be excluded.

4251048 - CCF LAW DEPARTMENT APPROVED AS TO FORM - ACD - 9/9/2021

3.1 Description of the Population Studied

This trial studies adult inpatients requiring intensive care with either new or established stage 1 or stage 2 sacral and ischial pressure injuries. Patients with a pacemaker/AICD, myasthenia gravis, rhabdomyolysis, gluteal skin breakdown, and unstable fractures at risk of displacement by IES are excluded. Patients with atrial or ventricular wires after cardiac surgery can be enrolled as long as they are not being paced or in the opinion of the treating physician are at high risk of requiring pacing. Prescription of neuromuscular blocking drugs is prohibited except for short term neuromuscular blockade usage for necessary procedures such as intubation or operating room visits.

3.2 Trial Duration

Subjects will be assessed for pressure injury status from point of randomization to discharge, death, or a minimum of 30 days. Device utilization and data collection will stop after 14 days in a non-ICU environment or when a total of 30 days of data collection has been met. If subjects are in the ICU longer than 30 days or when the combined total of ICU and less than 14 non-ICU days is greater than 30 days, the assessment and use of the device may continue after 30 days. Following entry into the study, participants will receive either the IES device in addition to the standard of care (treatment group) or the standard of care alone (control group). The study is expected to complete accrual within 12-18 months. Participant treatment will occur for the same amount of time as pressure injury assessment occurs as described above.
Chart review or patient contact will be used for a follow 3 and 12 months after enrollment. The total duration of the study will be approximately 24 months.

13.4 4 PATIENT SELECTION

13.4.1. 4.1 Inclusion Criteria

1. Either new or established stage 1 or 2 sacral or ischial pressure ulcer in the ICU environment.

2. Participants capable of giving informed consent, or if appropriate, participants having an acceptable individual capable of giving consent on the participant's behalf.

13.4.2. 4.2 Exclusion Criteria

1. Existing pressure injuries above Stage II and injuries classified as DTI or unstageable
2. Continuous neuromuscular blocking drugs & myasthenia gravis: may prevent the ability of electrical stimulation to induce muscular contraction
3. Unstable spinal, pelvic, or hip fractures that may be displaced by a forced contraction
4. Rhabdomyolysis

4251048 - CCF LAW DEPARTMENT APPROVED AS TO FORM - ACD - 9/9/2021

5. Presence of permanent pacemaker or AICD, and for those with external wires after cardiac surgery, those who are using or at high risk for the development of a requirement for an external pacemaker
6. Skin breakdown or malignant skin involvement over the gluteal regions that would preclude the use of surface electrodes
7. BMI $\geq$ 40

Important note: All inclusion and exclusion criteria must be adhered; no waivers will be granted.

13.4.3. 4.3 Randomization Criteria

Subjects will be randomized by covariate-adaptive randomization (block randomization) through the use of REDCap. Subjects will be stratified by initial pressure injury status into the following criteria: Stage I ulcer and Stage II ulcer. Randomization codes will be maintained until after all data are collected and analyzed.

13.4.4. 4.4 Withdrawal/Discontinuation Criteria

In accordance with the guidelines of the International Conference on Harmonization (ICH) Good Clinical Practices (GCP) and Health Canada, a patient may withdraw consent to continue participation in this study at any time. A participant may be discontinued from study treatment (but may continue to be monitored in follow-up within the trial) for any of the following reasons:

- AE or intercurrent illness that, in the opinion of the investigator or treating physician, warrants the participant's withdrawal from study treatment
- Investigator's decision
- Noncompliance with study procedure or procedure requirements
- Sponsor or Regulator Agency's discontinuation of the study
- The participant or legal representative (such as a legal guardian) withdraws consent for treatment

4.4.1 Permanent Participant Discontinuation
Participants may withdraw from the study at any time at their own request, or they may be withdrawn at any time at the discretion of the investigator or sponsor for safety or behavior reasons, or the inability of the participant to comply with the protocol required procedures. The only exception would be short-term conditions, such as an open-chested subject, where routine wound care assessment in the buttock area is temporarily deferred. Permanent study discontinuation is defined as permanent cessation of study treatment.
After study discontinuation, the patient's inpatient medical records will be reviewed at the time of discharge to identify any unexpected adverse events or outcomes. This will constitute the end of study assessment. Participants will be withdrawn from the study for the following reasons:

- Withdrawal of consent

- Surgical or other interventions that preclude the use of IES. Short term pausing of the intervention for procedures or studies that would preclude its use temporarily is acceptable.
- Death

If a patient withdraws from study and also withdraws consent for disclosure of future information, no further evaluations will be performed, and no additional data will be collected. The sponsor may retain and continue to use any data collected before the withdrawal of consent.

13.5 5 STUDY TREATMENT

13.5.1. 5.1 Treatment Allocation

5.1.1 Randomization

This study will be conducted with a 1:1 allocation ratio. Randomization will be stratified by: a) Pressure injury stage at point of entry:

Stage I
Stage II
Other pressure injury classifications are excluded.

5.1.2 Randomization or Registration Procedure

Every weekday, the clinical trial nurse, together with the staff nurse lead or delegate and / or inpatient physician lead will review all new admissions to the study site. The clinical trial nurse will screen potential subjects for eligibility. The clinical trial nurse and/or the investigators will confirm eligibility, consent, enroll and randomize the patient. Participants will be randomized by covariateadaptive randomization (block randomization). They will be stratified by the stage of pressure injury at point of consent. A statistician will generate a randomization list that will be uploaded into the Electronic Date Capture system, REDCap, for randomization. After confirming a participant's eligibility, the study staff will randomize the patient using REDCap. This is an open label study, so no blinding is required. Randomization codes will be maintained until after all the data are collected and analyzed. The patient and study staff will be informed of the randomization allocation.

5.2 Intervention

The Prelivia System™ IES system is composed of a simulator (Prelivia, Rehabtronics) and selfadhesive, non-sterile 7.5 by 10 cm surface gel electrodes (Axelgaard Pals Platinum Neurostimulation electrodes, Model 895340-4-40, Fallbrook, CA) applied directly on the skin
(Fig. 3).

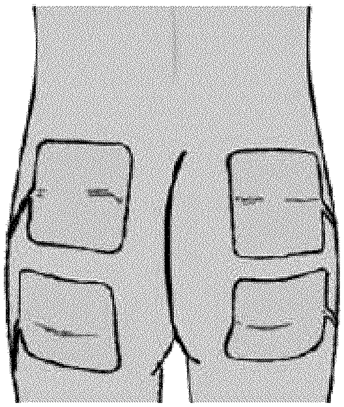

Figure 3 - The IES System. Electrodes are applied directly on the skin.

The device produces charged pulses at 30 Hz. Stimulation intensity is adjusted to generate visibly fused muscle contractions of the gluteus maximus muscles to a maximum current of 100 mA. Stimulation occurs for 10 seconds every 10 minutes. Chosen device parameters approximate those shown to induce pressure redistribution and sustained elevation in tissue oxygenation based on previous studies. Each subject will receive IES consistently 24 hours a day from entry and while in the ICU except for brief periods necessary for particular tasks in patient care (ex. Dressing changes, operating room needs). While in non-ICU environments, the device will be used while in bed and not participating in physical therapy, mobilization, toileting, and similar activities where no consistent pressure in applied to the sacral area. The study device will be used until discharge, death, resolution of the injury, or the need for surgical or other interventions that preclude the use of IES.

13.5.2. 5.3 Medications/Treatments Permitted

Apart from neuromuscular blocking drugs, all standard and investigational medical and surgical interventions are permitted during the conduct of the PROTECT study.

13.5.3. 5.4 Medications/Treatments Prohibited/Restricted

Neuromuscular blocking drugs are prohibited during the conduct of the PROTECT study. These are agents used for myasthenia gravis therapy and in the treatment of severe lung injury or clinical instability and include: succinylcholine, cis-atracurium, rocuronium, Atracurium besylate, pancuronium, vecuronium, metocurine iodide, gallamine tri-ethiodide, doxacurium, and tubocurarine. Short term usage for acute procedures is acceptable.

13.6 6 STUDY VISITS AND PROCEDURES

13.6.1. 6.1 Informed Consent

4251048 - CCF LAW DEPARTMENT APPROVED AS TO FORM - ACD - 9/9/2021

Investigators will ensure that patients are fully informed about the purpose, potential risks, and other critical issues regarding clinical trials in which they volunteer to participate. The study team will provide an appropriate informed consent form which will include all elements required by ICH, GCP and applicable regulatory requirements. The consent is given as a separate document dated and version controlled to this protocol. The informed consent documents will be submitted to ethics committees for approval. It will be emphasized in the patient information sheet that participation is voluntary and that the patient is free to refuse further participation in the protocol whenever they want to. This will not have any impact on the patient's subsequent care.
Documented informed consent will be obtained for all patients included in the study. The written informed consent form will be signed and personally dated by the patient or surrogate medical decision maker, as well as by the person who conducted the informed consent discussion. The consent document is included in the APPENDIX E.

6.2 Patient Enrollment

6.2.1 Recruitment Strategies
The inpatient nurse leaders and hospitalist physicians are collaborators and co-investigators in the study and support the conduct. The protocol has been developed in collaboration with the key people providing inpatient care at the inpatient units at the study site. All new admissions will be screened each weekday for eligible subjects.

6.2.2 Consent Procedure
This study will perform the consent procedure as follows:

1. Screening of inpatients will be performed followed by informed consent by a member of the Research Team. This will be done at the bedside in the ICU.
2. Patients admitted to the ICU who did not meet inclusion criteria immediately postoperatively or at ICU admission but meet inclusion and exclusion criteria later in the ICU stay will be requested to give informed consent by the Research Team. The Research Team will query the legal surrogate if the patient is unable to provide consent either in person or by telephone.
3. The Consent document will be approved by the IRB before the study initiation and will be used to document this process.
4. We have attached the proposed consent document for IRB review.

6.2.3 Accrual Period
The study site admits approximately 5000 new inpatients each year with an incidence of approximately 5-10% of sacral and ischial pressure ulcers. Assuming 75% eligibility and 75% consent for the study, accrual is estimated to be completed in 12 months.

6.2.4 Screening and baseline REDCap data collection:
Pre-treatment examinations will be performed within 48 hours before administration of the IES device and after having obtained the participant's written informed consent. Within 2 Days prior to randomization we will obtain:

- Signed informed consent
- Inclusion/exclusion criteria
- Demographic data, sex, age
- Critical care diagnosis
- Severity of illness screen with SOFA score on ICU day 1 and at enrollment
- Body height (cm) and weight (kg)

4251048 - CCF LAW DEPARTMENT APPROVED AS TO FORM - ACD - 9/9/2021

- Chart check for serum albumin within 14 days versus not available
- History of type II diabetes mellitus (yes/no)
- Hemoglobin A1c level
- Hemoglobin level
- Cumulative fluid balance for hospital stay
- Current steroid use > 3 days
- Active COVID-19 infection for the current hospital stay (yes/no/unknown)
- Baseline pressure injury assessment
- Pressure injury development (new, established)
- Vasoactive drug (norepinephrine, phenylephrine, dopamine, epinephrine, nitroglycerin, dopamine, dobutamine, midodrine) dosage
- Ventricular assist device or ECMO use during enrollment or for study duration
- Organ transplant recipient for the current hospital stay
- Hospital length of stay prior to enrollment

6.2.5 Randomization

- Stratified by pressure injury status

6.2.6 Data collected during study (each day of inpatient stay):

- Electrode induced skin toxicity
- Pressure injury assessment
- IES system functionality

6.2.7 End of treatment (discharge):

- Pressure injury assessment
- Electrode induced skin toxicity
- Mechanical support device during hospital stay (ventricular assist device, ECMO, renal replacement therapy)
- Organ transplant recipient for the current hospital stay
- Cumulative and daily maximum vasopressor dose for the hospital stay while on the device
- Number of ventilator days
- ICU length of stay
- Hospital length of stay from enrollment to hospital discharge or death
- Reason for discontinuation
- All inpatient treatments related to the ulcer (grafting procedures, medications, hyperbarics)
- Number of units of packed red blood cells transfused while on the device
- Number of operating room procedures for which device was turned off
- Cumulative hours in the operating room for those procedures for which the device was turned off
- OR time with the device turned off >4 hours (y/n)
- Average hemoglobin over the duration of the device
- Minimum hemoglobin while on the device
- Number of hypotensive episodes while on the device
- Average blood oxygen saturation while on the device
- Mean blood glucose while on the device
- ICU readmission during the hospital stay after enrollment

4251048 - CCF LAW DEPARTMENT APPROVED AS TO FORM - ACD - 9/9/2021

- Discharge destination

6.2.8 3 month and 1 Year Follow Up
- Chart review
- Patient readmission
- Number of wound care follow up visits for sacral or ischial pressure injury
- Discharge with wound care device or medical equipment related to sacral or ischial pressure injury

6.3 Study Visits

6.3.1 Study calendar

Protocol Activity	Screening	Inpatient Stay					Discharge	Long term Follow-up (1 year after study intervention started)
		Day 1	Day 2	Day 3	Day 4	Day 5+		
Baseline documentation								
Informed Consent	X							
Inclusion / exclusion criteria	X							
Medical history and Demographics*	X							
Height**	X							
Weight**	X							
Clinical assessments								
Physical Exam	X							
Pressure Injury Assessment								
Skin Assessment and Pressure Injury Staging	X	X	X	X	X	X	X	
Experimental Group								
Device Administration & Function Check		X	X	X	X	X	X	
Adverse Skin Events		X	X	X	X	X	X	
Other assessments								
Randomization	X							
Long term follow-up (overall survival)								X

* If available Type II Diabetes diagnosis and Serum albumin levels within past 14 days impact inclusion; If available COVID-19 test results should be noted; Neuromuscular blocking drugs are prohibited during study participation
** BMI < 35 required for inclusion

13.7 7 STATISTICAL CONSIDERATIONS

13.7.1. 7.1 Analysis

All randomized participants who have received any of the study intervention will be included in the primary endpoint analysis in a modified intention-to-treat (MITT) basis. The MITT data will be used to evaluate efficacy on the primary and secondary endpoints. The significance level will be 0.05 for all hypothesis testing, and will be controlled at 5% in case of multiple comparisons. Two-sided tests for superiority will be used throughout.

For the statistical analysis of clinical course, unstageable injuries will be assigned a score of 3 (equivalent to Stage III injuries). If a patient experiences multiple

4251048 - CCF LAW DEPARTMENT APPROVED AS TO FORM - ACD - 9/9/2021

sacral/ischial pressure injury simultaneously, the most severe injury will be used for analysis.

Primary Outcome: sacral and ischial pressure injuries (SPI) measured over time.

Timing of measurements: Patients will be assessed twice daily by bedside nurse for pressure injury location and stage from study entry to discharge or 30 days, whichever comes first. This is standard wound care evaluation protocol for nursing. Wound care nursing specialist consultation staging will be used instead of bedside nursing staging should a discrepancy occur. If SPI scores are available after discharge and within the 30 days, those data will be included in the analyses as well, even if the SPI are healed or at the worst stage.

We will assess the efficacy of an IES system added to the standard care of turning patients every two hours versus standard wound care on sacral and ischial pressure injury scores measured over time using a generalized mixed effects ordinal regression cumulative logit model which considers treatment and time (categorical) as fixed effects, subject as a random effect and accounts for 1) the ordinal nature of the data, 2) the within-subject correlation over time. The model will allow differing number of measurements and length of follow-up for patients, but will assume shorter follow-up or dropout is largely at random and not due to either improvement or worsening of SPI symptoms.

We will also assess the treatment-by-time interaction to assess whether the treatment effect is consistent over time. Given a significant interaction we will assess the treatment effect at various time points, although the primary analysis will be the treatment effect collapsed over time.

Results will be reported as a proportional odds ratio which estimates the odds of an EIS-treated patient having a higher (worse) score than a randomly chosen standard care patient. For example, an odds ratio of 0.6 would mean the treatment was associated with an estimated 40% less likely to have a higher score; correspondingly, a treated patient has a 40% better chance to have a lower (better) score compared to a standard care patient.

Displaying of results / treatment effect. We will display the distribution of scores for treatment and control over time. We will also display a forest plot of odds ratios over time and the cumulative/aggregate odds ratio collapsed over time.

Alternative statistical models will be considered as needed, or as sensitivity analyses: 1) If the generalized mixed effects model does not converge or is deemed not sufficient, an analogous generalized estimating equation (GEE) model will be used in which the correlation is adjusted for using the R matrix (within-subject correlation), either unstructured or autoregressive (AR(2)). 2) recurrent time to event model with ordinal outcome (continuation ratio approach) (Gebski, Byth, Asher and Marchner 2021). This approach models the risk for the jth event (e.g., a certain category of pressure injury at a specific f/up measurement) ignoring any previous events, but

4251048 - CCF LAW DEPARTMENT APPROVED AS TO FORM - ACD - 9/9/2021

accounting for subject as random effect, and can be modeled using an eventstratified PH regression model.

Sensitivity Analyses.

Treatment Effect Heterogeneity. We will assess treatment effect heterogeneity (IES device versus control) for the primary outcome across levels of various baseline factors outlined in section 2.1.2 using tests for interaction (treatment-by-baseline factor). Primary result will be the interaction P-value and corresponding estimate of the interaction effect. We will also test and report the treatment effect within levels of each factor.

Secondary Endpoint Analyses:

We will assess the treatment effect of IES vs standard care on time to events outcomes (time to resolution of ulcer, time to worsening of ulcer, time to discharge alive, and mortality) using Cox proportional hazards regression and reporting results as hazard ratio and 95% confidence interval, with the proportional hazards assumption tested using the treatment-by-log(time) interaction as well as graphical displays of the hazard of the outcome over time for the overlayed treatment groups. Kaplan-Meier analyses with 95% confidence bands and the log-rank test will also be used,

Exploratory Endpoints: We will assess cost effectiveness analysis within a sub-study of data generated. We will measure incremental cost-effectiveness ratios (ICERS) between traditional care and IES interventions in the treatment of pressure ulcers.

Internal Pilot Study to Re-assess assumptions on Variability and Correlation. At both the first or second interim analysis we will reassess following assumptions use in sample calculations: within-subject correlation over time, mean # measurements per subject, control proportions across stages. Reassessing these “nuisance” parameters will not affect the type I error. We will not reconsider the treatment effect of interest.

13.7.2. 7.2 Interim analysis

Interim analysis will be performed at each 25% of the planned enrollment using a group sequential design assessing the treatment effect on the primary outcome for efficacy and futility. We will use a gamma spending function with gamma parameters -4 for efficacy and -1 for futility. Stopping boundaries will be non-binding statistically.

All data and results will be submitted to the DSMB for review n an A versus B basis unless the DSMB requests to be unblinded. The DSMB will review assess any possible safety concerns and futility and advise if discontinuation is warranted.
994

4251048 - CCF LAW DEPARTMENT APPROVED AS TO FORM - ACD - 9/9/2021

13.7.3. 7.3 Sample Size Calculations

Sample size calculations are based on the primary outcome of pressure ulcer score (0,1,2,3,4) measured over time within a patient and assuming a statistical model (mixed effects ordinal regression) which accounts for the within-subject correlation over the repeated measurements. We design the study to have 90% power at the 0.05 significance level to detect a treatment effect as large or larger than the effect seen in Table A1 below (for the combined starting stages I, II). Sample size was estimated based on applying a design effect to a standard 2-group comparison for independent data; it incorporates the number of measurements and an assumed intraclass correlation (ICC) to account for within-subject correlation.

Results: Table 2 below gives total sample size for combined stages in Table 1A considering varying number of measurements per subject (10, 20, 40) and varying ICC (0.2, 0.5, 0.8). The ICC is expected to be high, so the recommended minimum sample size is 548 total (274/gp) for the overall treatment effect. However, if sufficient power is desired for each of the 2 starting strata, stage I and stage II, roughly the same sample size should be achieved for each strata (see Table 2 for Stage I and Stage II strata). Therefore, a total maximum (accounting for interim analyses) sample size of approximately 1100 patients is recommended.

Table 1A. Minimally clinically important Result: COMBINED Stages I and II

N=586 fixed, *assuming single measurement* / patient ; 668 with interims

	0	I	II	III	IV
**Intervention**	**82.5**	**2.5**	**10**	**2.5**	**0**
**Control**	**70**	**7.5**	**15**	**7.5**	**5**

Table 1B. Minimally clinically important Result: Starting at Stage I
N= 548 fixed (620 with interims)

	0	I	II	III	IV
**Intervention**	**90**	**5**	**2.5**	**2.5**	**0**
**Control**	**75**	**10**	**7.5**	**5**	**2.5**

Table 1C. Minimally clinically important Result: Starting at Stage II
N= 616 fixed (697 with interims)

	0	I	II	III	IV
**Intervention**	**85**	**0**	**12.5**	**2.5**	**0**
**Control**	**70**	**0**	**20**	**5**	**5**

4251048 - CCF LAW DEPARTMENT APPROVED AS TO FORM - ACD - 9/9/2021

**Table 2. Sample size for varying # measurements and ICC. Data = N total. Starting with N=668 total observations (Table 1A), what is required N subjects accounting for ICC and expected number of measurements/subject?**				
**Sample**	**N Measurements per patient**	**ICC**		
		**.2**	**.5**	**.8**
**Combined stages**	**10**	*188*	*368*	*548*
**Table 1A effect**	**20**	*162*	*351*	*542*
	**40**	*148*	*344*	*538*
**Stage I start**	**10**	174	342	510
**Table 1B effect**	**20**	150	326	504
	**40**	138	318	500
**Stage II start**	**10**	196	384	572
**Table 1C effect**	**20**	168	366	564
	**40**	154	358	562
ICC = intraclass correlation				

ADVERSE EVENT REPORTING

8.1 Definition of an Adverse Event

Any untoward medical occurrence, unintended disease or injury, or untoward clinical signs (including abnormal laboratory findings) in subjects, users or other persons, whether or not related to the investigational medical device which includes:

a. events related to the investigational medical device or the comparator; and

b. events related to the procedures involved

For users or other persons, this definition is restricted to events related to investigational medical devices.

8.2 Reporting, Recording and Follow-up of Adverse Events

The investigator will assess the relationship between protocol treatment and the occurrence of AEs and this assessment will be recorded in the database for adverse events. The key potential adverse events of interest are restricted to skin irritation / allergies and these skin manifestations will be scored with the International Common Terminology Criteria for Adverse Events (CTCAE), version 5.0, for adverse event reporting. Due to the non-systemic nature of the intervention, and the complexity of inpatient care and number of concurrent therapies being administered to this population, no other adverse events will be collected or reported. The investigator will follow each adverse event of interest until the event has resolved to baseline grade or better or is assessed as stable by the investigator or until the patient is discharged from hospital. During the study period, resolution of adverse events (with dates) should be documented on the Adverse Event electronic Case Report Form (eCRF)] and the patient's medical record to facilitate source data verification. If, after follow-up, return to baseline

4251048 - CCF LAW DEPARTMENT APPROVED AS TO FORM - ACD - 9/9/2021

status or stabilization cannot be established, an explanation should be recorded on the Adverse Event eCRF. *Please refer to the table below.*

Summary table for reporting of Adverse events/toxicities/SAEs:

Study Period/ Reporting procedure	AEs/toxicities	SAEs
From time of first study treatment until discharge	Yes	Yes
After Discharge	No	Yes, if found on medical chart review during one year survival assessment and assessed as related or possibly related to the treatment by the Investigator.
How to report	Source documents (e.g. study-specific worksheets) and CRF.	SAEs, unexpected, related/possibly related to be reported through an SAE form to the manufacturer, PI with copy to the IIT team within 24 hrs. REB/ Health Canada to be notified within applicable timeframe.

13.7.4. 8.3 Serious Adverse Events

Serious adverse events (SAE) as defined by the Good Clinical Practice Guideline is any untoward medical occurrence that: a. led to death
b. led to serious deterioration in the health of the subject, that either resulted in
i. a life-threatening illness or injury, or ii. a permanent impairment of a body structure or a body function, or
iii. in-patient or prolonged hospitalization, or
iv. medical or surgical intervention to prevent life-threatening illness or injury or permanent impairment to a body structure or a body function.

Only new hospitalizations will be reported as a serious adverse event and hospitalization due solely to the progression of underlying critical illness should NOT be reported as a serious adverse event.

8.3.1 Serious Adverse Event Reporting Requirements
The qualified investigator is required to report serious adverse events to the local IRB, the Cleveland Clinic IRB, and to the manufacturer within 72 hours of discovery. This includes cases in which the incident:
a. is related to a failure of the device or a deterioration in its effectiveness, or any inadequacy in its labelling or in its directions for use, and
b. has led to the death or a serious deterioration in the state of health of a patient, user or other person, or could do so were it to recur.

The preliminary report shall be submitted:
a. within 10 days after the manufacturer of a medical device becomes aware of an incident, if the incident has led to the death or a serious deterioration in the state of health of a patient, user or other person, or

4251048 - CCF LAW DEPARTMENT APPROVED AS TO FORM - ACD - 9/9/2021

b. ii. within 30 days after the manufacturer of a medical device becomes aware of an incident, if the incident has not led to the death or a serious deterioration in the state of health of a patient, user or other person, but could do so were it to recur.

The PI/Sponsor will be made aware of all Serious Adverse Events within 24 hours. All SAEs will be reported to the local research ethics board (REB) and regulatory authorities, as applicable, in accordance with local guidelines.

13.8 TO MANUFACTURER

Rehabtronics Inc.
#4352, 10230 Jasper Avenue
Edmonton, Alberta, Canada T5J 4P6
Telephone: 780-701-5167
Email: support@rehabtronics.com

13.9 TO PRINCIPAL INVESTIGATOR

Dr. Chase Donaldson, MD
Department of Intensive Care and Resuscitation; Anesthesiology Institute
Cleveland Clinic
9500 Euclid Avenue
Cleveland, OH 44195
Telephone: 216-905-6204
Email: DONALDC@ccf.org

13.10 9 MONITORING, AUDITING AND INSPECTING

The investigator/institution will permit trial-related monitoring, audits, REB, DSMB review, and regulatory inspection(s), providing direct access to paper and/or electronic documentation pertaining to the clinical study (e.g. CRFs, source documents such as hospital patient charts and investigator study files). The investigator agrees to cooperate and provide assistance at reasonable times and places with respect to any monitoring or auditing activity.

13.10.1. 9.1 Source Data

The Principal Investigator will maintain accurate source records from which the case report forms are based. The investigator agrees to allow the monitor direct access to all relevant documents. Other institutions will maintain source records locally and will make them available to the principle investigator as needed.

13.10.2. 9.2 Quality Control and Assurance

The sponsor / designee will monitor the site activity to verify that:

- The rights and well-being of human participants are protected.
- The reported trial data are accurate, complete, and verifiable from source documents.
- The conduct of the trial is in compliance with the currently approved protocol/amendment(s), with GCP, and with applicable regulatory requirements(s).

4251048 - CCF LAW DEPARTMENT APPROVED AS TO FORM - ACD - 9/9/2021

13.10.3. 9.3 Data Safety Monitoring Board

The trial activities will be monitored by the DSMB. The DSMB is independent of the investigator and is composed of representatives from Cleveland Clinic, the Department of Intensive Care and Resuscitation, and Department of ICU Nursing at the Cleveland Clinic. The PI will establish the DSMB before initiation of the clinical trial. Other participating sites may also have their own DSMB but all relevant reports will be given to the Cleveland Clinic DSMB.

13.10.4. 9.4 Deviations

The investigator is responsible to identify, document, assess and report all protocol deviations in accordance with Cleveland Clinic, REB, and HC's requirements.

13.11 <u>10 DATA COLLECTION AND DATA MANAGEMENT</u>

13.11.1. 10.1 Data Collection/Case Report Forms (CRFs)

A REDCap electronic data capture system will be used in this trial. A case report form will have to be completed for each consented patient. The site maintains a separate source of data. This data will be entered by the site into the electronic data capture system.

The investigator is ultimately responsible for the collection and timely reporting of all applicable data entered in CRFs and any other data collection forms (source documents) and ensuring they are accurate, original, attributable, complete, legible, contemporaneous, and available when required. Changes to entries made in CRF and source documents must be dated, initialed and explained (if necessary). Changes should not obscure original entry.

Data reported on the CRF that are derived from source documents should be consistent with the source documents or the discrepancies should be explained.

The CRF must be signed by the investigator or by authorized delegate to attest the data in the CRF is true.

13.12 <u>11 ADMINISTRATIVE, ETHICAL AND REGULATORY STANDARDS</u>

13.12.1. 11.1 Compliance Statement

The manufacturer will follow the principles of the Declaration of Helsinki and the Tri-Council Policy Statement (2nd Edition): Ethical Conduct for Research Involving Humans (2014) and conform to Good Clinical Practices (GCP) as set out by ISO 14155 - Clinical investigation of medical devices for human subjects.
The Investigational Medical Device will bear a label compliant with Part 3 of the Medical Devices Regulations, section 86.

11.2 Ethics
This study will be submitted to the local IRB of each study site for approval.

13.12.2. 11.4 Confidentiality and Data Protection

11.4.1 Participant Protection
The responsible investigator will ensure that this study is conducted in compliance with the protocol and in agreement with the Declaration of Helsinki. The protocol has been written, and the study will be conducted according to the ICH Harmonized Tripartite Guideline on Good Clinical Practice. HREBA.CC must approve the protocol, Informed Consent Form and any trial materials given to participants.

All potential serious breaches of GCP must be reported to Sponsor or designee immediately. A serious breach is a breach of the conditions and principles of GCP in connection with the study or the protocol, which is likely to affect, to a significant degree, the safety or physical or mental integrity of the participants of the study or the scientific value of the study.
11.4.2 Participant Identification A sequential identification number will be automatically allocated to each patient registered in the trial. This number will identify the patient and will be included on all CRFs. 11.4.3 Confidentiality of Trial Documents and Patient Records The investigators must assure that participants anonymity will be maintained and that their identities are protected from unauthorized parties. On CRFs patients should not be identified by their names, but by an identification code. Investigators should keep patients' written consent forms and a patient enrollment log at the site showing codes, names and addresses.

11.4.4 Retention of Patient Records and Study Files
The investigator must retain all study records and source documents for the maximum period required by applicable regulations and guidelines, or institution procedures, whichever is longer. 11.4.2 Participant Identification A sequential identification number will be automatically allocated to each patient registered in the trial. This number will identify the patient and will be included on all CRFs. 11.4.3 Confidentiality of Trial Documents and Patient Records The investigators must assure that participants anonymity will be maintained and that their identities are protected from unauthorized parties. On CRFs patients should not be identified by their names, but by an identification code. Investigators should keep patients' written consent forms and a patient enrollment log at the site showing codes, names and addresses.

11.4.4 Retention of Patient Records and Study Files
The investigator must retain all study records and source documents for the maximum period required by applicable regulations and guidelines, or institution procedures, whichever is longer.

13.12.3. 11.5 Protocol Registration

The sponsor has committed to the global industry position on disclosure of information about clinical trials. The International Committee of Medical Journal Editors (ICMJE) also requires trial registration as a condition of the publication of research results generated by a clinical trial. The information regarding this trial will be made publicly available on the internet at www.clinicaltrials.gov/ct2/show/NCT04328246.

13.12.4. 11.6 Protocol Amendments

Before study initiation, the investigator must have written and dated approval/favorable opinion from the Institutional Review Board (IRB) for the protocol, and consent form to be provided to participants. The investigator, Sponsor or designee should provide the IRB with reports, updates and other information (e.g., expedited safety reports, amendments, and administrative letters) according to regulatory requirements or institution procedures.
11.4.2 Participant Identification A sequential identification number will be automatically allocated to each patient registered in the trial. This number will identify the patient and will be included on all CRFs. 11.4.3 Confidentiality of Trial Documents and Patient Records. The investigators must assure that participants anonymity will be maintained and that their identities are protected from unauthorized parties. On CRFs patients should not be identified by their names, but by an identification code. Investigators should keep patients' written consent forms and a patient enrollment log at the site showing codes, names and addresses.

11.4.4 Retention of Patient Records and Study Files
The investigator must retain all study records and source documents for the maximum period required by applicable regulations and guidelines, or institution procedures, whichever is longer. All local study records and source documents must be made available to the chief study site upon request.

13.12.5. 11.5 Protocol Registration

The sponsor has committed to the global industry position on disclosure of information about clinical trials. The International Committee of Medical Journal Editors (ICMJE) also requires trial registration as a condition of the publication of research results generated by a clinical trial. The information regarding this trial will be made publicly available on the internet at www.clinicaltrials.gov/ct2/show/NCT04328246.

13.12.6. 11.6 Protocol Amendments

Before study initiation, the investigator must have written and dated approval/favorable opinion from the Institutional Review Board (IRB) for the protocol, and consent form to be provided to participants. The investigator, Sponsor or designee should provide the IRB with reports, updates and other information (e.g., expedited safety reports, amendments, and administrative letters) according to regulatory requirements or institution procedures.

13.13 12 CRITERIA FOR TERMINATION OF THE TRIAL

The investigator has the right to close their study at any time. These decisions will be taken in consultation or in discussion with the CCI DSMB. The regulatory sponsor (AHS-CCI) has the right to close this study at any time, which may be due but not limited to the following reasons:
12.1.1 Futility

4251048 - CCF LAW DEPARTMENT APPROVED AS TO FORM - ACD - 9/9/2021

If the study conduct (e.g. recruitment rate, drop-out rate, data quality, protocol compliance) does not suggest a proper completion of the study within a reasonable time frame the trial will be terminated. This will be done in consultation with the CCI DSMB.

12.1.2 Safety
If risk-benefit ratio becomes unacceptable owing to, for example,
- Safety findings from this study (e.g., SAE's)
- Results of any interim analysis
- Results of parallel clinical studies
- Results of parallel animal studies

12.1.3 Efficacy
If the results of any interim analysis demonstrate the primary objective has been attained further accrual would be unethical and the study will be closed. For any of the above closures, the following applies:
- Closures should occur only after consultation between the involved parties.
- All affected institutions (e.g. REB; competent authorities; study center(s)) must be informed as applicable according to local law.
- All study materials (except documentation that has to remain stored at site) must be returned to the sponsor. The investigator will retain all other documents until notification given by the sponsor for destruction.
- In case of a partial study closure, ongoing participants, including those in post study followup, must be taken care of in an ethical manner.

13.14 13 FINANCING AND INSURANCE

The manufacturer (Rehabtronics Inc.) will provide 40 units of the Prelivia IES System, unlimited non-sterile, adhesive electrode pads, and funding to support the clinical trial activities. This investigator-initiated trial was designed by CCF staff and will be conducted as a multi-centered trial at the hospitals listed on page 3. The Cleveland Clinic is the regulatory sponsor of the study. . Cleveland Clinic, as regulatory sponsor, will provide the insurance provided to other investigatorinitiated studies.

13.15 14 PUBLICATION POLICY

The publication of the main trial results will be written by the Principal Investigator on the basis of the final analysis and will be sent to a major scientific journal. Authors of the manuscript will include at least the Principal Investigator and any co-investigators who have i) included substantial numbers of eligible patients in the trial (by order of inclusion) or ii) contributed significantly to the design, conduct and data interpretation. Publication or presentation of study data before the publication of the primary trial endpoint may be authorized at the discretion of the Principal Investigator. The data collected during this study are confidential. Rehabtronics Inc. will be provided with a copy of all communications 30 days prior to publication and will be permitted the opportunity to suggest edits and improvements. The final content for publication will be solely the decision of the investigators.

The Principal Investigator (PI) will be responsible for ensuring participants' safety on a daily basis and for reporting Serious Adverse Events and Unanticipated Problems to

the Institutional Review Board (IRB) and FDA as required. The study statistician prepares reports that list adverse events, serious adverse events, deaths, and disease- or treatment-specific events required for monitoring body review in order to ensure good clinical care and identify any emerging trends. (adapted from https://www.niams.nih.gov/grants-funding/conducting-clinicaltrials/clinical-trial-policies-guidelines-and-templates/data-and)

13.16 15 PRINCIPAL INVESTIGATOR PROTOCOL AGREEMENT

Protocol Title: The PROTECT 2 ICU Study: Pressure Injury Treatment by Intermittent Electrical Stimulation: A Randomized, Controlled Trial Study Number: ________

By signing below, the Principal Investigator (PI) agrees to adhere to the protocol in the conduct of this study. Any change in the study must be reviewed by a formal protocol amendment procedure and the Principal Investigator will submit all changes, amendments and revisions to their Research Ethics Board. Any change to the protocol that affects patient selection, safety, or changes in the conduct of the trial will require written approval from the Cleveland Clinic IRB before implementing the change.

The Investigator will provide access to this protocol for personnel at the study center who are involved in this clinical study and will ensure that study personnel understand the protocol and have knowledge of the investigational medicinal product or intervention.

The Investigator will keep all study documents in confidence.

The Investigator(s) also agree(s) to conduct the study in accordance with the Declaration of Helsinki and the International Conference on Harmonization guidelines on Good Clinical Practice (ICH GCP).

The Principal Investigator also thereby agrees that the REB will approve all patient informed consent forms (ICF) before the study is initiated. The investigator will obtain informed consent and document this process for all patients enrolled on this study.

Chase Donaldson, MD________________________________

Principal Investigator (Print) Signature ______________________

Date (dd/mmm/yyyy) ____________________________________

13.17 16 REFERENCES

1. National Pressure Ulcer Adivsory Panel, European Pressure Ulcer Advisory Panel and Pan Pacific Pressure Injury Alliance. Prevention and Treatment of Pressure Ulcers: Quick Reference Guide. Haesler E, ed. Osbourne Park: Cambridge Media, 2014.

2. Woodbury MG, Houghton PE. (2004) Prevalence of pressure ulcers in Canadian healthcare settings. Ostomy Wound Manage 50(10), pp. 22-24.

3. Zulkowski K, Langemo D, Posthauer ME. (2005) Coming to consensus on deep tissue injury. Adv Skin Wound Care 18, pp. 28–29.

4. Krause JS, Broderick L. (2004) Patterns of recurrent pressure ulcers after spinal cord injury: Identification of risk and protective factors 5 or more years after onset. Archives of Physical Medicine & Rehabilitation 85(8), pp. 1257-64.

5. Thomas DR. (2003) The promise of topical growth factors in healing pressure ulcers. Annals of Internal Medicine 139(8), pp. 694-695.

6. Solis LR, Hallihan DP, Uwiera RR et al. (2007) Prevention of pressure-induced deep tissue injury using intermittent electrical stimulation. Journal of Applied Physiology 102(5), pp. 1992-2001.

7. Solis LR, Gyawali S, Curtis, CA et al. (2008) Changes in superficial pressure and tissue oxygenation levels due to contractions elicited by intermittent electrical stimulation for the prevention of deep tissue injury. In International functional electrical stimulation society. Freiberg, Germany.

8. Ahmetovic A, Mishahwar V, Sommer R et al. (2015) Safety and Feasibility of Intermittent Electrical Stimulation for the Prevention of Deep Tissue Injury. Adv Wound Care 4(3), pp. 192-201.

9. Kane A, Warwaruk-Rogers R, Ho C et al. (2017) A Feasibility Study of Intermittent Electrical Stimulation to Prevent Deep Tissue Injury in the Intensive Care Unit. Adv Wound Care 6(4), pp. 115-124.

10. Gyawali S, Solis L, Chong SL et al. (2011) Intermittent electrical stimulation redistributes pressure and promotes tissue oxygenation in loaded muscles of individuals with spinal cord injury. J Appl Physiol 110, pp. 246-55.

11. Solis LR, Twist E, Seres P et al. (2013) Prevention of deep tissue injury through muscle contractions induced by intermittent electrical stimulation after spinal cord injury in pigs. J Appl Physiol 114, pp. 286-96.

12. Solis LR, Gyawali S, Seres P et al. (2011) Effects of intermittent electrical stimulation on superficial pressure, tissue oxygenation, and discomfort levels for the prevention of deep tissue injury. Ann Biomed Eng 29, pp. 649-63.

13. Gyawali, S. (2009) Investigation of intermittent electrical stimulation as a potential prophylaxis against the formation of deep pressure ulcers after spinal cord injury. (Master's Thesis).

4251048 - CCF LAW DEPARTMENT APPROVED AS TO FORM - ACD - 9/9/2021

13.17.1. 17 APPENDICES

Appendix A Wound and Risk Nursing Assessment
Appendix B Wound Assessment and Treatment Record
Appendix C Pre-clinical IES Data
Appendix D Clinical participant feedback IES Data
Appendix E Consent Document

4251048 - CCF LAW DEPARTMENT APPROVED AS TO FORM - ACD - 9/9/2021

Appendix A Wound and Risk Nursing Assessment

Wounds ☐ NA ☐ NCR

☐ Go to Initial / Ongoing Wound Assessment Form

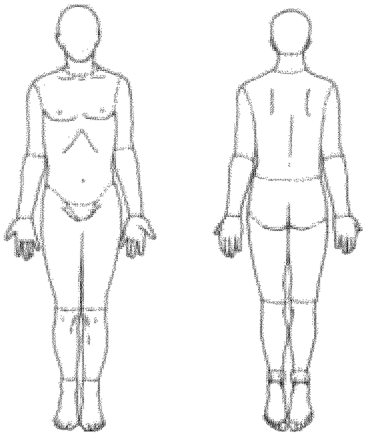

Mark all applicable areas:
X = affected area

Braden Scale for Predicting Pressure Sore Risk

**Sensory Perception**		**Moisture**	
☐ Completely limited	(1)	☐ Constantly moist	(1)
☐ Very limited	(2)	☐ Very moist	(2)
☐ Slightly limited	(3)	☐ Occasionally moist	(3)
☐ No impairment	(4)	☐ Rarely moist	(4)
**Activity**		**Mobility**	
☐ Bed fast	(1)	☐ Completely immobile	(1)
☐ Chair fast	(2)	☐ Very limited	(2)
☐ Walks occasionally	(3)	☐ Slightly limited	(3)
☐ Walks frequently	(4)	☐ No limitations	(4)
**Nutrition**		**Friction / Shear**	
☐ Very poor	(1)	☐ Problem	(1)
☐ Probably inadequate	(2)	☐ Potential problem	(2)
☐ Adequate	(3)	☐ No apparent problem	(3)
☐ Excellent	(4)		

Total Score = ________ Surface Type ________________

Risk Level: Mild (15 or 16) Moderate (13 or 14)
Severe (12 or less)

4251048 - CCF LAW DEPARTMENT APPROVED AS TO FORM - ACD - 9/9/2021

Wound Assessment and Treatment Record

May be used for multiple wounds or to record up to 4 dressing changes. Each wound is to be identified by an alphabetic indicator.	**Pressure Injury Stages**	
	1	Unresolved redness of intact skin
**Clearly circle the location of each wound and note alphabet**	2	Partial thickness skin loss

4251048 - CCF LAW DEPARTMENT APPROVED AS TO FORM - ACD - 9/9/2021

4251048 - CCF LAW DEPARTMENT APPROVED AS TO FORM - ACD - 9/9/2021

Wound Assessment and Treatment Record

**Type of Wound** (see key on front	Pressure Injury	☐ Pressure Injury<br>Stage: ☐ 1 ☐ 2<br>☐ 3 ☐ 4 ☐ U	☐ Pressure Injury<br>Stage: ☐ 1 ☐ 2<br>☐ 3 ☐ 4 ☐ U	☐ Pressure Injury<br>Stage: ☐ 1 ☐ 2<br>☐ 3 ☐ 4 ☐ U	☐ Pressure Injury<br>Stage: ☐ 1 ☐ 2<br>☐ 3 ☐ 4 ☐ U
	Other	Type:	Type:	Type:	Type:

4251048 - CCF LAW DEPARTMENT APPROVED AS TO FORM - ACD - 9/9/2021

Appendix C Pre-clinical IES Data

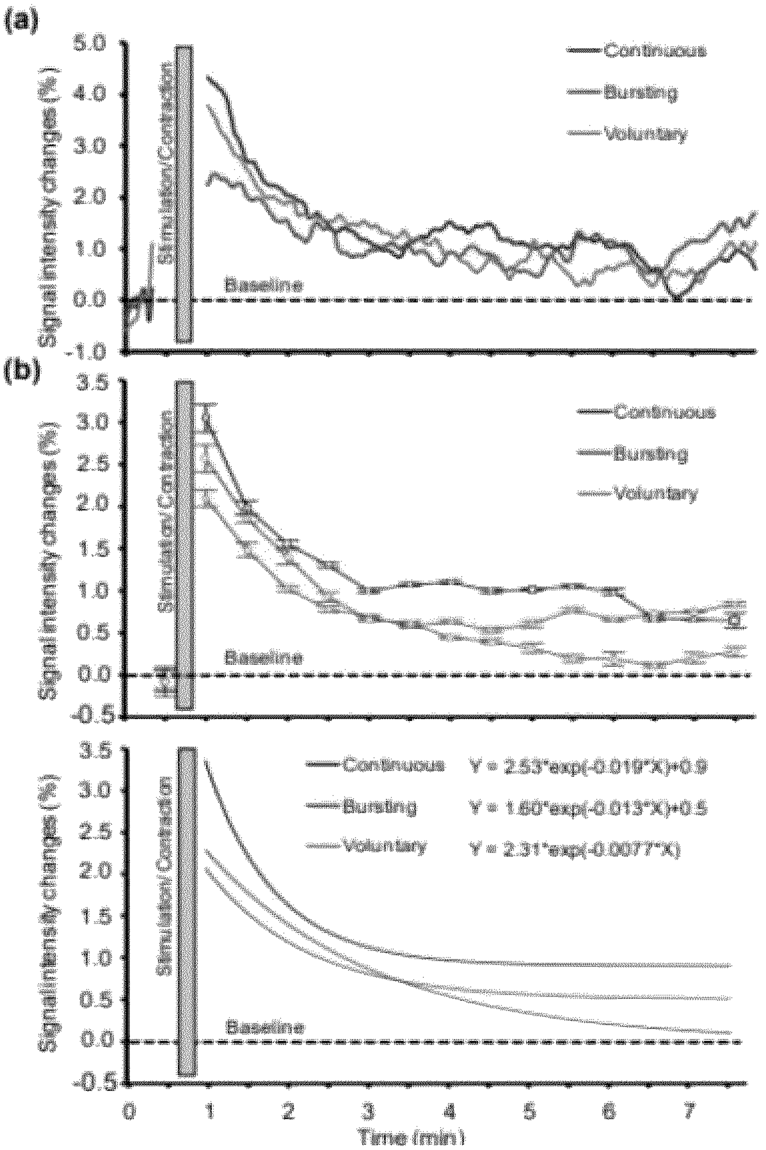

Figure 5. Changes in tissue oxygenation induced by bilateral continuous IES, bilateral bursting IES, and voluntary contractions. (a) Average change in signal intensity (%) from baseline. No data were quantified during the stimulation/ contraction period. (b) *Top*: comparison of time points after stimulation/contraction to baseline for *bilateral continuous* IES, *bilateral bursting* IES, and *voluntary* contraction protocols. Time points were grouped in 30 s time windows for comparison. (b) *Bottom*: exponential decay curves fitted for data from time points after contraction for *continuous*, *bursting*, and *voluntary* protocols.

Figure from LR Solis PhD Thesis (2013)

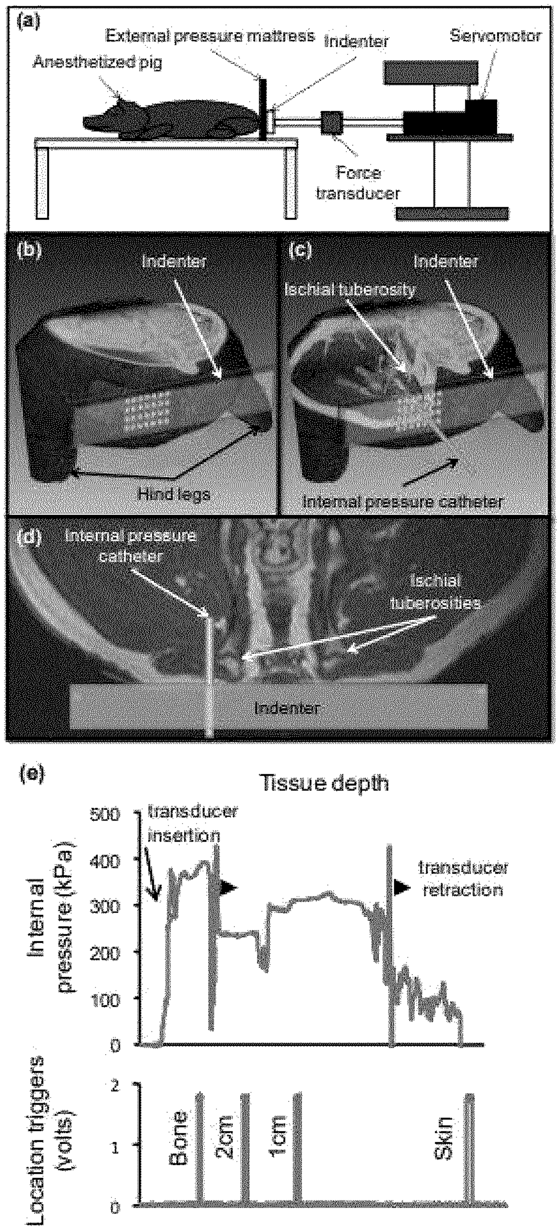

Figure 1. Experimental setup. (a) A computer controlled servo motor was used to push an indenter against the buttocks of an anesthetized pig. Loading levels equivalent to 25%, 50%, and 75% BW of each pig were applied to the buttocks. (b, c) A pressure catheter was introduced through small openings (1 mm diameter) in the indenter and into the pig's buttocks. (d) With the buttocks loaded, a map of internal pressure was formed by acquiring measurements at different dorso-ventral, medio-lateral and antero-posterior locations relative to the ischial tuberosity. (e) Top: An example of a raw trace of internal pressure recorded as the pressure transducer was retracted toward the skin from a location next to the bone. Bottom: Trigger signals recorded in conjunction with the raw pressure traces. Each trigger indicates the location, relative to the bone, from which internal pressure measurements were acquired. Arrow indicates the instance of transducer insertion toward the bone; arrow heads indicate two instances of transducer retraction in 1 cm steps through the tissue.

Figure from LR Solis PhD Thesis (2013)

4251048 - CCF LAW DEPARTMENT APPROVED AS TO FORM - ACD - 9/9/2021

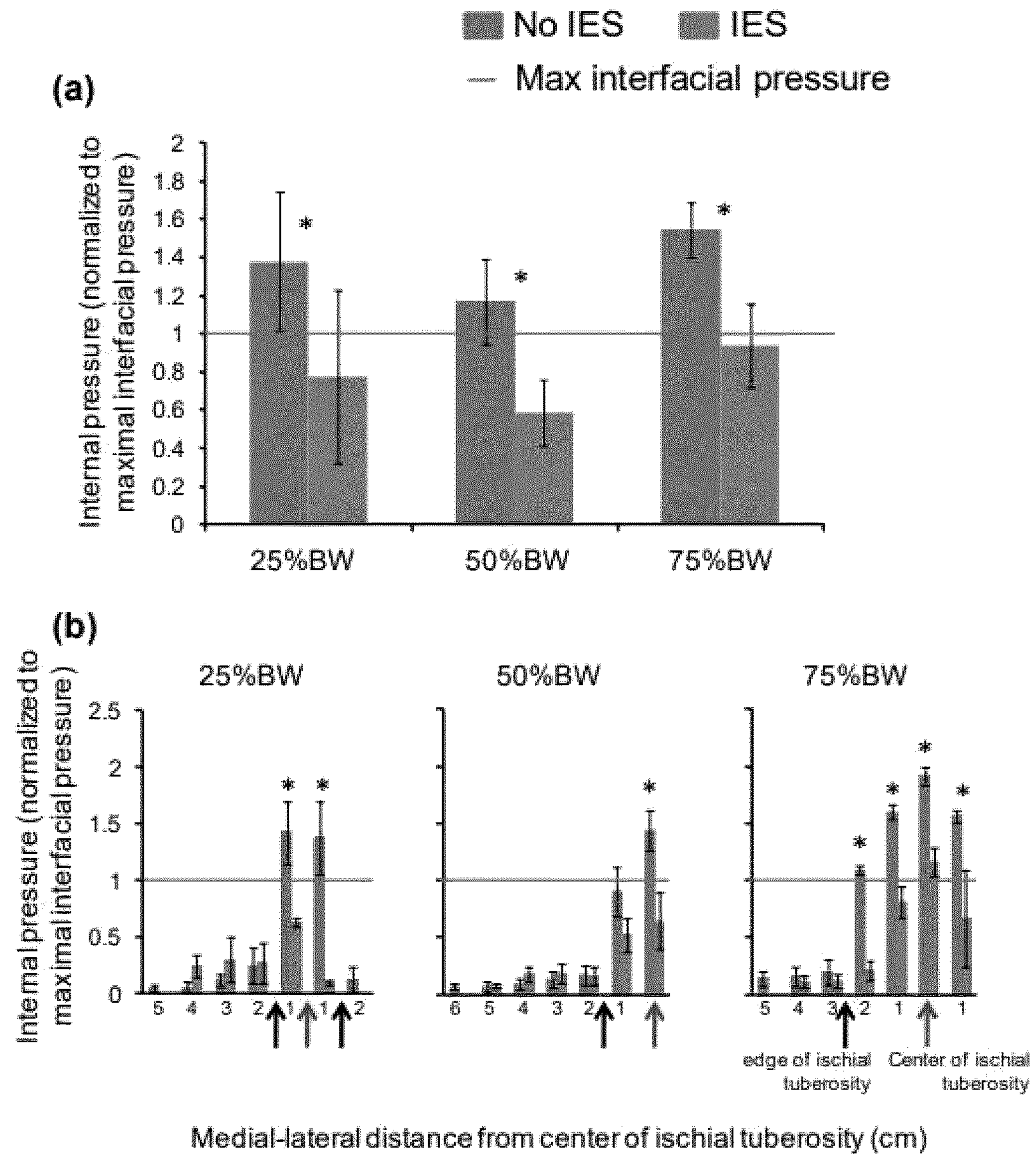

Figure 10. Statistical changes in internal pressure during IES-induced muscle contractions in intact pigs. (a) Mean internal pressure 6 standard error in the dorso-ventral plane centered on the ischial tuberosities for all levels of external loading. (b) Medio-lateral distribution of internal pressure (mean 6 standard error) relative to the ischial tuberosity in the dorso-ventral plane centered on the ischial tuberosities, for all levels of external loading, with and without IES. *Significant reduction in internal pressure during IES.

Figure from LR Solis PhD Thesis (2013)

13.17.2. Appendix D Clinical participant feedback IES Data

4251048 - CCF LAW DEPARTMENT APPROVED AS TO FORM - ACD - 9/9/2021

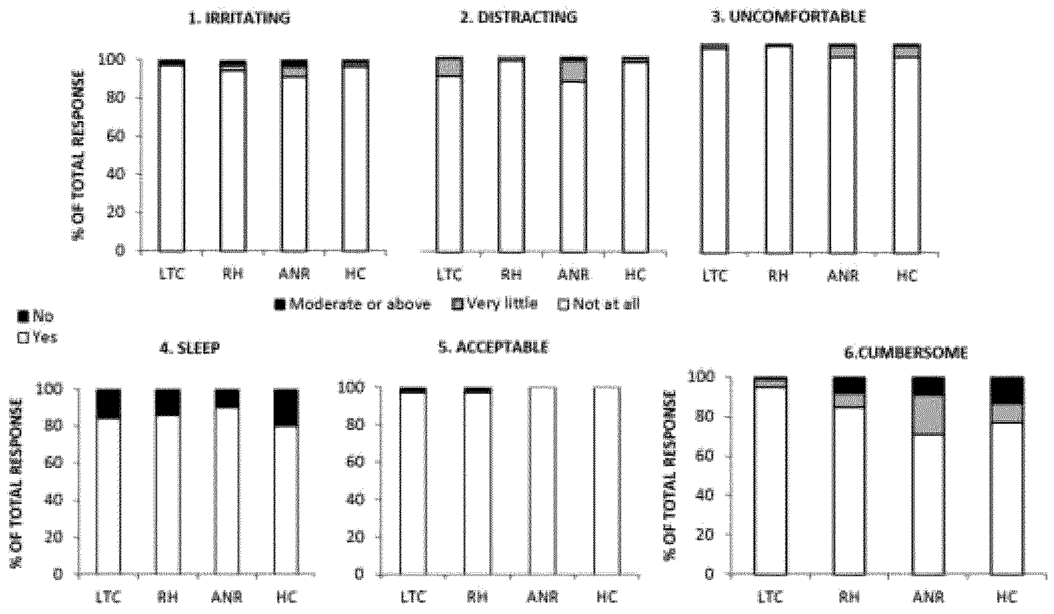

Figure 6. Participant feedback. Responses to questions regarding irritability, distraction, and discomfort are shown in the *top panels*, whereas whether the participant could sleep or find the IES acceptable or cumbersome to use are shown in the *lower panels*. Subjects' perception was generally favorable with no significant difference between different clinical settings. Abbreviations used for facility names are the same as in Figure 2.

Figure from Ahmetovic et al (2015)

13.17.3. Appendix E Consent Document

CLEVELAND CLINIC

Consent to Participate in Research(v. 08.2016)

(a) Protocol. The PROTECT 2 ICU Study: Pressure Injury Treatment by Intermittent Electrical Stimulation: A Randomized, Controlled Trial

Principal Investigator: Chase Donaldson, MD/Department of Intensive Care and Resuscitation/Anesthesiology Institute/Cleveland Clinic Foundation/Cleveland, OH 44195/216-
905-6204

Introduction to Research at Cleveland Clinic

Doctors, nurses, and medical researchers at Cleveland Clinic want to know more about the nature of disease and how to improve the lives of patients and their families. One way to learn more about diseases and their treatment is by asking patients to take part in research studies like this one, which is being led by Chase Donaldson, MD.

You are being invited to participate in this research study. Before you agree to take part though, you need to know what to expect, what risks might be involved, what benefits you might gain, and your rights as a research participant. Please take your

time and read the information in this document carefully. Ask questions about anything that is unclear to you and do not sign this form until you are sure that you understand what will happen to you as a subject in this study. If you decide to participate, you will be given a copy of this consent form to keep for your records.

(b) Information about this Study

Why am I being asked to participate?

You or your family member are being asked to participate in this study because they have been admitted to intensive care and have developed a pressure ulcer (also known as bedsores). These can develop during periods of bedrest with severe illness.

This consent form provides detailed information about the study to help you make an informed decision. Please read this document carefully and ask any questions you may have. All questions should be answered to your satisfaction before you decide whether to participate.

The study staff will tell you about timelines for making your decision. You may find it helpful to discuss the study with family and friends so that you can make the best possible decision within the given timelines.

Taking part in this study is voluntary. You may choose not to take part or, if you choose to participate, you may leave the study at any time without giving a reason. Deciding not to take part or deciding to leave the study will not result in any penalty or any loss of medical or health-related benefits to which you are entitled.

The study team, who are the researchers, will discuss this study with you and will answer any questions you may have. If you do consent to participate in this study, you will need to sign and date this consent form. You will receive a copy of the signed form.

WHAT IS THE BACKGROUND INFORMATION FOR THIS STUDY?
Pressure injuries, also known as pressure ulcers or bedsores, commonly happen to people spending time in hospital beds and people with limits in their movement and limits in their ability to feel their skin. These wounds are usually caused by prolonged sitting or lying down, and are common in adults in hospital. Bedsores most commonly happen in the buttocks and over the tailbone.

Many people hospitalized in the intensive care unit are at risk of developing pressure injuries. Pressure ulcers can be hard to treat and can lengthen your hospital stay. Sometimes pressure ulcers need surgery. The Cleveland Clinic does the standard things to reduce pressure ulcers including specialized mattresses, wheelchair cushions, and moving people every two hours. Even with these efforts, pressure ulcers sometimes happen.

In this study, a device called Prelivia is used to electrically stimulate the buttock muscles to produce muscle contractions. The device contracts muscles for 10 seconds every 10 minutes, just like healthy people do when sitting or lying down. Animal studies show that Prelivia reduces pressure, improves blood flow and oxygen to tissues, and prevents pressure ulcers. Two earlier clinical trials show that Prelivia is safe and well-accepted by patients and hospital staff.

Health Canada, the regulatory body that oversees the use of natural health products, drugs and devices in Canada, has not approved the sale or use of Prelivia to treat pressure injuries, although they have allowed its use in this study. The Food and Drug Administration (FDA) has approved this device to increase local blood flow to pressure injuries, but not for the treatment of pressure injuries.

The Cleveland Clinic Institutional Review Board which oversees the ethical acceptability of research involving humans, has reviewed and granted ethics approval for this study.

How many people are going to participate in this study?

About 240 other people are going to take part in this study at the Cleveland Clinic and Cleveland Clinic Regional Hospitals.

Who is doing this study? Members of the Critical Care team are leading the study. These include intensive care physicians, nurses, and research specialists.

The investigator on this study, Chase Donaldson MD is working with researchers from Canada to learn more about the treatment of pressure ulcers.

The study is being led by Chase Donaldson, MD from the Cleveland Clinic., and will be carried out with the assistance from ICU physicians, ICU nurses, and ICU Research specialists. This research team will work with you at the Cleveland Clinic Intensive Care Units and Cleveland Clinic Regional Hospitals.

Who can be in this study? Patients can be in the study if they are admitted to the ICU and have a stage 1 or stage 2 pressure injury (bedsore).

4251048 - CCF LAW DEPARTMENT APPROVED AS TO FORM - ACD - 9/9/2021

Participants in this study will be assigned to one of two groups.

ASSIGNMENT TO A GROUP

If you decide to participate then you will be "randomized" into one of the groups described below. Randomization means that you are put into a group by chance (like flipping a coin). There is no way to predict which group you will be assigned to. You will have an equal chance of being placed in either group. Neither you, the study staff, nor the study doctor can choose what group in which you will join.

You will be told which group you will join.

STUDY INTERVENTION

- Group 1 (Prelivia System + Usual Care): The Usual Care of turning every 2 hours to relieve pressure, established and standard treatment of pressure injuries, plus the Prelivia System.

If you are randomized into this group, you will receive the standard care to treat pressure injuries along with the experimental device. The standard care involves turning every 2 hours to relieve pressure in high risk locations (buttocks and tailbone) along with cleaning and dressing wounds.

The experimental device, Prelivia, is attached to adhesive, non-sterile electrodes that will be applied to your buttocks. The device uses electrical pulses to produce muscle contractions. The pulses will occur every 10 minutes and produce a contraction in your buttocks lasting 10 seconds. Every 12 hours, the adhesive electrodes will be replaced by nursing staff. Each time a new set of electrodes is applied, a nurse will test the device to ensure muscle contraction is taking place.

- Group 2 (Usual Care): Usual care of turning every 2 hours to relieve pressure in addition to standard medical and/or surgical treatment of pressure injuries.

If you are randomized into this group, you will receive the standard care to treat pressure injuries. The standard care involves turning every 2 hours to relieve pressure in high risk locations (buttocks and tailbone) along with cleaning and dressing wounds.

Other important information on study intervention:
If you have side effects while you are on this study, the study team may make changes to the intervention.

STUDY PROCEDURES

Standard Procedures

The following standard procedures will be done as part of this study. These procedures are being done as part of your standard nursing care and the results may be used. If the results show that you are not able to continue participating in the study, the study team will let you know.

- Physical examination
- Skin assessment and pressure injuring staging

Experimental Procedures

The following test is considered experimental and will only be done for participants in the experimental group on this study: **PROTECT2.ICU**

- Placement of the adhesive pads on the buttocks
- Prelivia device function check-assessment for a visible muscle contraction
- The device will be worn consistently while in the ICU except for necessary treatment breaks based on clinical task needs, and while in other hospital environments while in bed and not participating in other therapy activities where pressure to your sacral area will be alleviated or where the device would interfere with toileting and other similar activities.

What is the purpose of this study? What are the investigators trying to find out?

The purpose of this study is to test the effects of a new device, Prelivia, added to the usual pressure injury care for the ability to treat pressure injuries near the buttocks and tailbone during your hospital stay.

(c) Information about your Role as a Study Participant

What will I have to do? What will happen to me during this study?

If you choose to participate in this study, you will be expected to:

- Tell the study team about your current medical conditions;
- Tell the study team about all prescription and non-prescription medications and supplements, including vitamins and herbals, that you may be taking and check with the study doctor before starting, stopping or changing any of these. This is for your safety as these may interact with the intervention you receive on this study;
- Tell the study team if you are thinking about participating on another research study;
- Undergo all of the procedures described above;

How long will I be part of this study?

The study intervention will last for the remaining time of your hospital stay (until discharge).

(d) Information about the Possible Risks of Participating in the Study

It is important that you be aware of the following known risks associated with participating in this study:

You may experience side effects from participating in this study. Some side effects are known and are listed below, but there may be side effects that are not expected. You should discuss these with the study team.

The study team will watch you closely to see if you have side effects. When possible, other treatment will be given to you to make side effects less serious and more tolerable. Many side effects go away shortly after treatment is stopped but in some cases side effects can be serious. If you experience serious side effects that require treatment after you are discharged from the
Cleveland Clinic or Cleveland Clinic Regional Hospitals, it is important that you make every effort to return to the Cleveland Clinic or Cleveland Clinic Regional Hospitals where Prelivia was administered.

Because Prelivia is a new device and is only used in clinics/hospitals involved in research studies, any serious side effects of the device may be best treated by these clinics/hospitals. If you need immediate treatment and are unable to return to the clinic/hospital where Prelivia was administered, you should go to the nearest medical clinic/hospital and tell them that the study doctor should be contacted as soon as possible.

Risks and side effects related to the experimental intervention, Prelivia, being studied include: *Very likely (greater than 21% or more than 20 people in 100):*
- None

Less likely (5 – 20% or between 5 and 20 people in 100):
- Skin redness
- Difficulty falling asleep or staying asleep
- Distraction or discomfort due to stimulation

Rarely (1 – 4% or less than 5 in 100 people):
- Skin irritation, blistering, or swelling

You will receive the standard treatment for the treatment of pressure injuries. An experimental device is being added to this. This combination could change the side effects or the effectiveness of the standard treatment. This could mean that you experience more side effects than you would with only the standard treatment. If could also mean that the standard treatment does not work as expected.

The risks and side-effects of the standard or usual treatment will be explained to you as part of your standard care. These risks are not included in this consent form. A Data and Safety Monitoring Board (DSMB), an independent group of experts, will be reviewing the data throughout the conduct of the study to ensure continuing participant safety as well as scientific validity and quality of the research.

It is not always possible to know all of the risks associated with a study like this one. If any new risks are reported for this study, your doctor or someone from the study team will let you know so that you can decide if you want to continue taking part.

(e) Information about the Possible Benefits of Participating in this Study

There may not be any direct benefit to you for participating in this study. The study may benefit future patients, however, because doctors will have greater knowledge of possible treatments for pressure ulcers.

The expected benefit from taking part in this study is reduced risk and severity of pressure injuries, but there is no guarantee that the intervention will be of direct benefit to you. However, based on the results of this study, it is hoped that in the long-term, patient care can be improved.

What if I Decide not to Participate?

Participation in this study is voluntary. If you do not wish to participate in the study, you will still receive the standard therapy used by your physician in the treatment of your disease or condition.

Standard treatment for prevention and treatment of ulcers involves the nursing staffing turning you on a regular basis in the ICU.

Are there Alternatives to Participating in this Study?
Yes. You will receive the standard of care to prevent and treat pressure ulcers that involves regularly turning in bed.

Can I Stop Taking Part in the Study Once I have Enrolled?

You may withdraw from the study at any time, without any penalty to you. You will still receive treatment for your condition if you decide to stop being in the study.

If more medical information becomes available about the treatments used in this study or new treatments for your disease, you will be informed of these results as soon as possible so that you can decide if you wish to continue participating in this project.

If your doctor feels that your continued participation in the study is not in your best interest, or if you have a bad reaction to the study drugs or treatment, you may be taken off the study without your consent. Your doctor will let you know if it necessary to take you off the study.

If you withdraw from the study, it still might be necessary for the investigator to look at your medical records to follow your medical progress. If you do not want the investigator to look at your records after you've left the study, you will need to let the investigator know in writing.

(f) Confidentiality of Personal Information

How will my personal information be kept confidential?

If you decide to participate in this study, the study doctor and study staff will only collect the information they need for this study.

Records identifying you, including information collect from your medical files/records, such as your Electronic Medical Records (EMR), Netcare, charts, etc., will be kept confidential to the extent permitted by the applicable laws, will not be disclosed or made publicly available, except as described in this consent document.

Authorized representatives of the following organizations may look at your identifiable medical/clinical study records at the site where these records are held for quality assurance purposes and/or to verify that the information collected for the study is correct and follows proper laws and guidelines:

- Members of the Regulatory/Audit team at the Cleveland Clinic, for quality assurance purposes;
- The Institutional Boards of the Cleveland Clinic In which oversees the ethical conduct of this study;

Authorized representatives of the above organizations and the organization listed below may receive information related to the study from your medical/clinical study records that will be kept confidential in a secure location and may be used in current or future relevant health research. Your name or other information that may identify you will not be provided (i.e., the information will be de-identified). The records received by these organizations will be coded with a number. The key that indicates what number you have been assigned will be kept secure by the researchers directly involved with your study and will not be released.

The following organizations may receive study data:

- Rehabtronics Inc., the company that makes the Prelivia device

Any disclosure of your identifiable health information will be done in accordance with federal and state laws. The organizations listed above are required to have organizational policies and procedures to protect the information they see or receive about you, except where disclosure may be required by law. The study doctor will ensure that any personal health information collected for this study is kept in a secure and confidential location at the Cleveland Clinic as also required by law.

If the results of this study are published, your identity will remain confidential. It is expected that the information collected during the study will be used in analyses and will be published/presented to the scientific community at meetings and in journals. This information may also be used as part of a submission to regulatory authorities around the world to support the approval of this intervention.
Even though the likelihood that someone may identify you from the study data is very small, it can never be completely eliminated. Every effort will be made to keep your identifiable information confidential, and to follow the ethical and legal rules about collecting, using and disclosing this information.

A copy of the consent form that you sign to enter the study will be included in your health record/hospital chart.

For this study, some of your study data may be sent electronically through the Internet to the agency sponsoring the study, but your name will be removed from the data and any other information sent will be encrypted (scrambled) so that no one can see it except the person authorized to receive the information.

If the Principal Investigator and/or the sponsor decide to report study results in research articles or scientific presentations, no personal information about you will be revealed. The information collected about all of the study participants is grouped together without any way of identifying individuals from within that group. If the articles or presentations include your x-rays, photographs, or other images gathered during the study, it will not be possible for anyone to identify or recognize you from those pictures and your identity will not be revealed.

Who will know that I am participating in this study?

Every effort will be made to protect your privacy and maintain the confidentiality of your medical records during this study. From time to time, though, it might be necessary for certain people to check parts of your medical record to make sure that the study data are correct and complete. Whenever such checks are made, only study data is recorded, not any personal or unrelated medical information about you. The only people who may have access to your study data are the lead investigator and study staff, authorized representatives of the company sponsoring the research, auditors from the government agencies that oversee medical research, and Cleveland Clinic Research Review Board staff (the committee that oversees research involving human subjects).

Where can I find out more information about the research study? **(as applicable to certain clinical trials)**

A description of this clinical trial will be available in http://www.ClinicalTrials.gov, as required by U.S. Law. This Web site will not include information that can identify you. At most, the Web site will include a summary of the results. You can search this Web site at any time.

What will it Cost to Participate in this Study?

You are not responsible for any additional costs related to the research study that are above the cost of your routine care.

Will I be Paid to take part in this Study?

You will not be paid for taking part in this study.

What if I Think I have been Hurt Because of Being Involved in the Study?

Will I be able to get treatment for a study-related injury?

If you are hurt as a direct result of your participation in this study, you will be treated for your injury. However, the investigator and the hospital cannot provide free medical care or payment for any unfavorable outcomes resulting from participation in this research. Medical services will be offered at the usual charge and be billed to your or your insurance company in the usual manner.

[If you experience emotional distress related to your participation in this study, please contact a psychologist who can help you cope with those feelings. –

Will the drug company or sponsor of this study pay for my medical care if I am harmed by the study drug or treatment?

The sponsor, will pay for your medical treatment if any harm to you is a direct result of using the study device according to the instructions in the study procedure. The sponsor will not pay for medical expenses unrelated to the study or the study intervention, or which are related to the natural course of any underlying disease or treatment process.

Who do I contact if I think I've been harmed by the study drug or study procedure?

The Principal Investigator, Chase Donaldson, MD., is the person to contact if you think you are having a bad reaction to a study drug or treatment. You should contact him at 216-905-6204 right away if you have a bad reaction to the study drug/treatment. If you need to reach Dr. Donaldson outside of normal business hours, you should call 216-905-6204.

(g) Research Funding and Conflict of Interest Disclosure

The sponsor of this research protocol, Rehabtronics has contracted with the Cleveland Clinic to conduct this study. Financial compensation received by Cleveland Clinic above expenses, will go to a fund to support other non-funded research. Cleveland Clinic and the investigator could make a profit from participating in this study. Cleveland Clinic has taken measures to ensure that any financial interest does not result in a conflict of interest that may affect your treatment or the way the study is conducted.

(h) Your Rights as a Participant in this Research Study

Anyone who volunteers to participate in a research study is entitled to certain protections under federal law. The federal regulations make sure that you are willingly and voluntarily participating, that you have not been forced or pressured to take part, that any potential risks have been explained to you, that any potential risks to you are minimized, that you have been informed of possible benefits of being in the study, that you can leave the study at any time without penalty, and that you have been given enough information to make a decision about whether or not to take part in the study.

For questions regarding participation in this research study, including the risks, hazards, and benefits involved, you may contact the Principal Investigator, Chase

4251048 - CCF LAW DEPARTMENT APPROVED AS TO FORM - ACD - 9/9/2021

Donaldson, MD. If any questions, concerns, or complaints arise about the study in the future, you may also contact the above.

If you have questions or concerns about your rights as a research participant, you may call the
Cleveland Clinic Institutional Research Review Board at 330-344-6947. The Institutional Research Review Board is responsible for making sure that all human subjects' research at Cleveland Clinic is conducted in compliance with federal regulations.

Please understand that by signing this consent form you do not give up any of your legal rights, but indicate that you have been informed about the research study in which you are agreeing to participate. A copy of this signed consent form will be provided to you for your records.

(i) HIPAA Authorization (Privacy and Confidentiality) *(as required)*

Cleveland Clinic has rules and procedures to protect information about you. Federal and State laws also protect your privacy.

The research team working on the study will collect information about you. This includes your health information, data collected for this research study and personal identifying information including your name, address, date of birth and other identifying information.
Generally, only people on the research team will know your identity and that you are in the research study. However, sometimes other people at Cleveland Clinic may see or give out your information. These include people who review research studies including the Institutional Review Board and Research Compliance, their staff, lawyers, or other Cleveland Clinic staff.

People outside Cleveland Clinic may need to see your information for this study. Examples include government groups (such as the Food and Drug Administration), safety monitors, other hospitals in the study and the sponsor of the research and their agents. Cleveland Clinic will do our best to ensure your information is kept confidential and that only the health information which is minimally required to conduct the study is used or disclosed to people outside Cleveland Clinic; however, people outside Cleveland Clinic who receive your information may not be covered by this promise.

You do not have to give this permission to use and give out your information; however, you will not be able to participate in this research study without providing this permission by signing this consent form. The use and disclosure of your information has no expiration date.

You may cancel your permission to use and disclose your information at any time by notifying the Principal Investigator in writing**, Chase Donaldson, MD.** If you do cancel your permission to use and disclose your information, your participation in this study will end and no further information about you will be collected. Your cancellation would not affect information already collected in the study.

Informed Consent Statement

(j) Statement of Participant

I have read and have had verbally explained to me the above information and have had all my questions answered to my satisfaction. I understand that my participation is voluntary and that I may stop my participation in the study at any time. Signing this form does not waive any of my legal rights. I understand that a copy of this consent will be provided to me. By signing below, I agree to take part in this research study.

___

Subject or Authorized Representative Signature Date and Time

___

Printed name of Subject or Authorized Representative

(k) Statement of Person Conducting Informed Consent Discussion

I have discussed the information contained in this document with the participant and it is my opinion that the participant understands the risks, benefits, alternatives and procedures involved with this research study.

___

Signature of Person Obtaining Consent Date and Time

___

Printed name of Person Obtaining Consent

___

Signature of Witness (if applicable) Date and Time

___ Printed name of Witness (if applicable)

Distribution of Copies of signed consent:
1 copy to subject, 1 copy placed on medical chart, 1 copy in study coordinator/investigator records.

Ahmetović, A., Mushahwar, V. K., Sommer, R., Schnepf, D., Kawasaki, L., Warwaruk-Rogers, R., . . . Chan, K. M. (2015). Safety and Feasibility of Intermittent Electrical Stimulation for the Prevention of Deep Tissue Injury. *Advances in wound care, 4*(3), 192-201. doi:10.1089/wound.2014.0569

Ahmetovj , A., Mushahwar, V., Sommer, R. W., Schnepf, D., Kawasaki, L., Warwaruk-Rogers, R., . . . Chan, K. (2015). Safety and Feasibility of Intermittent Electrical Stimulation for the Prevention of Deep Tissue Injury. *Advances in wound care, 4 3*, 192-201.

Chou, R., Dana, T., Bougatsos, C., Blazina, I., Starmer, A. J., Reitel, K., & Buckley, D. I. (2013). Pressure ulcer risk assessment and prevention: a systematic comparative effectiveness review. *Ann Intern Med, 159*(1), 28-38. doi:10.7326/0003-4819-159-1-201307020-00006

Gebski, V, Byth, K, Asher, R, Marschner, I. Recurrent time-to-event models with ordinal outcomes. *Pharmaceutical Statistics*. 2021;20:77-92 https://doi.org/10.1002/pst.2057.

Gefen, A. (2008). How much time does it take to get a pressure ulcer? Integrated evidence from human, animal, and in vitro studies. *Ostomy Wound Manage, 54*(10), 26-28, 30-25.

Gyawali, S., Solis, L., Chong, S. L., Curtis, C., Seres, P., Kornelsen, I., . . . Mushahwar, V. K. (2011). Intermittent electrical stimulation redistributes pressure and promotes tissue oxygenation in loaded muscles of individuals with spinal cord injury. *J Appl Physiol (1985), 110*(1), 246-255. doi:10.1152/japplphysiol.00661.2010

Heagerty PJ, Zeger SL. Marginal regression models for clustered ordinal measurements. Journal of the American Statistical Association 1996; 91:1024–1036.]

Hedeker, D. (2008). Multilevel models for ordinal and nominal variables. In J. de Leeuw & E. Meijer (Eds.), Handbook of Multilevel Analysis. Springer, New York.

Kane, A., Warwaruk-Rogers, R., Ho, C., Chan, M., Stein, R., Mushahwar, V. K., & Dukelow, S. P. (2017). A Feasibility Study of Intermittent Electrical Stimulation to Prevent Deep Tissue Injury in the Intensive Care Unit. *Advances in wound care, 6*(4), 115-124. doi:10.1089/wound.2016.0686

Krause, J. S., & Broderick, L. (2004). Patterns of recurrent pressure ulcers after spinal cord injury: identification of risk and protective factors 5 or more years after onset. *Arch Phys Med Rehabil, 85*(8), 1257-1264. doi:10.1016/j.apmr.2003.08.108

Labeau, S. O., Afonso, E., Benbenishty, J., Blackwood, B., Boulanger, C., Brett, S. J., . . . the European Society of Intensive Care Medicine Trials Group, C. (2020). Prevalence, associated factors and outcomes of pressure injuries in adult intensive care unit patients: the DecubICUs study. *Intensive Care Medicine*. doi:10.1007/s00134-020-06234-9

Lima Serrano, M., González Méndez, M. I., Carrasco Cebollero, F. M., & Lima Rodríguez, J. S. (2017). Risk factors for pressure ulcer development in Intensive Care Units: A systematic review. *Med Intensiva, 41*(6), 339-346. doi:10.1016/j.medin.2016.09.003

Moore, Z., Patton, D., Avsar, P., McEvoy, N. L., Curley, G., Budri, A., . . . O'Connor, T. (2020). Prevention of pressure ulcers among individuals cared for in the prone position: lessons for the COVID-19 emergency. *Journal of Wound Care, 29*(6), 312-320. doi:10.12968/jowc.2020.29.6.312

National Pressure Ulcer Adivsory Panel, European Pressure Ulcer Advisory Panel and Pan Pacific Pressure Injury Alliance. Prevention and Treatment of Pressure Ulcers: Quick Reference Guide. (2014). Retrieved from Osbourne Park, Australia: https://www.epuap.org/wp-content/uploads/2016/10/quick-reference-guide-digital-npuapepuap-pppia-jan2016.pdf

Solis, L., Gyawali, S., Curtis, C. A., Chong, S., Thompson, R., & Mushahwar, V. (2008). *Changes in superficial pressure and tissue oxygenation levels due to contractions elicited by intermittent electrical stimulation for the prevention of deep tissue injury*.

Solis, L. R., Gyawali, S., Seres, P., Curtis, C. A., Chong, S. L., Thompson, R. B., & Mushahwar, V. K. (2011). Effects of intermittent electrical stimulation on superficial pressure, tissue oxygenation, and discomfort levels for the prevention of deep tissue injury. *Ann Biomed Eng, 39*(2), 649-663. doi:10.1007/s10439-010-0193-1

Solis, L. R., Hallihan, D. P., Uwiera, R. R., Thompson, R. B., Pehowich, E. D., & Mushahwar, V. K. (2007). Prevention of pressure-induced deep tissue injury using intermittent electrical stimulation. *J Appl Physiol (1985), 102*(5), 1992-2001. doi:10.1152/japplphysiol.01092.2006

Solis, L. R., Twist, E., Seres, P., Thompson, R. B., & Mushahwar, V. K. (2013). Prevention of deep tissue injury through muscle contractions induced by intermittent electrical stimulation after spinal cord injury in pigs. *J Appl Physiol (1985), 114*(2), 286-296. doi:10.1152/japplphysiol.00257.2012

Tang, J., Li, B., Gong, J., Li, W., & Yang, J. (2020). Challenges in the management of critical ill COVID-19 patients with pressure ulcer. *International wound journal*, 10.1111/iwj.13399. doi:10.1111/iwj.13399

Thomas, D. R. (2003). The promise of topical growth factors in healing pressure ulcers. *Ann Intern Med, 139*(8), 694-695. doi:10.7326/0003-4819-139-8-200310210-00013

4251048 - CCF LAW DEPARTMENT APPROVED AS TO FORM - ACD - 9/9/2021

Woodbury, M. G., & Houghton, P. E. (2004). Prevalence of pressure ulcers in Canadian healthcare settings. *Ostomy Wound Manage, 50*(10), 22-24, 26, 28, 30, 32, 34, 36-28.

Zulkowski, K., Langemo, D., & Posthauer, M. E. (2005). Coming to consensus on deep tissue injury. *Adv Skin Wound Care, 18*(1), 28-29. doi:10.1097/00129334-200501000-00013

4251048 - CCF LAW DEPARTMENT APPROVED AS TO FORM - ACD - 9/9/2021

Cleveland Clinic Institutional Review Board (IRB)
Federalwide Assurance (FWA 00005367)

Cleveland Clinic

Full AAHRPP Accreditation
Association for the Accreditation of Human Research Protection Programs, Inc.

December 3, 2021

Chase Donaldson, M.D.

RE: IRB #21-1009: The PROTECT 2 ICU Study: Pressure Injury Treatment by Intermittent Electrical Stimulation: A Randomized, Controlled Trial (Rehabtronics, Inc)

Dear Dr. Donaldson:

Your response dated 11/30/2021 to the prior conditional approval of your new study by the convened IRB on **11/19/21** is acceptable. Your new study is now granted full **approval for the period 12/3/2021 to 11/18/2022**.

You are approved to begin this research with the use of New Study Application 10/14/2021, 026_Section 17 - Electrical Safety & EMC, 027_ATTACHMENT 17A - IEC 60601-1 & 60601-2-10 Test Report, 028_ATTACHMENT 17B - IEC 60601-1-2 Test Report, 029_ATTACHMENT 17C - AIM 7351731 Test Report, 030_Section 18 - Bench Testing, Non-Significant Risk justification from sponosr, Significant-Risk-and-Nonsignificant-Risk-Medical-Device-Studies---Information-Sheet Complete Protocol, Prelivia Operator's Manual, FDA 510K letter, Susie Stein, RN email about IDE, 21-1009 cleaned copy ICF v 3 11-30-2021, 21-1009 tracked copy ICF v 3 11-30-2021, Response from Cory Anand re COI.

The stamp-approved consent is available online under the Approved Documents tab.

Written consent is required to document that each person has been adequately informed about this research and voluntarily agrees to participate prior to any involvement in the research.

Please note that human subjects research at Cleveland Clinic has been impacted by COVID-19. The study team is responsible for compliance with the enterprise-wide restrictions related to research. This information is available on the Intranet, including the Center for Clinical Research homepage.

Any changes or amendments require IRB review and approval prior to implementation. Unanticipated problems including adverse events and deviations are to be reported in accordance with IRB Policy 60: Adverse Events and IRB Policy 70: Unanticipated Problems.

This study may not continue beyond the approved **expiration date: 11/18/2022**. Submit a renewal application up to 30 days prior to expiration to allow sufficient time for IRB review or a completion report for closure.

Sincerely,

Bridget Howard

Bridget Howard, Esq., CIP
Executive Director, IRB and Human Research Protections

BH/jl

This letter is available online under the Correspondence tab
