# Supplementary material for: Pressure injury treatment by intermittent electrical stimulation (PROTECT-2): protocol for a multicenter randomized clinical trial
Source: Trials. 2024 May 10;25:313. doi: 10.1186/s13063-024-08085-x (PMC11083768; doi:10.1186/s13063-024-08085-x)
Supplement: Supplementary file 8 — Additional file 8.Informed consent materials. [file 13063_2024_8085_MOESM8_ESM.pdf]

The Cleveland Clinic Foundation
Consent to Participate in a Research Study

Study title: **The PROTECT 2 Study: Pressure Injury Treatment by Intermittent Electrical Stimulation: A Randomized, Controlled Trial**

Sponsor: Rehabtronics, Inc.

Principal Investigator: Chase Donaldson, MD
216-905-6204

After hours' phone contact #: 216-212-6421

KEY INFORMATION

The following is a short summary of this research study to help you decide whether or not to be a part of this research study. More detailed information is included later on in this document.

What should I know about a research study?

- Someone will explain this research study to you.
- You can choose whether or not to take part.
- You can agree to take part and then later change your mind.
- Your decision whether or not to participate will not be held against you.
- You can ask all the questions you want before you decide.

What is the purpose, procedures and duration of this study?

We invite you to take part in a research study because you or your family members have been hospitalized and have developed a pressure ulcer (also known as bedsores).
These can develop during periods of bedrest with severe illness.

This consent form provides detailed information about the study to help you make an informed decision. Please read this document carefully and ask any questions you may have. All questions should be answered to your satisfaction before you decide whether to participate.

The study staff will tell you about timelines for making your decision. You may find it helpful to discuss the study with family and friends so that you can make the best possible decision within the given timelines.

Taking part in this study is voluntary. You may choose not to take part or, if you choose to participate, you may leave the study at any time without giving a reason. Deciding not to take part or deciding to leave the study will not result in any penalty or any loss of medical or health-related benefits to which you are entitled.

The study team, who are the researchers, will discuss this study with you and will answer any questions you may have. If you do consent to participate in this study, you will need to sign and date this consent form. You will receive a copy of the signed form.

The purpose of this study is to test the effects of a new device, Prelivia, added to the usual pressure injury care for the ability to treat pressure injuries near the buttocks and tailbone during your hospital stay. The participation on this study is for 30 days or hospital discharge whichever comes first.

Prelivia is an investigational device that works by using electrical stimuli to the buttock muscles to produce muscle contractions. The device contracts muscles for 10 seconds every 10 minutes. The Food and Drug Administration (FDA) has approved this device to increase local blood flow to pressure injuries, but not for the treatment of pressure injuries.

You will be asked to participate based on "randomization" into one of the groups described below. Randomization means that you are put into a group by chance (like flipping a coin). There is no way to predict which group you will be assigned to. You will have an equal chance of being placed in either group. Neither you, the study staff, nor the study doctor can choose what group in which you will join.

You will be told which group you will join.

STUDY INTERVENTION

- Group 1 (Prelivia System + Usual Care): The Usual Care of turning every 2 hours to relieve pressure, established and standard treatment of pressure injuries, plus the Prelivia System.

If you are randomized into this group, you will receive the standard care to treat pressure injuries along with the experimental device. The standard care involves turning every 2 hours to relieve pressure in high risk locations (buttocks and tailbone) along with cleaning and dressing wounds.

The experimental device, Prelivia, is attached to adhesive, non-sterile electrodes that will be applied to your buttocks. The device uses electrical pulses to produce muscle contractions. The pulses will occur every 10 minutes and produce a contraction in your buttocks lasting 10 seconds. Every 12 hours, the adhesive electrodes will be replaced by nursing staff. Each time a new set of electrodes is applied, a nurse will test the device to ensure muscle contraction is taking place. Also, you will be asked every day about possible disturbances in your sleep, discomfort or any electrical shock sensation through a questioner.

- Group 2 (Usual Care): Usual care of turning every 2 hours to relieve pressure in addition to standard medical and/or surgical treatment of pressure injuries.

If you are randomized into this group, you will receive the standard care to treat pressure injuries. The standard care involves turning every 2 hours to relieve pressure in high risk locations (buttocks and tailbone) along with cleaning and dressing wounds.

Your participation in the research will last for the remaining time of your hospital stay (until discharge).

More detailed information can be found under the section labeled: “Information on the Research.”

Why might you choose not to participate in this research study?

Risks and side effects related to the experimental intervention, Prelivia, being studied include:

- Skin redness
- Difficulty falling asleep or staying asleep
- Distraction or discomfort due to stimulation
- Skin irritation, blistering, or swelling
- Mild electric shock sensation

More detailed information can be found under the section labeled: “Information on the Research.”

Why might you choose to volunteer for this study?

There may not be any direct benefit to you for participating in this study. The study may benefit future patients, however, because doctors will have greater knowledge of possible treatments for pressure ulcers.

We cannot know if you will have any benefit as a result of your participation in the study; it is possible the study treatment reduces the risk and severity of pressure injuries, which may give you relief from some symptoms or improve your quality of life. However, it is possible that your condition could worsen. Your participation in this study will help to obtain information about treating subjects with pressure injuries.

What are my other choices if I do not take part in this study?

If you decide not to take part in this study, you have other choices. For example:

- You will still receive the standard treatment for prevention and treatment of ulcers.

More detailed information can be found under the section labeled: “Information on the Research.”

DETAILED INFORMATION

The following is more detailed information about this study in addition to the information listed above.

1. INFORMATION ON THE RESEARCH

Why is the research study being done?

Pressure injuries, also known as pressure ulcers or bedsores, commonly happen to people spending time in hospital beds and people with limits in their movement and limits in their ability to feel their skin. These wounds are usually caused by prolonged sitting or lying down, and are common in adults in hospital. Bedsores most commonly happen in the buttocks and over the tailbone.

Many people hospitalized are at risk of developing pressure injuries. Pressure ulcers can be hard to treat and can lengthen your hospital stay. Sometimes pressure ulcersneed surgery. The Cleveland Clinic does the standard things to reduce pressure ulcers including specialized mattresses, wheelchair cushions, and moving people every two hours. Even with theseefforts, pressure ulcers sometimes happen.

Health Canada, the regulatory body that oversees the use of natural health products, drugs and devices in Canada, has not approved the sale or use of Prelivia to treat pressure injuries, although they have allowed its use in this study. The Food and Drug Administration (FDA) has approved this device to increase local blood flow to pressure injuries, but not for the treatment of pressure injuries. No surgical procedure and/or anesthesia is required for the use of Prelivia

How Many People Will Take Part in this Study?

Approximately 240 people are going to take part in this study at the Cleveland Clinic. This is a Multi-Center study and the approximate enrollment is about548 to 1100.

What is involved if you decide to take part in this research study?

If you decide to participate then you will be "randomized" into one of the groups described below. Randomization means that you are put into a group by chance (like flipping a coin). There is no way to predict which group you will be assigned to. You will have an equal chance of being placed in either group. Neither you, the study staff, nor the study doctor can choose what group in which you will join.

- You will be told which group you will join.

STUDY INTERVENTION

Group 1 (Prelivia System + Usual Care): The Usual Care of turning every 2 hours to relieve pressure, established and standard treatment of pressure injuries, plus the Prelivia System.

If you are randomized into this group, you will receive the standard care to treat pressure injuries along with the experimental device, Prelivia. The standard care involves turning every 2 hours to relieve pressure in high risk locations (buttocks and tailbone) along with cleaning and dressing wounds.

Group 2 (Usual Care): Usual care of turning every 2 hours to relieve pressure in addition to standard medical and/or surgical treatment of pressure injuries.

If you are randomized into this group, you will receive the standard care to treat pressure injuries. The standard care involves turning every 2 hours to relieve pressure in high risk locations (buttocks and tailbone) along with cleaning and dressing wounds.

Other important information on study intervention:
If you have side effects while you are on this study, the study team may make changes to the intervention.

Does the Research Involve Drugs/Devices?

Yes, the experimental device, Prelivia, is attached to adhesive, non-sterile electrodes that will be applied to your buttocks. The device uses electrical pulses to produce muscle contractions. The pulses will occur every 10 minutes and produce a contraction in your buttocks lasting 10 seconds. Every 12 hours, the adhesive electrodes will be replaced by nursing staff. Each time a new set of electrodes is applied, the nurse who is taking standard care of you will test the device to ensure muscle contraction is taking place.

How will my data be used?

Your data may be sent outside of the Cleveland Clinic for research purposes. Examples include government groups (such as the Food and Drug Administration), safety monitors, other hospitals in the study and the sponsor of the research and their agents. Cleveland Clinic will take steps to ensure your information is kept confidential and that only the health information which is minimally required to conduct the study is used or disclosed to people outside Cleveland Clinic; however, people outside Cleveland Clinic who receive your information may not be covered by this promise.

2. ALTERNATIVES

What are the alternatives to participation in the research study?

Participation in this study is voluntary. If you do not wish to participate in the study, you will still receive the standard therapy used by your physician in the treatment of your disease or condition.

3. RISKS

What are the risks of participating in the research study?

It is important that you be aware of the following known risks associated with participating in this study:
You may experience side effects from participating in this study. Some side effects are known and

are listed below, but there may be side effects that are not expected. You should discuss these with the study team.

The study team will watch you closely to see if you have side effects. When possible, other treatment will be given to you to make side effects less serious and more tolerable. Many side effects go away shortly after treatment is stopped but in some cases side effects can be serious.
If you experience serious side effects that require treatment after you are discharged from the Cleveland Clinic Hospital, it is important that you make efforts to return to the Cleveland Clinic Hospital where Prelivia was administered.

Because Prelivia is a new device and is only used in clinics/hospitals involved in research studies, any serious side effects of the device may be best treated by these clinics/hospitals. If you need immediate treatment and are unable to return to the clinic/hospital where Prelivia was administered, you should go to the nearest medical clinic/hospital and tell them that the study doctor should be contacted as soon as possible.

Risks and side effects related to the experimental intervention, Prelivia, being studied include:
As in any study, side effects can be temporary or permanent, mild or severe, and, especially in this study, would be unlikely to be fatal.

Likely (5 – 20% or between 5 and 20 people in 100):
- Skin redness
- Difficulty falling asleep or staying asleep
- Distraction or discomfort due to stimulation

Rarely (1 – 4% or less than 5 in 100 people):
- Skin irritation, blistering, or swelling
- Mild electric shock sensation

You will receive the standard treatment for the treatment of pressure injuries. An experimental device is being added to this if you are randomized to the device arm. This combination could change the side effects or the effectiveness of the standard treatment. This could mean that you experience more side effects than you would with only the standard treatment. If could also mean that the standard treatment does not work as expected.

The risks and side-effects of the standard or usual treatment will be explained to you as part of your standard care. These risks of the standard treatment are not included in this consent form.

A Data and Safety Monitoring Board (DSMB), an independent group of experts, will be reviewing the data throughout the conduct of the study to ensure continuing participant safety as well as

scientific validity and quality of the research.

It is not always possible to know all of the risks associated with a study like this one. If any new risks are reported for this study, your doctor or someone from the study team will let you know so that you can decide if you want to continue taking part.

Confidentiality Risks

There is a potential risk of loss of confidentiality of your data. Efforts will be made to keep your information confidential through the use of the following safeguards: If you decide to participate in this study, the study doctor and study staff will only collect the information they need for this study. Records identifying you, including information collect from your medical files/records, such as your Electronic Medical Records (EMR), Epic, charts, etc., will be kept confidential to the extent permitted by the applicable laws, will not be disclosed or made publicly available, except as described in this consent document. Authorized representatives of the following organizations may look at your identifiable medical/clinical study records at the site where these records are held for quality assurance purposes and/or to verify that the information collected for the study is correct and follows proper laws and guidelines:

- The Institutional Board of the Cleveland Clinic in which oversees the ethical conduct of this study;

Authorized representatives of the above organizations and the organization listed below may receive information related to the study from your medical/clinical study records that will be kept confidential in a secure location and may be used in current or future relevant health research. Your name or other information that may identify you will not be provided (i.e., the information will be de-identified). The records received by these organizations will be coded with a number. The key that indicates what number you have been assigned will be kept secure by the researchers directly involved with your study and will not be released.
The following organizations may receive study data:

- Rehabtronics Inc., the company that makes the Prelivia device
- FDA as part of the clinical trial

Any disclosure of your identifiable health information will be done in accordance with federal and state laws. The organizations listed above are required to have organizational policies and procedures to protect the information they see or receive about you, except where disclosure may be required by law. The study doctor will ensure that any personal health information collected for this study is kept in a secure and confidential location at the Cleveland Clinic as also required by law.

If the results of this study are published, your identity will remain confidential. It is expected that the information collected during the study will be used in analyses and will be published/presented to the scientific community at meetings and in journals. This information may also be used as part of a submission to regulatory authorities around the world to support the approval of this intervention.
Even though the likelihood that someone may identify you from the study data is very small, it can never be completely eliminated. Efforts will be made to keep your identifiable information

confidential, and to follow the ethical and legal rules about collecting, using and disclosing this information.

Your consent to participate in this study will be stored and documented in your medical record / hospital record.
For this study, some of your study data may be sent electronically through the Internet to the agency sponsoring the study, but your name will be removed from the data and any other information sent will be encrypted (scrambled) so that no one can see it except the person authorized to receive the information.

If the Principal Investigator and/or the sponsor decide to report study results in research articles or scientific presentations, no personal information about you will be revealed. The information collected about all of the study participants is grouped together without any way of identifying individuals from within that group. If the articles or presentations include your x-rays, photographs, or other images gathered during the study, it will not be possible for anyone to identify or recognize you from those pictures and your identity will not be revealed.

4. BENEFITS
What are possible benefits of participating in the research?

There may not be any direct benefit to you for participating in this study. The study may benefit future patients, however, because doctors will have greater knowledge of possible treatments for pressure ulcers.

The possible benefit from taking part in this study is reduced risk and severity of pressure injuries, but there is no guarantee that the intervention will be of direct benefit to you. However, based on the results of this study, it is hoped that in the long-term, patient care can be improved.

5. COSTS
Are there any costs to you if you participate in this study?

- There is no cost to you to be in this research study.

The study device will be provided free of charge to you or your insurance while you are participating in this study. It is possible that the study device will need to be replaced or its batteries changed. During the study, the sponsor will pay for the costs associated with replacements.

6. PAYMENT
Are there any payments to you if you participate in this study?

- You will not be paid for taking part in this study.

7. RESEARCH RELATED INJURY

What will happen if you are injured as a result of taking part in the research?

In the event you suffer a research related injury as a result of being in this study, Cleveland Clinic will provide appropriate medical treatment for such injury in a timely manner. Provision of such medical treatment does not imply any negligence or other wrongdoing on the part of Cleveland Clinic or any of the physicians or other personnel involved in the study. If you believe that you have been injured as a result of participating in the study, please immediately contact your Cleveland Clinic study doctor even if you may have already been seen or treated by another doctor. If you are seen or treated by a doctor other than the study doctor, you should inform such doctor that you are in this study and, if possible, take this document with you as it may help such doctor treat you.

In the event you suffer a research related injury as a result of being in this study, the costs for medical treatment may be billed to you or your medical insurance plan, if applicable. Medical insurance plans may or may not cover costs for medical treatment of research-related injuries. If you have insurance, you should check with your medical insurance plan before deciding to participate in this research study. In the event your medical insurance plan covers some or all of the treatment costs, you may still be responsible for co-pays or deductibles as required by your medical insurance plan.

Cleveland Clinic has not set aside any money to pay you or to pay for your treatment if you suffer a research related injury as a result of being in the study. There are no plans for Cleveland Clinic to provide other forms of compensation (such as lost wages, pain and suffering, or other direct or indirect losses) to you for research related injuries. You are not waiving any legal rights by signing this form, including the right to seek compensation for an injury. Further information about research related injury is available by contacting the Institutional Review Board at (216) 444-2924.

8. PRIVACY AND CONFIDENTIALITY

What will happen to your information that is collected for this research?

Cleveland Clinic may share your study information, without anyone knowing that it is related to you specifically, with others or use it to research projects not listed in this form. Your data may be stored and shared for future research without additional informed consent if identifiable private information, such as your name and medical record number, are removed. If your identifying information is removed from your data, we will no longer be able to identify and destroy them.

Study results may be shared in medical journals, at scientific meetings, and in other media without your identifying information. Your records will be confidential and your identity will not be shared in medical journals, at scientific meetings, and in other media without your express consent.

Authorization to Use/Disclose Protected Health Information

Cleveland Clinic has rules and procedures to protect information about you. Federal and State laws also protect your privacy.

The research team working on the study will collect information about you. This includes your health information, data collected for this research study and personal identifying information including your name, address, date of birth and other identifying information.

Generally, only people on the research team will know your identity and that you are in the research study. However, sometimes other people at Cleveland Clinic may see or give out your information. These include people who review research studies including the Institutional Review Board and Research Compliance, their staff, lawyers, or other Cleveland Clinic staff.

People outside Cleveland Clinic may see your information for this study. Examples include government groups (such as the Food and Drug Administration), safety monitors, other hospitals in the study and the sponsor of the research and their agents. Cleveland Clinic will take steps to ensure your information is kept confidential and that only the health information which is minimally required to conduct the study is used or disclosed to people outside Cleveland Clinic; however, people outside Cleveland Clinic who receive your information may not be covered by this promise.

You do not have to give this permission to use and give out your information; however, you will not be able to participate in this research study without providing this permission by signing this consent form. The use and disclosure of your information has no expiration date.

You may cancel your permission to use and disclose your information at any time by notifying the Principal Investigator in writing, Chase Donaldson, MD; Cleveland Clinic Foundation, 9500 Euclid Avenue Cleveland, OH 44195. If you do cancel your permission to use and disclose your information, your participation in this study will end and no further information about you will be collected. Your cancellation would not affect information already collected in the study.

Clinical Trials Registration

A description of this clinical trial will be available on http://www.ClinicalTrials.gov, as required by U. S. law. This Website will not include information that can identify you. At most, the Website will include a summary of the results. You can search the Website at any time.

9. QUESTIONS

Who do you call if you have any questions or problems?

If you have any questions or concerns about the research, or develop a research-related problem, you should contact Chase Donaldson, MD at 216 905-6204. During non-business hours, weekends and holidays, please contact 216 212-6421. If you have questions about your rights as a research subject, you should contact the Institutional Review Board at (216) 444-2924.

10. VOLUNTARY PARTICIPATION

What are your rights as a research participant?

Taking part in this study is voluntary. You will be told of any new, relevant information from the research that may affect your health, welfare, or willingness to continue in this study. You may choose not to take part or may leave the study at any time. Withdrawing from the study will not result in any penalty or loss of benefits to which you are entitled. If you decide to withdraw from the study you should discuss with your study doctor your decision to ensure a safe withdrawal.

If you leave the study early, Cleveland Clinic may use or give out your health information that it has already collected if the information is needed for this study or any follow-up activities.

Can I Stop Taking Part in the Study Once I have Enrolled?

Subject's Withdrawal from the Study – You may withdraw from the study at any time, without any penalty to you. You will still receive treatment for your condition if you decide to stop being in the study.

If more medical information becomes available about the treatments used in this study or new treatments for your disease, you will be informed of these results as soon as possible so that you can decide if you wish to continue participating in this project.

Sponsor's and/or PI's Withdrawal of Subject – If your doctor feels that your continued participation in the study is not in your best interest, or if you have a bad reaction to the study drugs or treatment, you may be taken off the study without your consent. Your doctor will let you know if it necessary to take you off the study.

Study team or sponsor long term follow up – If you withdraw from the study, it still might be necessary for the investigator to look at your medical records to follow your medical progress. If you do not want the investigator to look at your records after you've left the study, you will need to let the investigator know in writing.

11. SIGNATURES
Statement of Participant

I have read and have had verbally explained to me the above information and have had all my questions answered to my satisfaction. I understand that my participation is voluntary and that I may stop my participation in the study at any time. Signing this form does not waive any of my legal rights. I understand that a copy of this consent will be provided to me. By signing below, I agree to take part in this research study.

____
Printed name of Participant

____ ____________________
Participant Signature Date

Legally Authorized Representative (LAR)
You have had the above research study explained to you and as an individual likely to understand the subject's situation and acting in their best interest, you give your permission (or authorize) for participation in this research

____
Printed Name of Subject

____ ___________________________
Printed Name of LAR

____ ___________________________
LAR Signature Date

____
Printed Name of Witness (if needed)

Signature of Witness (if needed) Date

Statement of Person Conducting Informed Consent Discussion
I have discussed the information contained in this document with the participant and it is my opinion that the participant understands the risks, benefits, alternatives and procedures involved with this research study.

____
Printed name of person obtaining consent

____ _____________________
Signature of person obtaining consent Date
