# Supplementary material for: Pressure injury treatment by intermittent electrical stimulation (PROTECT-2): protocol for a multicenter randomized clinical trial
Source: Trials. 2024 May 10;25:313. doi: 10.1186/s13063-024-08085-x (PMC11083768; doi:10.1186/s13063-024-08085-x)
Supplement: Supplementary file 6 — Additional file 6. IRB approval/ethical approval. [file 13063_2024_8085_MOESM6_ESM.pdf]

Cleveland Clinic

Full AAHRPP Accreditation

Association for the Accreditation of Human Research Protection Programs, Inc.

December 3, 2021

Chase Donaldson, M.D.

RE: IRB #21-1009: The PROTECT 2 ICU Study: Pressure Injury Treatment by Intermittent Electrical Stimulation: A Randomized, Controlled Trial (Rehabtronics, Inc)

Dear Dr. Donaldson:

Your response dated 11/30/2021 to the prior conditional approval of your new study by the convened IRB on **11/19/21** is acceptable. Your new study is now granted full **approval for the period 12/3/2021 to 11/18/2022**.

You are approved to begin this research with the use of New Study Application 10/14/2021, 026_Section 17 - Electrical Safety & EMC, 027_ATTACHMENT 17A - IEC 60601-1 & 60601-2-10 Test Report, 028_ATTACHMENT 17B - IEC 60601-1-2 Test Report, 029_ATTACHMENT 17C - AIM 7351731 Test Report, 030_Section 18 - Bench Testing, Non-Significant Risk justification from sponosr, Significant-Risk-and-Nonsignificant-Risk-Medical-Device-Studies---Information-Sheet Complete Protocol, Prelivia Operator's Manual, FDA 510K letter, Susie Stein, RN email about IDE, 21-1009 cleaned copy ICF v 3 11-30-2021, 21-1009 tracked copy ICF v 3 11-30-2021, Response from Cory Anand re COI.

The stamp-approved consent is available online under the Approved Documents tab.

Written consent is required to document that each person has been adequately informed about this research and voluntarily agrees to participate prior to any involvement in the research.

Please note that human subjects research at Cleveland Clinic has been impacted by COVID-19. The study team is responsible for compliance with the enterprise-wide restrictions related to research. This information is available on the Intranet, including the Center for Clinical Research homepage.

Any changes or amendments require IRB review and approval prior to implementation. Unanticipated problems including adverse events and deviations are to be reported in accordance with IRB Policy 60: Adverse Events and IRB Policy 70: Unanticipated Problems.

This study may not continue beyond the approved **expiration date: 11/18/2022**. Submit a renewal application up to 30 days prior to expiration to allow sufficient time for IRB review or a completion report for closure.

Sincerely,

Bridget Howard

Bridget Howard, Esq., CIP
Executive Director, IRB and Human Research Protections

BH/jl

This letter is available online under the Correspondence tab
